# An *in silico* investigation on the binding site preference of PD-1 and PD-L1 for designing antibodies for targeted cancer therapy

**DOI:** 10.1371/journal.pone.0304270

**Published:** 2024-07-25

**Authors:** Sarah Abdolmaleki, Mazdak Ganjalikhani hakemi, Mohamad Reza Ganjalikhany

**Affiliations:** 1 Department of Cell and Molecular Biology & Microbiology, University of Isfahan, Isfahan, Iran; 2 Regenerative and Restorative Medicine Research Center (REMER), Research Institute for Health Sciences and Technologies (SABITA), Istanbul Medipol University, Istanbul, Turkey; 3 Department of Immunology, Faculty of Medicine, Isfahan University of Medical Sciences, Isfahan, Iran; National Institute of Health, INDIA

## Abstract

Cancer control and treatment remain a significant challenge in cancer therapy and recently immune checkpoints has considered as a novel treatment strategy to develop anti-cancer drugs. Many cancer types use the immune checkpoints and its ligand, PD-1/PD-L1 pathway, to evade detection and destruction by the immune system, which is associated with altered effector function of PD-1 and PD-L1 overexpression on cancer cells to deactivate T cells. In recent years, mAbs have been employed to block immune checkpoints, therefore normalization of the anti-tumor response has enabled the scientists to develop novel biopharmaceuticals. *In vivo* affinity maturation of antibodies in targeted therapy has sometimes failed, and current experimental methods cannot accommodate the accurate structural details of protein-protein interactions. Therefore, determining favorable binding sites on the protein surface for modulator design of these interactions is a major challenge. In this study, we used the *in silico* methods to identify favorable binding sites on the PD-1 and PD-L1 and to optimize mAb variants on a large scale. At first, all the binding areas on PD-1 and PD-L1 have been identified. Then, using the RosettaDesign protocol, thousands of antibodies have been generated for 11 different regions on PD-1 and PD-L1 and then the designs with higher stability, affinity, and shape complementarity were selected. Next, molecular dynamics simulations and MM-PBSA analysis were employed to understand the dynamic, structural features of the complexes and measure the binding affinity of the final designs. Our results suggest that binding sites 1, 3 and 6 on PD-1 and binding sites 9 and 11 on PD-L1 can be regarded as the most appropriate sites for the inhibition of PD-1-PD-L1 interaction by the designed antibodies. This study provides comprehensive information regarding the potential binding epitopes on PD-1 which could be considered as hotspots for designing potential biopharmaceuticals. We also showed that mutations in the CDRs regions will rearrange the interaction pattern between the designed antibodies and targets (PD-1 and PD-L1) with improved affinity to effectively inhibit protein-protein interaction and block the immune checkpoint.

## Introduction

**Fig 1:**
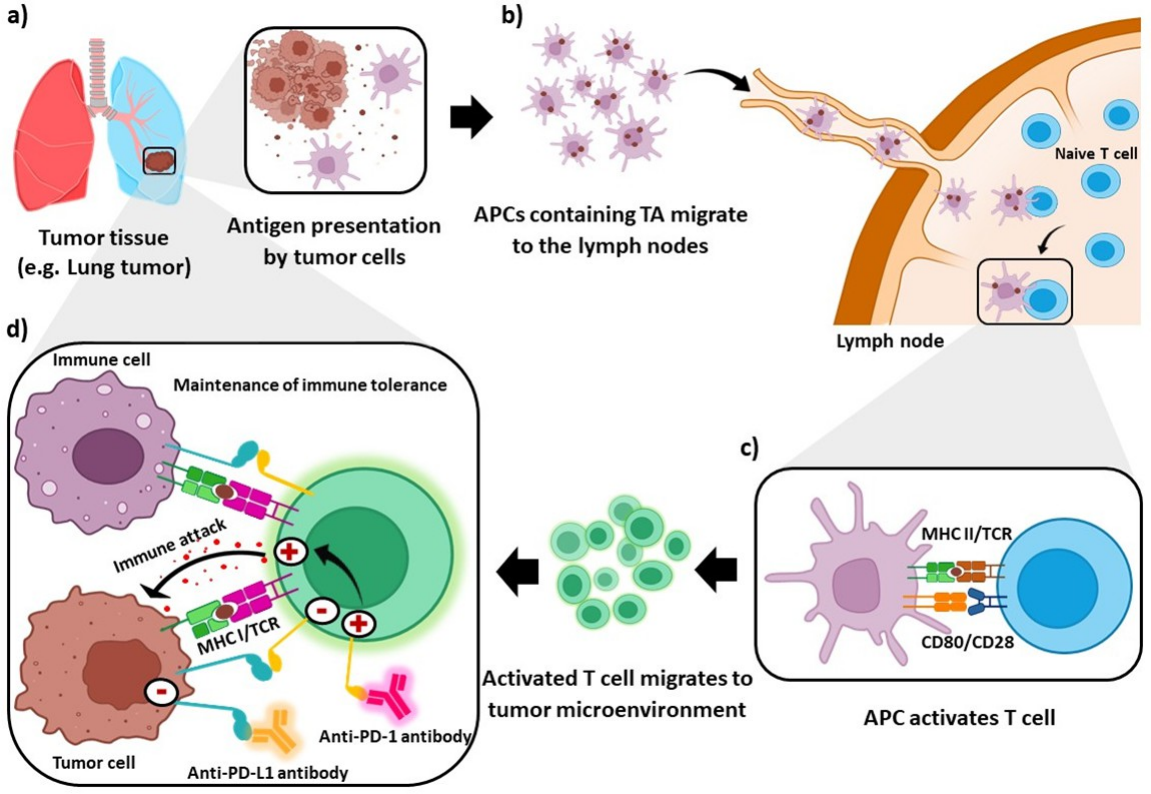
Mechanisms of immune checkpoint inhibitors in cancer immunology. Antigen presenting cells (APCs) process tumor antigens (TA) and present antigens via the major histocompatibility complex (MHC) II to T cell receptors (TCR) on native T cells. Activated T cells migrate to the tumor microenvironment, and PD-1 regulates T cell activation by (1) immune cells and (2) tumor cells, leading to the maintenance of immune tolerance and the effector function of T cells, respectively. The activation of T cells with anti-PD-1 or anti-PD-L1 antibodies leads to the reversal of PD-1 pathway-mediated immunosuppression and tumor cell death.

Dendritic cells (Dcs) patrol peripheral and lymphatic tissues, capture tumor antigens (TA), and present antigens via the major histocompatibility complex (MHC) II for activation of T cells. Activated T cells penetrate the tumor microenvironment, recognize antigens presented by MHC I on cancer cells, and bind them [1]. Immune checkpoints are molecules that normally expressed by immune cells to modulate immune responses in different conditions and are of special usefulness in maintaining self-tolerance to avoid autoimmunity. However, these can be upregulated not only by immune cells but also by tumor cells to control and downregulate the immune responses [2]. Programmed cell death 1 (PD-1) is an immune checkpoint protein that is expressed on the surface of T cells and binds to programmed death-ligand 1 (PD-L1) on the surface of cancer cells [3]. The interaction between tumors and T cells by PD-1/PD-L1 inhibition of the effector function, such as the release of cytotoxins and apoptosis in cancer cells [4]. Cancer cells escape immune system detection and destruction by hijacking the immune checkpoint control mechanisms. Cancer cells highly express PD-L1 ligands and limit productive immune responses of T cells through frequent interactions with cancer cell antigens [2, 5]. In contrast, the interaction between PD-1 and PD-L1 induces T cell apoptosis and inhibits effector functions and T cell proliferation (Fig 1) [6].

**Fig 2:**
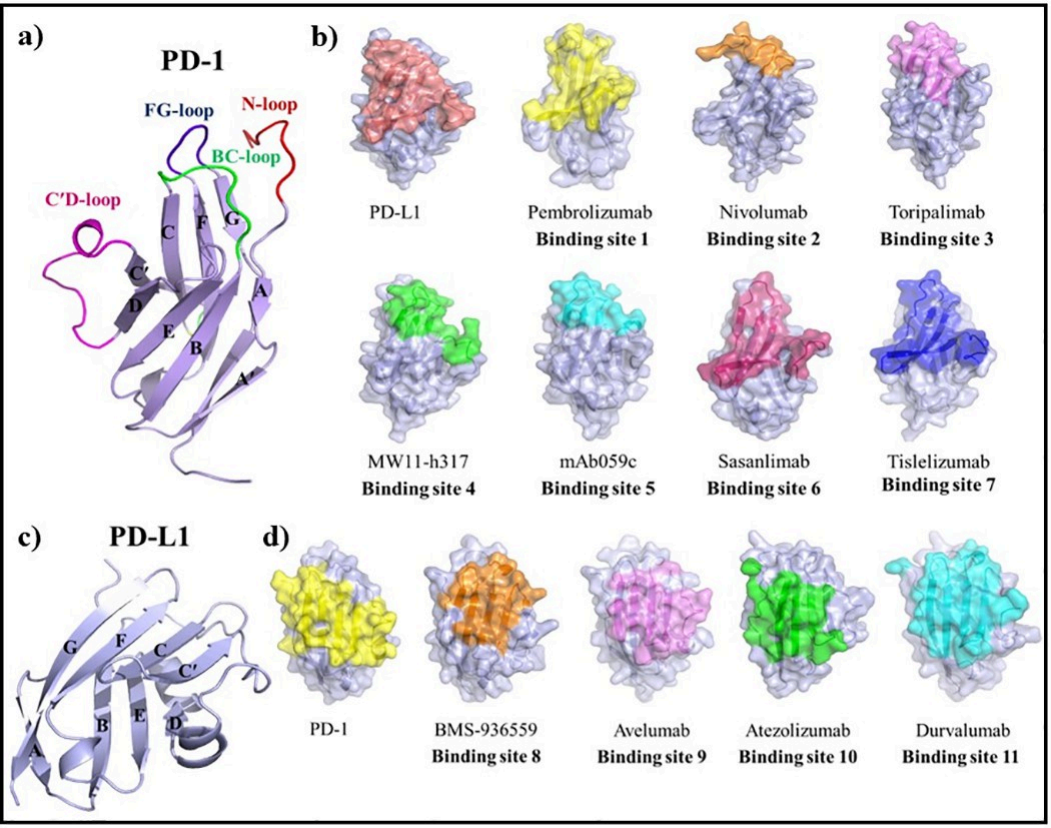
Comparison of the binding surfaces of anti-PD-1 mAbs and anti-PD-L1 mAbs. (a) The structure of the extracellular domain of PD-1 and the BC, C′D, FG, and N loops are colored green, magenta, blue, and red, respectively. (b) Comparison of the binding surfaces of PD-L1 and anti-PD-1 on PD-1 structure. The binding areas of PD-L1 and mAbs targeting PD-1, pembrolizumab, nivolumab, toripalimab, MW11-h317, mAb059c, sasanlimab, and tislelizumab are colored deep salmon, yellow, orange, violet, green, cyan, warm pink, and blue, respectively. (c) The structure of extracellular PD-L1 domain (d) Comparison of the binding surface of PD-1 and anti-PD-L1 on PD-L1 structure. The binding areas of PD-1 and mAbs targeting PD-L1, BMS-936559, avelumab, atezolizumab, and durvalumab are colored yellow, orange, violet, green, and cyan, respectively.

PD-1 is a membrane receptor type I with 288 amino acids and a molecular mass of 31,647 Da. PD-1 in the extracellular region has a β-sandwich topology and is composed of AA′BDCC′FG strands, and N, BC, CC′, C′D and FG loops, an α-helix, and a disulfide bridge (CYS54-CYS123) [3, 7] (Fig 2a). PD-1 binds to the N-terminal domain of PD-L1 via the front β-sheets consisting of the CC′FG strands and FG loop (Fig 2b and 2d) [3, 8]. PD-L1 is a membrane receptor of the B7 family, with 290 amino acids and molecular mass of 33,275 Da. PD-L1 in the extracellular region has two domains consisting of an N-terminal V domain, including two β-sheets, AGFCC′C′′ (front β-sheet) and BED (back β-sheet), and a C-terminal constant domain [3, 8, 9] (Fig 2c).

Protein-protein interactions (PPIs) regulate biological processes in cells, therefore targeting PPIs using inhibitors could influence cell functions. PPIs inhibitors are currently becoming more popular and are recognized as a novel treatment strategy that helps develop new drug generations [10, 11]. The binding of PD-1 to PD-L1 is an aberrant PPI associated with cancer, which is difficult to target therapeutically [12]. The interface area of this interaction is large, hydrophobic, relatively flat, and contains few grooves or pockets. The surface residues on both protein interfaces either continuously or discontinuously bind to the partner side and increase the area of the binding interface, leading to an enhanced binding affinity of the two proteins [11, 13]. Traditional chemical drugs which are known as small-molecule drugs are less effective in serving as PPI inhibitors while antibodies, mini-protein binders, and peptides are shown to be efficient for blocking protein-protein interaction [10, 14, 15]. Monoclonal antibodies (mAbs) are protein-based drugs which their functions and stability could be improved using protein engineering methods, and the market value of this biopharmaceutical has continued to rise at 80.2% of total protein-based biopharmaceutical in 2021 [16]. Also, based on the reports, the global market share for monoclonal antibodies is estimated to be valued at $205.4 billion in 2023 and it expects to rise to $533.6 billion in 2031 [17].

In recent years, blocking immune checkpoints such as PD-1 and PD-L1 with mAbs has become a key solution for the reversal of PD-1 pathway-mediated immunosuppression in cancer immunology [18]. Scientists have found that identifying other immune target agents and combining treatment with other immune checkpoint inhibitors can be effective in achieving a full immune response [19, 20]. Owing to the importance of checkpoint inhibitor therapies, in 2018 Tasuku Honjo and James Allison won the Nobel Prize for the discovery of PD-1 and cytotoxic T-lymphocyte antigen-4 (CTLA-4), respectively [20]. Researchers have also produced mAbs using various laboratory methods, some of them have entered clinical studies that have been successful in the treatment of various tumors and approved by the FDA [18, 21].

In addition, structural studies with a focus on PPIs have provided valuable information to understand the inhibitory mechanisms and binding characteristics of inhibitors, such as mAbs [3, 12, 21]. The current *in silico* methods can accurately accommodate the structural details of PPIs; however, determining favorable binding sites on the protein surface for modulator design of these interactions is a major challenge [22]. Optimizing the binding affinity of antibodies using random mutagenesis techniques in the laboratory has sometimes been successful; however, the process of producing these antibodies and measuring their affinities is not possible without conducting numerous, costly, and time-consuming experiments [23, 24]. *In silico* methods would allow us to study the characteristics of antibodies, build thousands of optimized variants, and save both time and cost for affinity improvement and efficacy of antibodies [25].

In the present study, *in silico* methods used to evaluate and differentiate between 11 different binding sites on PD-1 and PD-L1 to find the most appropriate binding spots on these targets. This research provides accurate and efficient information for further understanding of how designed antibodies interact with these binding sites. Based on the current study, binding sites 1, 3 and 6 form PD-1 and binding sites 9 and 11 form PD-L1 could be more favorable targets for designing mAbs. In addition, thousands of antibodies were generated and optimized for PD-1 and PD-L1, then the designs with higher stability, affinity, and shape complementarity were further subjected to molecular dynamics simulations and MM-PBSA analysis to measure the dynamic, stability, and binding affinity of the final designs. Our results showed that PD-1 has flexible BC, C′D, and FG loops that adopt variable conformations upon binding to antibodies, implying that these loops contribute to the binding affinity for antibodies and provide a hotspots region for immune checkpoint therapy.

## Materials and methods

### Preparation and analysis of antibody-antigen structures

Crystal structures of antibody-antigen complexes were obtained from the Protein Data Bank (PDB). The complex structures included pembrolizumab-PD-1 (PDB ID 5GGS) [26], nivolumab-PD-1 (PDB ID 5WT9) [27], toripalimab-PD-1 (PDB ID 6JBT) [28], MW11-h317-PD-1 (PDB ID 6JJP) [29], mAb059c-PD-1 (PDB ID 6K0Y) [30], sasanlimab-PD-1 (PDB ID 6XKR) [31], tislelizumab-PD-1 (PDB ID 7CGW) [32], BMS-936559-PD-L1 (PDB ID 5GGT) [26], avelumab-PD-L1 (PDB ID 5GRJ) [33], durvalumab-PD-L1 (PDB ID 5XJ4) [34], and atezolizumab-PD-L1 (PDB ID 5XXY) [35]. The constant domain of antibody on both heavy and light chains as well as water, ions, and non-protein residues have been removed using PyMOL 2.3.0 [36]. Python scripts were used to rename the antibodies and target chains as (heavy), L (light), and A (antigen), to ensure compatibility between the designs and simulations. To identify the binding sites, structural analysis of the complexes was performed using PyMOL and COCOMAPS (molnac.unisa.it/BioTools/cocomaps) [37].

### Structural modeling of PD-1

Since PD-1 structure has missing regions in mAbs-PD-1 co-crystal structures, the PD-1 has been modeled using RosettaCM using templates include nivolumab-PD-1 (PDB ID 5WT9), toripalimab-PD-1 (PDB ID 6JBT), MW11-h317-PD-1 (PDB ID 6JJP), and mAb059c-PD-1 (PDB ID 6K0Y). First, PD-1 sequences were aligned and manually converted into a grishin format, then the PD-1 sequence was threaded onto the templates, and disulfide bonds were defined. Rosetta scripts have been run and the top-scoring models have been selected. RosettaCM is a comparative modeling method that assembles homologous structures by recombining aligned segments in Cartesian space and building unaligned torsion-space-based regions and optimizes the all-atom energy function over the defined conformational space [38].

### *In silico* affinity maturation

In this study, the RosettaDesign protocol [39] has been employed to predict changes in the binding affinity of designs upon introducing point mutations on the binding surface of antibody. The crystal structures of the mAbs-PD-1 and mAbs-PD-L1 complexes were obtained from PDB. First, the crystal structures have been relaxed using FastRelaxMover in order to remove clashes between side chains and minimize the backbone tension by adjusting phi and psi torsion angles. Python script was used to define the interface residues, which were written in a file called resfile. Interface residues were defined as heavy atoms of a residue within 5 Å of the heavy atom of a residue on the partner side of the interface. The interface residues, including residues on the antibodies (CDRs sequence), were redesigned, and those on PD-1 and PD-L1 were repacked. RosettaDesign protocol has been employed to redesign the CDRs sequence using a RosettaScript XML file [40]. Each native amino acid was mutated to the other 19 native amino acids, and then backrub motions were applied around the mutation site. In addition, the side chains were simultaneously optimized and the conformation of the backbone coordinates was fixed.

### Analysis of protein-protein interactions and prediction of binding affinity

Rosetta energy function [41] has been employed to calculate the free energy of mutants and maximize the probability of identifying mutations that improve structural stability and antibody-antigen binding affinity. These energy functions include van der Waals packing, hydrogen bonding, and implicit solvation of designs [42]. The optimal design was used to calculate the effects of these mutations on the stability and binding affinity of the interface. A sequence logo was created from our designs via multiple sequence alignments using MultAlin [43] and then used WebLogo representation to show which mutations were made. In addition, the interface characteristics were analyzed using RosettaAnalyzeMover. Using Python scripts, the designs were ranked according to their total score, binding energy, and binding density assigned to the Rosetta energy units (REU).

### Molecular dynamics (MD) simulations

MD simulation technique has been employed in order to understand the structural characteristics of the complexes and the interaction pattern between the antibodies and PD-1 and PD-L1 using AMBER20 [44] with the ff19SB force-field [45]. The systems were neutralized by adding sodium or chloride counterions to the complexes. The systems were then solvated in a truncated octahedral box at 10 Å layer of TIP3P water molecules using xleap [46]. Thereafter, the topology and coordination were saved for the subsequent steps in the simulations. The energy minimization step of the solvated complex was performed in two stages. First, the protein complex was held fixed, the ions and water were minimized by 8000 steps, and then the entire system was minimized by 8,000 steps, including 4000 steps of the steepest-descent and 4000 steps of conjugate gradient algorithms. The SHAKE algorithm [47] was used to constrain the bonds involving hydrogen atoms. Non-covalent interactions were calculated using the Particle Mesh Ewald (PME) algorithm [48] under periodic boundary conditions and with a cutoff distance of 10 Å. The system was heated from 0 to 300 K for 400 ps in a constant-volume ensemble using a Langevin thermostat with NVT [49]. The equilibration step was performed for 5 ns in the NPT ensemble with constant pressure and isotropic position scaling. Finally, production MD simulations were performed for 50 ns using *pmemd*.*cuda* using NPT ensemble, and the coordinates were saved every 4 ps with a time step of 2 fs.

MD simulation has been done for the best 36 designs and 12 control complexes for binding sites 1–11 on PD-1 and PD-L1 receptors.

### Trajectory analysis

To analyze the MD production, *cpptraj* [50] from AmberTools20 was used to calculate the root mean square deviation (RMSD), root mean square fluctuation (RMSF), and number of contacts. Graphs were plotted using gnuplot [51] (http://www.gnuplot.info) and ggplot2 in R [52].

### Analysis of intermolecular interactions of PD-1-PD-L1, antibody-PD-1, and antibody-PD-L1 complexes

The intermolecular interactions of the complexes were analyzed using PyMOL and LigPlot^+^ (v.2.2.5) [53], to assess the interaction of the antibodies with PD-1 and PD-L. Multiple sequence alignments of the design sequences from RosettaDesign were obtained using MultAlin [43]. Network analysis of the complexes was performed using the NAPS [54] (http://bioinf.iiit.ac.in/NAPS/) server for the final structures resulting from the simulation.

### Binding free energy calculation of complexes using MM-PBSA method

To compare the binding affinities of the antibodies for the PD-1 and PD-L1 receptors, the binding free energy (ΔG_binding_) was calculated for 36 of the best-scoring designs and 12 control complexes using the MM-PBSA method [55]. First, the MM-PBSA calculation was performed using Amber 20 for 20 ns with ff99SB force field. Next, an average value of ΔG_binding_ has been calculated from 200 snapshots of trajectories at various time steps by mmpbsa.py [56].

## Results

### Structural modeling of PD-1

Since PD-1 structure has missing regions in mAbs-PD-1 co-crystal structures, including N and C′D loops (PDB ID 4ZQK, 5WT9, and 6GBT), the PD-1 has been modeled using RosettaCM. First, PD-1 sequence was aligned by the query sequences of templates nivolumab-PD-1 (PDB ID 5WT9), toripalimab-PD-1 (PDB ID 6JBT), MW11-h317-PD-1 (PDB ID 6JJP), and mAb059c-PD-1 (PDB ID 6K0Y), manually converted the alignments into a grishin format, threaded the PD-1 sequence onto the templates, and generated a set of partial threads. RosettaCM breaks multiple templates and generates hybridized structures to provide more accuracy. The hybridize mover is defined in Rosetta Scripts and runs the RosettaCM hybridize protocol to generate 1000 comparative designs of the PD-1 protein. Finally, we selected the top-scoring pose as our final comparative design, with an RMSD of 0.455 Å and a score of -1415.767 (Fig 2a).

### Identification of the binding sites of the PD-1-PD-L1, mAbs-PD-1 and mAbs-PD-L1 complexes

Binding sites of PD-1 and PD-L1 were identified with cutoff distance of 8 Å using COCOMAPS and PyMOL for the complex structures included PD-1-PD-L1 (PDB ID 4ZQK) [3], pembrolizumab-PD-1 (PDB ID 5GGS) [26], nivolumab-PD-1 (PDB ID 5WT9) [27], toripalimab-PD-1 (PDB ID 6JBT) [28], MW11-h317-PD-1 (PDB ID 6JJP) [29], mAb059c-PD-1 (PDB ID 6K0Y) [30], sasanlimab-PD-1 (PDB ID 6XKR) [31], tislelizumab-PD-1 (PDB ID 7CGW) [32], BMS-936559-PD-L1 (PDB ID 5GGT) [26], avelumab-PD-L1 (PDB ID 5GRJ) [33], durvalumab-PD-L1 (PDB ID 5XJ4) [34], and atezolizumab-PD-L1 (PDB ID 5XXY) [35] (Fig 2).

PD-1 is a membrane receptor type I, in which the extracellular region has a β-sandwich topology and is composed of β-sheets of CC′FG (front β-sheet) and AA′BDC (back β-sheet), loops, an α-helix, and a disulfide bridge) CYS54-CYS123 ([3] (Fig 2a). PD-L1 is a membrane receptor of the B7 family that has two extracellular Ig domains, an N-terminal V domain including two β-sheets, AGFCC′C′′ (front β-sheet) and BED (back β-sheet), and a C-terminal constant domain [26] (Fig 2c). PD-1 binds to the AGFCC′C′′ β-sheet in the N-terminal domain of PD-L1 via the CC′FG β-sheet and FG loop (Fig 2b and 2d). Fig 2b shows the binding site of antibodies to PD-1; pembrolizumab mainly binds to the C′D and FG loops and CC′ β-sheets, nivolumab binds to the flexible N-terminal region and BC and FG loops, toripalimab binds to the N and FG loops and G strand, MW11-h317 binds to BC, C′D, and FG loops and C′ strand, mAb059c binds to BC, C′D, and FG loops, sasanlimab binds to CC′ and FG loops, and CC′FG β-sheets, and tislelizumab binds to CC′, C′D, and FG loops and CC′FG β-sheets. In addition, BMS-936559 binds to GFCC′ β-sheet, avelumab binds to GFCC′ β-sheet and CC′ loop, atezolizumab binds to GFCC′ β-sheet and CC′ and FG loops, and durvalumab binds to AGFCC′ β-sheet of the IgV domain (Fig 2d). Investigations of the binding mechanism of antibodies to PD-1 and PD-L1 receptors showed that these antibodies bind to the N, BC, CC′, C′D, and FG loops of PD-1 and the AGFCC′C′′ β-sheet of PD-L1, which are key resigns of the binding site of PD-1 to PD-L1.

### Antibody design and analysis

The crystal structures of PD-1-mAbs and PD-L1-mAbs have been prepared including pembrolizumab-PD-1 (PDB ID 5GGS), nivolumab-PD-1 (PDB ID 5WT9), toripalimab-PD-1 (PDB ID 6JBT), MW11-h317-PD-1 (PDB ID 6JJP), mAb059c-PD-1 (PDB ID 6K0Y), sasanlimab-PD-1 (PDB ID 6XKR), tislelizumab-PD-1 (PDB ID 7CGW), BMS-936559-PD-L1 (PDB ID 5GGT), avelumab-PD-L1 (PDB ID 5GRJ), durvalumab-PD-L1 (PDB ID 5XJ4), and atezolizumab-PD-L1 (PDB ID 5XXY). Each of these antibodies binds to a different binding site on the target; therefore, 110000 antibodies were designed for seven different regions on the surface of PD-1 and four different regions on the surface of PD-L1 (each binding site 10,000 designs). Then, using the RosettaDesign protocol, mutations were created in the CDRs sequence of the antibodies. Each native amino acid was mutated to the other 19 native amino acids, then a conformational sampling of the mutated residue’s side chain was performed using rotamer libraries, and backrub motions were applied to the antibody backbone around the binding site to mimic CDRs flexibility in solution and improve sequence diversity. The Rosetta energy function was used to calculate the free energy of the mutated amino acids and identify the amino acids that were minimized. Interface metrics have been calculated for the control and design complexes including total cross-interface hydrogen bonds (hbonds_int), the number of buried unsatisfied hydrogen bonds at the interface (delta_unsathbonds), the solvent accessible area buried at the interface (dSASA_int), the amount of change in the hydrophobic part of dSASA (dSASA-hydrophobic), the change in the polar part of dSASA (dSASA-polar) and other interface metrics for the evaluation of the antibody-antigen interface compared to the interface characteristics of the control and design complexes. In addition, the score of the entire complex or stability (total-score), the binding energy (dG), and the binding density (dG/dSASA) of the designed and control complexes were calculated using the Rosetta scoring function (S1 Table in S1 File). For example, the score of the entire complex (total-score_difference_ = total-score_design_—total-score_control_) of design-1022, design-3753, and design-6013 compared to control-5ggs (pembrolizumab-PD-1) were -3.673, -2.771, and -0.825 REU, with binding energy (dG) differences of -0.113, 0.0049, and -0.9036 REU, respectively. In addition, the dSASA-hydrophobic has increased and the dSASA-polar has decreased, suggesting that the numbers of hydrophobic interactions in the designs were increased.

As shown in S1 Table in S1 File, the highest binding energy (dG) of PD-1-antibody and PD-L1-antibody complexes (< -55 REU) belong to binding sites 1, 6, 7, 9, and 11. The key interacting residues at the 11 binding sites were predicted separately (S4 Table in S1 File). The residues of binding site 1, including Val266, Tyr280, Pro285, Arg288, Ser289, Gln290, Pro291, Ile328, leu330, and Lys333, were key residues involved in antibody-PD-1 interactions.

Next, the candidate designs from a large set of mutated antibodies were screened using Python scripts. According to results, 33.2% of the designed antibodies for PD-1 and 30.6% of the designs for PD-L1 had higher binding affinity (dG), binding density (dG/dSASA) and total-score than those of controls (Table 1).

**Table 1:** The number of the best designs from Rosetta for different binding sites on PD-1 and PD-L1.

Binding site 1	Binding site 2	Binding site 3	Binding site 4	Binding site 5	Binding site 6	Binding site 7	Binding site 8	Binding site 9	Binding site 10	Binding site 11
70	790	352	191	212	44	668	125	707	120	272

**Fig 3:**
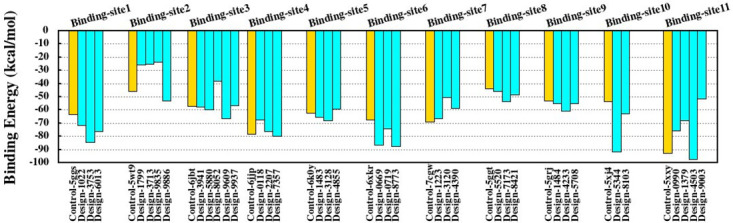
Binding free energy analysis of the control and designed complexes. Binding free energy was analyzed using the MM-PBSA method for all controls (gold) and the designs (cyan) during 20 ns simulation for binding sites 1–11. Control complexes including control-5ggs (pembrolizumab-PD-1), control-5wt9 (nivolumab-PD-1), control-6jbt (toripalimab-PD-1), control-6jjp (MW11-h317-PD-1), control-6k0y (mAb059c-PD-1), control-6xkr (sasanlimab-PD-1), control-7cgw (tislelizumab-PD-1), control-5ggt (BMS-936559-PD-L1), control-5grj (avelumab-PD-L1), control-5xj4 (atezolizumab-PD-L1), and control-5xxy (durvalumab-PD-L1). Fig 2 depicts binding sites 1–11 for binding surfaces of anti-PD-1 mAbs and anti-PD-L1 mAbs.

Then, we selected the best 36 designs from the library which were screened by Rosetta analysis, including 24 designs for 7 different sites on PD-1 (binding sites 1–7) and 12 designs for 4 different sites on PD-L1 (binding sites 8–11). The MM-PBSA technique was used to calculate the binding free energy of the designs from 200 snapshots taken from complexes. MM-PBSA was performed on the control, designs, and PD-1-PD-L1 complexes to assess the effect of mutations on the binding energy. The results of binding free energy analysis of the control and designed complexes are presented in Fig 3.

As shown in Fig 3, highest binding affinities (more negative binding energies) were related to designs 3753, 8773, 5344, and 4503 with -84.5037, -87.8527, -91.9746, and -97.6586 kcal.mol^-1^ for binding sites 1 and 6 on PD-1 and binding sites 10 and 11 on PD-L1. However, analysis of 36 selected designs showed that the binding affinities of these antibodies have increased by 50% and 75% for PD-1 and PD-L1, compared to controls (Fig 3).

Further details of the results from MM-PBSA of designs are presented in S2 Table in S1 File. For example, the results show binding free energies (ΔG_binding_) of designs 1022, 3753, and 6013 in binding site 1, relative to control (pembrolizumab-PD-1), have been improved. The electrostatic energy (EEL) differences (EEL_difference_ = EEL_design_—EEL_control_) of designs 1022, 3753, and 6013 were -17.0086, -37.0799, and -75.931 kcal.mol^-1^, respectively. The van der Waals energy (VDWAALS) differences did not differ significantly. It seems that electrostatic interactions have the main role in binding affinities (ΔGbinding) between designs 1022, 3753, and 6013 and binding site 1 of PD-1.

In addition to the above results, the K_d_ values of the available antibodies against PD-1 and PD-L1 have been extracted from the reports and their binding free energy values have been calculated and compared with the results in the current study (Table 2). The ΔG results showed that binding sites 1, 3, 6, and 7 for PD-1 and binding site 9 for PD-L1 had the highest binding affinity values. Also, comparison of the binding sites revealed that the experimental data were consistent with 4 suggested binding sites from Rosetta results [1, 6, 7, 9] and 2 suggested binding sites from MM-PBSA results [1, 6]. In the next step, the structural characteristics and interactions at the binding sites were investigated to understand the details of each favorable binding site.

**Table 2:** Binding affinity predicted values of mAb-PD-1, mAb-PD-L1, and PD-1-PD-L1 complexes using three methods: Experimental, MM-PBSA, and Rosetta.

Binding site	Protein complex	K_d_ (PM) *	ΔG (kcal.mol^-1^)^‡^	ΔG_binding_ (kcal.mol^-1^)^†^	dG (REU)^⁂^	References
**1**	Pembrolizumab-PD-1	28±1	-14.68±0.32	-63.5046±1.0689	-58.1373	[57–59]
**2**	Nivolumab-PD-1	2.8±0.2 × 10^3^	-11.67±0.04	-45.9166±0.6162	-39.0138	[57, 59]
**3**	Toripalimab-PD-1	23.8 × 10^1^	-13.13	-57.2988±1.2210	-32.4556	[28]
**4**	MW11-h317-PD-1	35.5 × 10^2^	-11.5	-78.3314±0.6077	-47.8669	[29]
**5**	mAb059c-PD-1	40.3 × 10^3^	-10.08	-62.1815±0.6120	-54.92	[30]
**6**	Sasanlimab-PD-1	42±11	-14.17±0.16	-67.5520±0.6288	-67.5875	[31]
**7**	Tislelizumab-PD-1	14±1 × 10^1^	-13.44±0.04	-69.1484±0.9933	-54.758	[60, 61]
**8**	BMS-936559-PD-L1	83 × 10^1^	-12.38	-43.6594±0.6323	-42.1543	[34, 59]
**9**	Avelumab-PD-L1	46.7	-14.09	-52.9111±0.9894	-63.3364	[33, 34, 59]
**10**	Atezolizumab-PD-L1	66.7 × 10^1^	-12.5	-53.3737±0.6736	-39.6586	[34, 59]
**11**	Durvalumab-PD-L1	40 × 10^1^	-12.82	-92.8574±1.2213	-64.6807	[34, 59]
**Native interaction**	PD-1-PD-L1	82±1 × 10^5^	-6.95±0.1	-36.3006±0.6670	-32.6836	[59, 60]

* K_d_: The values of the available mAbs against PD-1 and PD-L1

^‡^ ΔG: The K_d_ value converted to ΔG by ΔG = RTlnK_d_

^†^ ΔG_binding_: Binding free energy calculated using MM-PBSA

^⁂^ dG: Binding energy calculated using Rosetta

### Analysis of RosettaDesign

**Fig 4:**
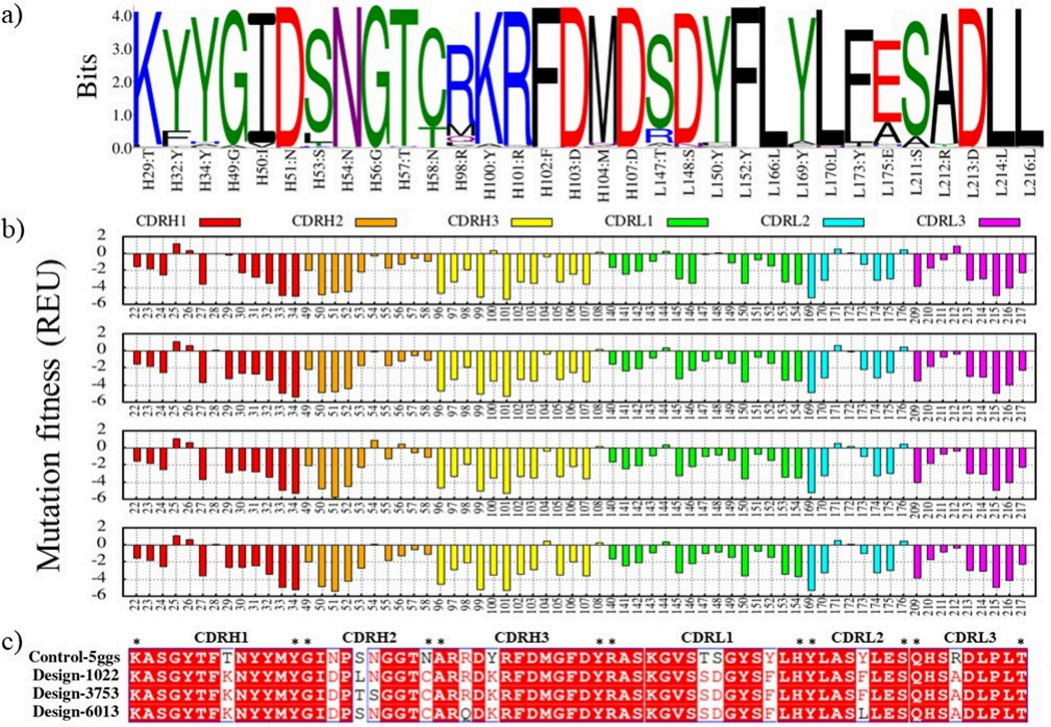
Redesigned CDR sequences for pembrolizumab in complex with PD-1 (control-5ggs). (a) The sequence logo in the 10000 designs. (b) Mutational fitness analysis of each residue in pembrolizumab CDRs sequence. Mutational energy is shown as a function of individual mutations; a more negative energy score is preferred. The CDRH1, CDRH2, CDRH3, CDRL1, CDRL2, and CDRL3 were colored in red, orange, yellow, green, cyan, and magenta, respectively. (c) Alignment of CDR sequences in the control-5ggs and design groups.

The detailed roles of mutations in changing the binding affinity of designs in complexes with receptors have been assessed. A sequence logo was created from our candidate design variants via multiple sequence alignment using WebLogo representation (Fig 4a and 4c). Then the relative contributions of the mutated amino acids to binding affinity have been calculated to determine the impact of each mutation on binding energy (Fig 4b). Comparative analysis showed that mutations play an essential role in antibody-antigen interactions which the binding affinity of antibodies significantly improved. The plots of redesigned CDRs sequence for nivolumab, toripalimab, MW11-h317, mAb059c, sasanlimab, and tislelizumab in complex with PD-1, and BMS-936559, avelumab, durvalumab and atezolizumab in complex with PD-L1 are presented in S1-S10 Figs in S1 File.

Pembrolizumab was selected to describe the results because it has been studied extensively in the treatment of various cancers, such as melanoma, and has been approved by the FDA. As shown in Fig 4b, mutations T29K, Y100K, T147S, S148D, and R212A lead to a decrease in their REU of about -3, -3.1, -1.1, -0.8, and -1.2, respectively (a more negative energy score is preferred); therefore, they may directly affect the binding energy. In addition, mutations N51D, S53L, S53T, and Y173F showed little change in binding energy (> -0.5 REU), whereas other mutations did not change significantly.

According to the results, some mutations had binding energy of < -0.5 REU (a more negative energy score is preferred) and could influence the interactions of antibodies with PD-1 and PD-L1 receptors (Table 3). However, other mutations showed negligible changes, and in some cases had mutation binding energy of > 0 REU. More details of mutation binding energy are shown in S1-S10 Figs in S1 File.

**Table 3:** List of effective mutations in the interactions of the designed antibodies with PD-1 and PD-L1 receptors (binding energy of < -0.5 REU).

Binding sites	Mutations	Design names	Figure
Binding site 1	T29K, Y100K, T147S, S148D, and R212A	1022, 3753, and 6013	3
Binding site 2	T28N, W52S, T173P, and S208K	1799, 3713, and 9835	S1
Binding site 3	H35W, E99V, G100L, T102D, V104E, V104T, V104D, S157P, H223E, and V224F	3941, 5880, 8052, 9609, and 9937	S2
Binding site 4	E146G, V148F, and W166N	0118, 2207, and 7357	S3
Binding site 5	R30E	3128	S4
Binding site 6	T101P and G149S	0669, 0917, and 8773	S5
Binding site 7	N58S, N165A, T172P, T207P, and S210P	1223, 3120, and 4390	S6
Binding site 8	T30E and R214L	5520, 7173, and 8421	S7
Binding site 9	F27N, T28D, T103Y, and R216I	1484, 4233, and 5708	S8
Binding site 10	N50A, R148K, R148P, and Y153H	5344 and 8103	S9
Binding site 11	S30E, S30A, S52V, R99F, P102A, and H210Y	0990, 1379, 4503, and 9003	S10

### Analysis of molecular dynamics simulations

**Fig 5:**
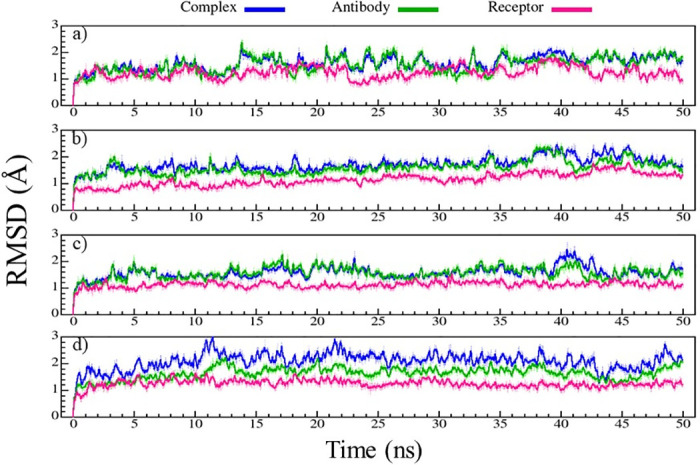
RMSD graphs for complex (blue), antibody (green), and PD-1 (magenta) during 50 ns of MD simulation. (a) control-5ggs (pembrolizumab-PD-1), (b) design-1022, (c) design-3753, (d) design-6013.

To examine the designs more closely and determine the effect of each mutation on structure and stability, MD simulation has been employed. For this purpose, the best 36 designs from the library which were screened by Rosetta analysis, including 24 designs for binding sites 1–7 and 12 designs for binding sites 8–11, as well as 12 control complexes were selected. RMSD of each structure has been obtained from MD simulation during 50 ns and the values have been depicted for complexes, antibodies, and receptors (Fig 5). pembrolizumab-PD-1 (complex), pembrolizumab (antibody), and PD-1 (receptor) had RMSD values 1–2 Å (Fig 5a) and no significant fluctuation was not observed except for a jump around 14 ns of the simulation. The same behavior was observed for designs 1022 and 3753 (Fig 5b and 5c), while design-6013 (Fig 5d) showed higher RMSD values considering other designs. Pembrolizumab mainly binds to the C′D and FG loops which have the highest mobility and flexibility.

As shown in S19a, S21a, and S27a Figs in S1 File, sasanlimab-PD-1 (binding site 6), control-7cgw (binding site 7), and durvalumab-PD-L1 (binding site 10) complexes had RMSD values higher than those of designs. In addition, RMSD fluctuations were more pronounced in design-9835 (S11d Fig in S1 File), design-5880 (S13c Fig in S1 File), design-9937 (S13f Fig in S1 File), and design-1484 (S25b Fig in S1 File) compared to other designs.

**Fig 6:**
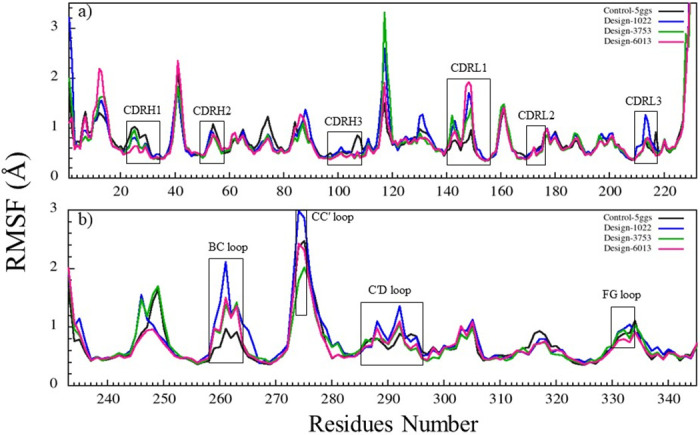
The RMSF graphs of PD-1 and antibodies during 50 ns of MD simulation. RMSF graphs of PD-1 for control-5ggs (pembrolizumab-PD-1, dark gray), design-1022 (blue), design-3753 (green), and design-6013 (magenta). (a) RMSF graphs of antibodies bound to PD-1. (b) RMSF graphs of PD-1 when bound to antibodies.

RMSF analysis was performed to observe the flexibility of PD-1 as well as antibodies in the control and designed complexes (Fig 6 and S11-S30 Figs in S1 File). All best-scoring designs showed decreased flexibility in CDR regions when they were bound to PD-1 (Fig 13). As shown in Fig 6a, the flexibilities of CDRH1, CDRH3, CDRL1, and CDRL3 have changed during the simulation, such that the RMSF values for CDRH1 and CDRH3 in design-1022, design-3753, and design-6013 and CDRL3 in design-3753, design-6013, and CDRL1 in control-5ggs (pembrolizumab-PD-1) were decreased. Fluctuations in PD-1 were more pronounced in the designed complex than in the control complex at residues 244–250, 258–265, and 272–277 and they were not involved in interaction with the antibody. The CC′ loop (274–275) did not interact with antibodies, but as shown in Fig 6, the fluctuations in this loop have changed upon interaction with antibodies in some cases. The residues of the C′D loop (285–296) created a large number of interactions with the CDRs of the antibody, causing displacement of the BC loop (258–264) with significant fluctuation changes. The FG loop (330–334) bound to the antibody during the simulation and the RMSF value did not change significantly. Therefore, despite high sequence and structural similarity between the control and designed structures, the observed differences in local flexibilities arise from the intermolecular interaction patterns in the complex.

In addition, design-9886 (binding site 2, S12 Fig in S1 File), design-5880 and design-9609 (binding site 3, S14 Fig in S1 File), design-7357 (binding site 4, S16 Fig in S1 File), design-3128 and design-4855 (binding site 5, S18 Fig in S1 File), design-8773 and design-0719 (binding site 6, S20 Fig in S1 File), and design-1223 (binding site 7, S22 Fig in S1 File), had significant changes in RMSF values of PD-1 loops (N, BC, CC′, C′D and FG loops) compared to other designs.

RMSD and RMSF graphs for nivolumab, toripalimab, MW11-h317, mAb059c, sasanlimab, and tislelizumab (as control samples) in complex with PD-1 in comparison with the designs, and also BMS-936559, avelumab, durvalumab, and atezolizumab (as control samples) in complex with PD-L1 in comparison with the designs were depicted in S11-S30 Figs in S1 File.

### Contact number analysis during MD simulation

**Fig 7:**
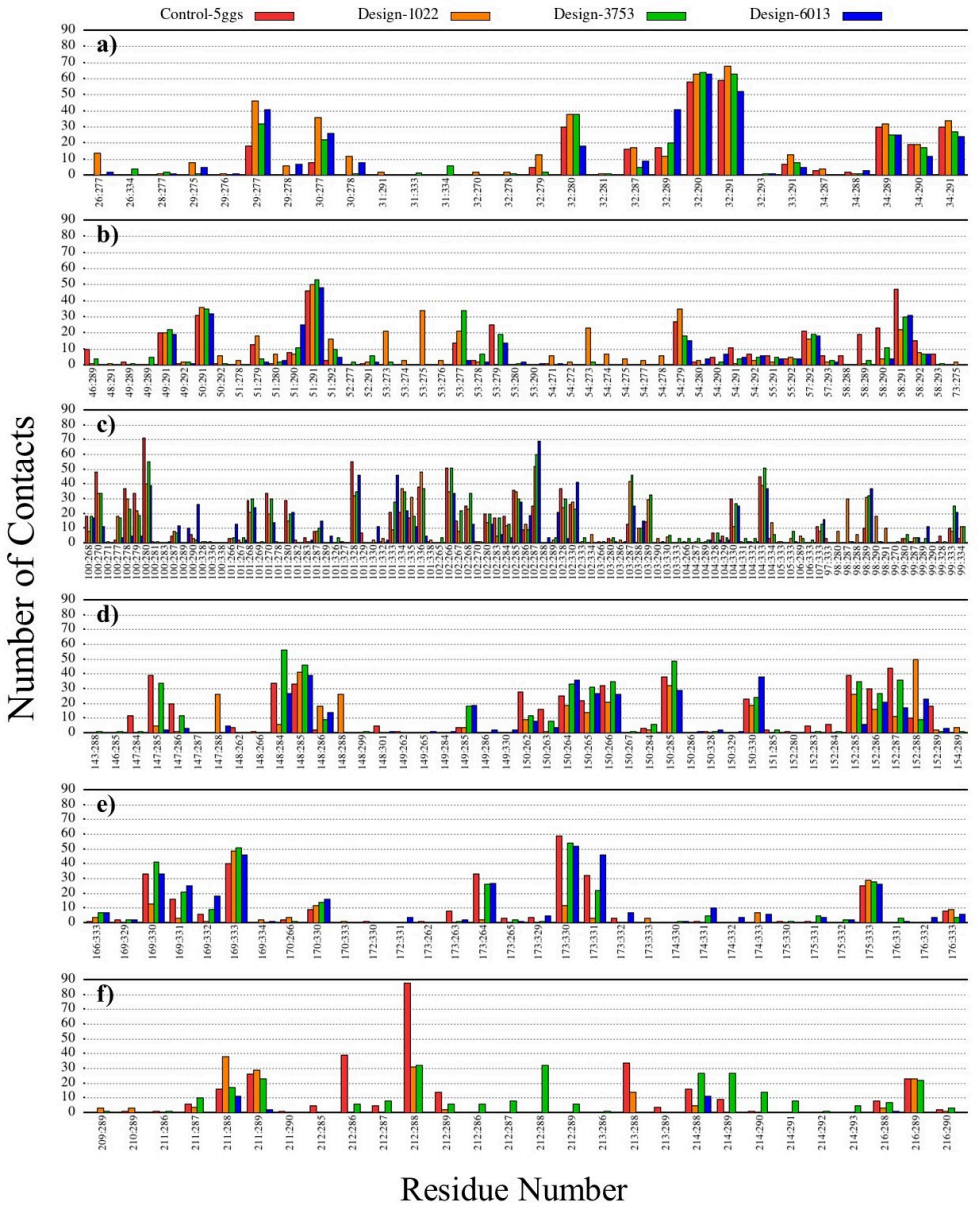
Contact patterns of complexes during 50 ns of MD simulation. Number of contacts for residues in (a) CDRH1, (b) CDRH2, (c) CDRH3, (d) CDRL1, (e) CDRL2, and (f) CDRL3. Control-5ggs-PD-1 (pembrolizumab-PD-1, red), design-1022-PD-1 (orange), design-3753-PD-1 (green), and design-6013-PD-1 (blue). The x-axis shows pairwise residue interactions of the antibody-receptor complex.

Contact numbers have been defined for antibody-antigen complexes using *cpptraj* with a cutoff distance of 8 Å during MD simulations. The contact areas between PD-1 with the CDRs as well as PD-L1 with the CDRs have been extracted by plotting contacts for 50 ns of simulation (Fig 7 and S31-S40 Figs in S1 File). Fig 7 shows the number of contacts in the pembrolizumab-PD-1 (control-5ggs) and design-1022 (T29K, N51D, S53L, N58C, Y100K, T147S, S148D, Y152F, Y173F, and R212A), design-3753 (T29K, N51D, S53T, N54S, N58C, Y100K, T147S, S148D, Y152F, Y173F, and R212A), and design-6013 (T29K, N51D, N58C, R98Q, Y100K, T147S, S148D, Y152F, Y173L, and R212A) complexes for 50 ns of simulation. We compared the contacts between the pembrolizumab-PD-1 and the designs during the simulation and found that mutations not only lead to a change in the contact pattern, but also increase the number of contacts, meanwhile some mutations lead to a decrease in contacts in design-1022, design-3753, and design-6013 compared to the control during the simulation. For example, mutations N51D, T147S, Y152F, Y173F, Y173L, and R212A slightly changed the number of contacts, while mutation N58C led to a decrease in the contacts compared to the control. Mutations T29K, S53T, S53L, R98Q, T147S, and R212A led to changes in the contact pattern and created new contacts. Overall, changes in the number of contacts and patterns compared to the control have been observed. In addition, these changes lead to the creation of new contacts in other residues participating in the interaction between the antibodies and PD-1 (residues N30 and D103 in designs 1022, 3753, and 6013; residues N54 and R98 in designs 1022 and 3753; and residue L214 in design-6013). The main contacts between CDRs of designs and binding sites of the PD-1 and PD-L1 is shown in Table 4. More details of Contact patterns of designs are shown in S31-S40 Figs in S1 File.

**Table 4:** The main contacts between designs and receptors (PD-1 and PD-L1) in binding sites 1–11.

Binding sites	Loops	Strands	CDRs	Figure
Binding site 1	C′D and FG	C and C′	H1, H2, H3, L1, L2, and L3	7
Binding site 2	N, BC, and FG		H1, H2, and H3	S31
Binding site 3	N, C′D, and FG	G	H1, H2, H3, L1, and L3	S32
Binding site 4	BC, C′D, and FG	B, C and C′	H1, H2, H3, L2, and L3	S33
Binding site 5	BC, C′D, and FG	C, F, and G	H1, H2, H3, and L3	S34
Binding site 6	C′D and FG	C, C′, F, and G	H1, H3, L1, and L2	S35
Binding site 7	CC′, C′D and FG	C and C′	H3, L1, and L2	S36
Binding site 8	BC and FG	C, C′, C′′, F, and G	H1, H2, H3, and L3	S37
Binding site 9	CC′	C, C′, C′′, D, F, and G	H1, H2 and H3	S38
Binding site 10	CC′	A, C, C′, F, and G	H2, H3, L1 and L3	S39
Binding site 11	BC, CC′ and FG	C, C′, C′′, F, and G	H1, H2, H3, and L3	S40

### 2D interaction map analysis of PD-1-antibody and PD-L1-antibody complexes

**Fig 8:**
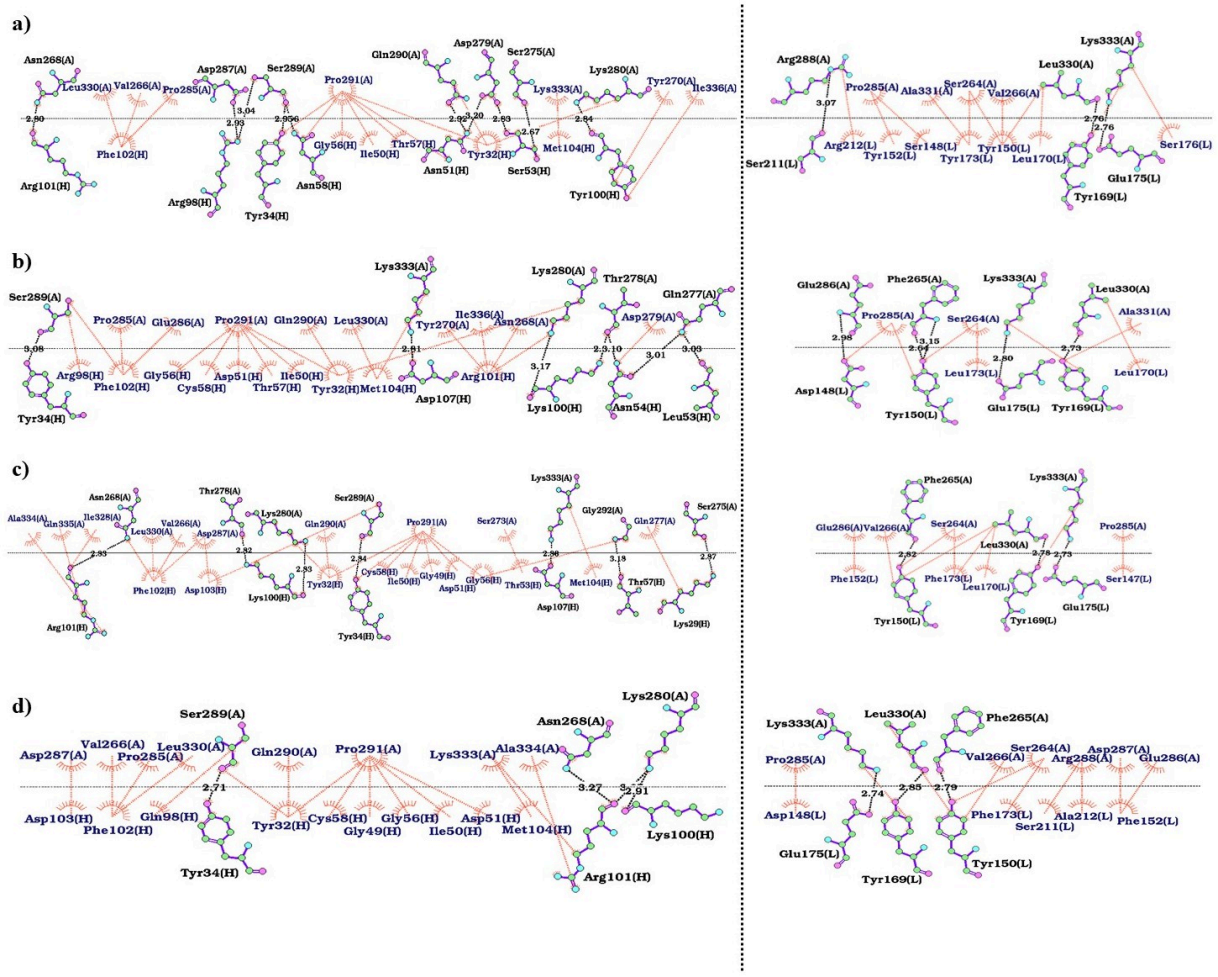
2D interaction maps of heavy (left) and light (right) chains of antibodies in complex with PD-1. The interactions between the light and heavy chains antibody and PD-1 in (a) control-5ggs (pembrolizumab-PD-1), (b) design-1022, (c) design-3753, and (d) design-6013 were analyzed using LigPlot^+^. The hydrogen bonds and hydrophobic interactions are colored in orange and black lines, respectively. The PD-1 and light and heavy chains of the antibody are labeled as A, H, and L, respectively.

Two-dimensional (2D) interaction maps of residue-residue in PD-1-antibody and PD-L1-antibody complexes from the final frames of the simulation were analyzed in order to understand how these residues affect the binding affinity and interactions. The last five frames of the MD simulations have been extracted for 2D interaction analysis. The results showed that mutations in the CDR sequences formed new hydrogen bonds, hydrophobic interactions, and salt bridges between the receptor and the designs during the simulations (Fig 8 and S41-S50 Figs in S1 File). The interactions types between the CDRs of the designs and binding sites on PD-1 [1–7] and PD-L1 [8–11] are summarized in S3 Table in S1 File.

The control-5ggs (pembrolizumab-PD-1), design-1022, design-3753, and design-6013 complexes were studied and showed that the number of hydrophobic interactions increased and the number of hydrogen bonds decreased (Fig 8), which consistent with increased binding affinity of the designs (Fig 3, S2 and S3 Tables in S1 File). Mutations T29K, S53L, S53T, Y152F, Y173F, and Y173L increased the binding affinity of the designs by forming new hydrogen bonds and hydrophobic interactions compared to the control.

### Binding free-energy decomposition

**Fig 9:**
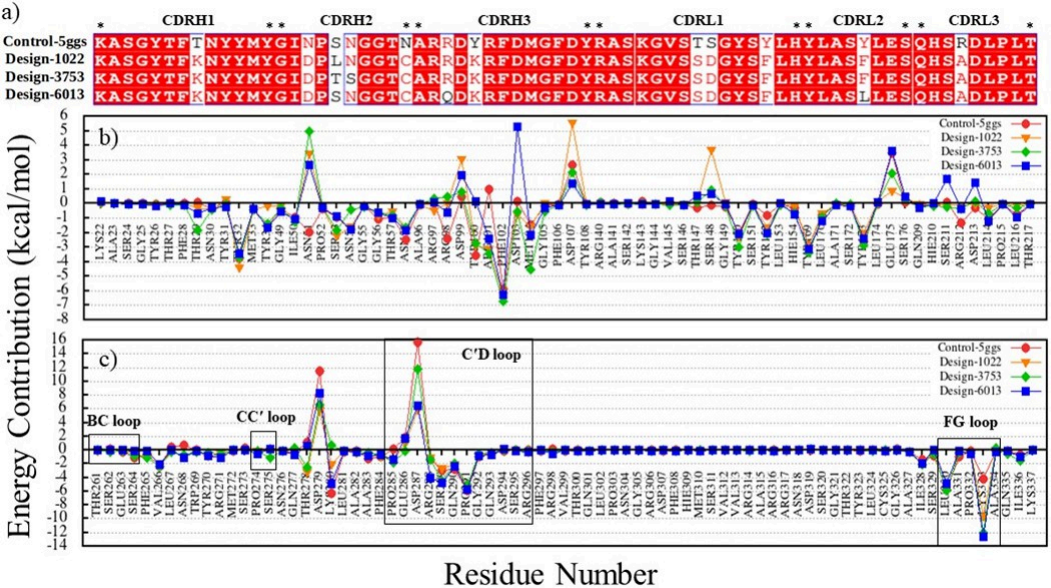
Binding free energy decomposition of the residues in the control and designed complexes. (a) Alignment of CDR sequences in the control-5ggs (pembrolizumab-PD-1) and design groups. (b) Energy contribution values of PD-1 residues from MM-PBSA. (c) Energy contribution values of CDR residues from MM-PBSA. The graphs show the binding free energy for each residue in control-5ggs (red), design-1022 (orange), design-3753 (green), and design-6013 (blue).

Analysis of the free energy decomposition of the residues in the control and designed complexes on CDRs and receptors (PD-1 and PD-L1) has been performed. This analysis could improve our insight in understanding the contribution of every single residue in interaction and its role in binding energy changes. Fig 9 shows the binding free energy decomposition of residues in the control-5ggs (pembrolizumab-PD-1), design-1022, design-3753, and design-6013 complexes.

Comparison of the decomposition results with the control will be useful to determine the binding free energy differences for mutations and residues on the binding interface (ΔG_difference_ = ΔG_binding-design_ − ΔG_binding-control_). According to the data presented in S5 Table in S1 File and Fig 9, mutations T29K, S53L and Y152F and residues Y32, R97, R101, D103, Y150, E175 in design-1022; mutations T29K, S53T, Y152F, and Y173F and residues R101, F102, D103, M104, G105, D107, Y150, and E175 in design-3753; mutations T29K, Y152F, and Y173L and residues M104 and D107 in design-6013 (Fig 9b) have main role in interaction of antibodies with PD-1. However, the binding free energy differences of the other mutations and residues were not significant. These significant differences in the binding free energy could be related to changes in the interaction patterns (Fig 7), formation of new contacts (Fig 8) as well as increased electrostatic share (S2 Table in S1 File) in the binding interface. In addition, residues N268, T278, D279, P285, E286, D287, R288, S289, and K333, which are located in C and C′ strands (265–273 and 276–284), and C′D (285–296) and FG (230–234) loops from the PD-1 side had the main role in interaction with antibodies, (Figs 7 and 9c).

**Fig 10:**
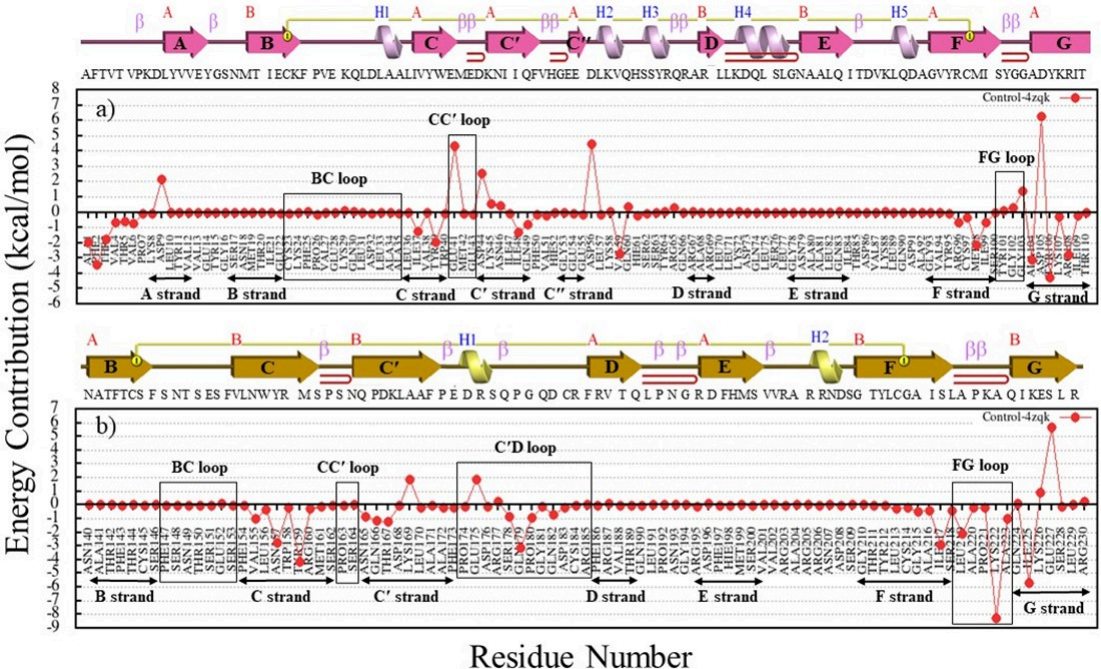
Binding free energy decomposition of the residues in the PD-1-PD-L1 complex. (a) Energy contribution values of PD-L1 from MM-PBSA calculations for residues in strands A (residues 8–12), C (residues 35–40), C′ (residues 44–50), F (residues 91–99), G (residues 103–112) of PD-L1. (b) Energy contribution values of PD-1 residues from MM-PBSA.

Next, the interaction mechanism of PD-1 with PD-L1 has been investigated and results are presented in Fig 10 and S2 Table in S1 File. The roles of surface residues have been also assessed; it seems that residues N157, Y159 (C strand), Q179 (C′D loop), I217 (F strand), L219, K222 (FG loop), and I225 (G strand) with binding free energies of -2.8, -4.2, -3.1, -2.9, -2.1, -8.1, and -5.7 kcal.mol^-1^ play a major role in the interaction of PD-1 with PD-L1. Also, from the PD-L1 side, residues A1, F2, T3, I37, Y39, I48, V59, M98, A104, Y106, and N108 (C, C′, F, and G strands) with binding free energies of -2, -3.4, -1.9, -1.2, -2.1, -1.3, -2.9, -2.2, -3.2, -4.1, and -1.9 kcal.mol^-1^ had the main role in interaction with PD-1.

### Investigation of conformational changes of loops of PD-1 in complex with antibody

**Fig 11:**
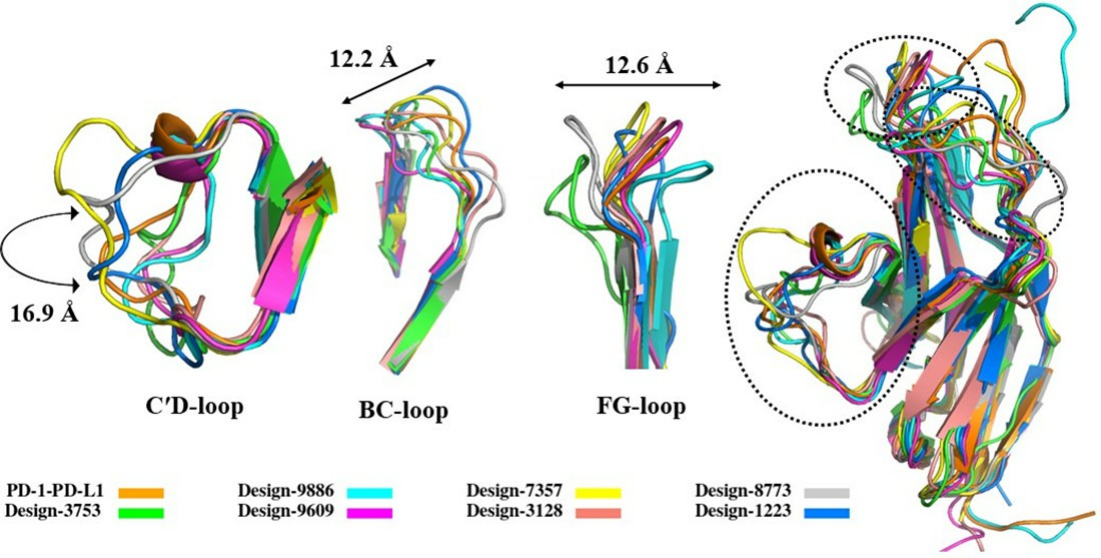
Comparison of conformational changes in the PD-1 loops in complex with antibodies. The PD-1 loops from different complex structures including PD-1-PD-L1 (PDB code:4ZQK, orange), Design-3753 (green), Design-9886 (cyan), Design-9609 (light magenta), Design-7357 (yellow), Design-3128 (salmon), Design-8773 (gray), and Design-1223 (marine). The FG, BC, C′D loops of PD-1 shifted up to 12.6, 12.2 and 16.9 Å, respectively, upon binding to different antibodies.

**Fig 12:**
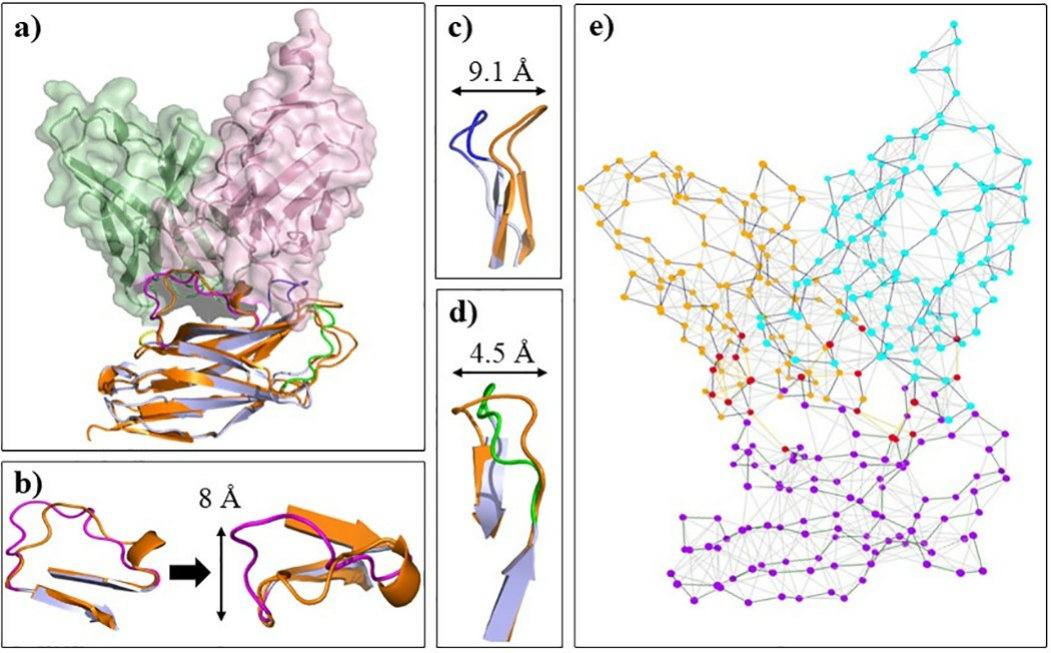
Analysis of conformational changes within PD-1 loops in complex with design-3753. (a) Comparison of conformational changes in the PD-1 loops upon binding to design-3753 and PD-L1. The heavy chain, light chain, PD-1 in complex with design-3753, and PD-1 in complex with PD-L1 are colored pale green, light pink, light blue, and orange, respectively. The C′D, FG, and BC loops of PD-1 in complex with design-3753 are colored in magenta, blue, and green, respectively. (b) The C′D loop of PD-1 shifted 8 Å in the groove which was formed by the CDR loops of design-3753. (c) The FG loop of PD-1 shifted 9.1 Å upon binding to design-3753. (d) The BC loop of PD-1 shifted 9.1 Å upon binding to design-3753. (e) Interaction network between PD-1 loops and the design-3753. The amino acids in the heavy chain, light chain and PD-1 are colored as cyan, gold, and violet nodes, respectively. Red nodes represent the amino acids which are participate in PD-1 interaction. Analysis of loop conformational changes for other designs have been depicted in S61-S66 Figs in S1 File.

In the next step, the role of loop flexibility in the binding affinity and formation of the antibody-PD-1 complex has been investigated. Therefore, conformational changes of the FG, BC, and C′D loops of PD-1 upon interaction to antibodies in 7 different regions (binding site 1–7) during simulation have been studied. At first, the antibody-PD-1 and PD-1-PD-L1 complex structures have been extracted from several frames of the MD simulations and the structures have been aligned (Fig 11). Then, the displacements of N, BC, C′D, and FG loops upon binding to antibodies were measured. As shown in Fig 11, the FG, BC, C′D loops of PD-1 shifted up to 12.6, 12.2 and 16.9 Å, respectively, upon binding to different antibodies. Based on the results, the FG loop adopts mainly varied conformations upon binding to the designed antibodies or PD-L1. Also, conformational change in the BC loop is depend on the binding partner, and in some designs the C′D loop intrudes into the groove formed by the CDR loops, leading to a stronger binding of PD-1 to antibodies (Figs 11 and 12 and S61-S66 Figs in S1 File).

The FG loop of PD-1 had high mobility upon binding to the designed antibodies and has shifted around 9.1 Å in design-3753 (Fig 12c), 3.5 Å in design-9886 (S61c Fig in S1 File), 0.4 Å in design-9609 (S62c Fig in S1 File), 5.4 Å in design-7357 (S63c Fig in S1 File), 1.6 Å in design-3128 (S64c Fig in S1 File), 8.2 Å in design-8773 (S65c Fig in S1 File), and 6.1 Å in design-1223 (S66d Fig in S1 File) compared to its position when binds to PD-L1. In similar manner, the BC loop has shifted 4.5 Å in design-3753 (Fig 12d), 3.6 Å in design-9886 (S61d Fig in S1 File), 7.1 Å in design-9609 (S62d Fig in S1 File), 3.1 Å in design-7357 (S63d Fig in S1 File), 4.5 Å in design-3128 (S64d Fig in S1 File), 4.5 Å in design-8773 (S65d Fig in S1 File), and 5.1 Å in design-1223 (S66e Fig in S1 File). The C′D loop had conformational shift of 8 Å (Fig 12b), 8.9 Å (S63b Fig in S1 File), and 8.5 Å (S65b Fig in S1 File) when entered to the groove which were formed by the CDR loops of the design-3753, design-7357 and design-8773, respectively (Fig 11). Conformational changes of PD-1 loops upon binding to designs 3753, 9886, 9609, 7357, 3128, 8773, and 1223 compared to these loops when binds to PD-L1 are presented in S1 Video (design-3753), S2 Video (design-9886), S3 Video (design-9609), S4 Video (design-7357), S5 Video (design-3128), S6 Video (design-8773), and S7 Video (design-1223). The heavy chain, light chain, PD-1 in complex with designs, and PD-1 in complex with PD-L1 are colored pale green, light pink, light blue, and orange, respectively, as well as the N, C′D, FG, CC′, and BC are colored in red, magenta, blue, green, and yellow, respectively.

Next, the contributions of the binding energies of the N, BC, C′D, and FG loops in PD-1-PD-L1 and the best-designed complexes were calculated. As shown in Table 5, the binding energies of the loops were significant, indicating that they played an important role in increasing the binding affinities of the designs. According to the binding energies of FG loop in the designed and PD-L1 complexes, we found that the FG loop is a hotspot site on PD-1 for the designed antibodies.

**Table 5:** The binding free energies (kcal·mol^−^1) for the N, BC, CD, FG, and CC′ loops of PD-1 in complex with best designs calculated by MM-PBSA. The binding free energies for the N, BC, C′D, FG, and CC′ loops are the sum of the binding free energy of the individual residues within the loop structures. Values of ±0.01 were considered approximately 0 kcal.mol^-1^.

	PD-1-PD-L1	Design-3753	Design-9886	Design-9609	Design-7357	Design-3128	Design-8773	Design-1223
**N-Loop**	18.2	0	-1.73	-1.2	0	0	0	0
**BC- Loop**	-0.1	-0.07	-3.86	4.15	-2.3	6.3	0	0
**C′D- Loop**	-4.1	-4.3	-0.34	-3.9	-11.4	-19.9	-7.8	-12.5
**FG- Loop**	-11.8	-19.1	-16.45	-21.7	-8.5	-7	-8.3	-3.58
**CC′- Loop**	-0.05	-1.2	0	0	0	0	-0.08	-0.34

**Fig 13:**
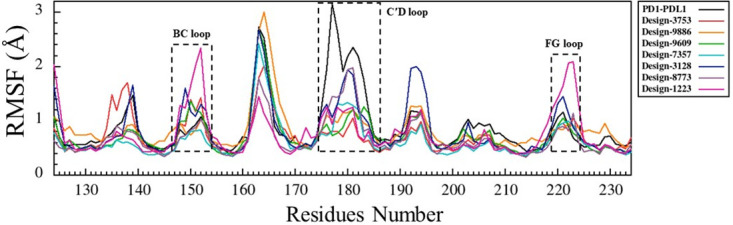
Loop flexibilities in PD-1 upon binding to the PD-L1 and designs during simulation. The RMSF of Cα of PD-1 when bound to PD-L1 (black), design-3753 (red), design-9886 (orange), design-9609 (green), design-7357 (cyan), design-3128 (blue), design-8773 (violet), and design-1223 (deep pink).

Finally, flexibility analysis of BC, C′D, and FG loop of PD-1 upon binding to PD-L1 and the designs during simulation have been assessed (Fig 13). Investigation of the N, BC, CD, and FG loop flexibilities and their binding energies (Table 5) upon binding to the designs and PD-L1 revealed that with increased in binding affinity (a more negative binding free energy), the flexibility of the loops decreased. Also, the interaction networks analysis for the designs and PD-1-PD-L1 complexes showed that in design-3753 and control-4zqk (PD-1-PD-L1), the BC loop did not directly involve in the interaction, while binding the C′D loop to the antibody led to a conformational change in the BC loop (Fig 12d and 12e). Design-9886 did not directly interact with the C′D loop, and also the PD-L1 has a minor interaction with the C′D loop, but its fluctuation was increased in PD-1-PD-L1 complex (control-4zqk) compared to design-9886 (Fig 13).

Furthermore, the binding of PD-L1 to PD-1 led to CC′ loop rearrangement in the form of a 90° twist and changed from the “open” to the “closed” conformation [3, 62, 63]. However, the CDRs of design-1223 (binding site 7) uniquely bind to the CC’ loop of PD-1 (S66 Fig in S1 File) and prevent the change of the conformation towards the “closed” and block the binding site of PD-L1.

In addition, we found a rearrangement in the side chains of residues involved in the interaction of PD-L1 with PD-1, and the designed antibodies did not significantly induce conformational changes in the PD-L1.

## Discussion

Immune checkpoints play a key role in the dynamic and balance of the immune system and modulate immune responses in tumor tissues [2, 64]. Cancer cells can avoid immune surveillance by targeting immune checkpoints via expressing their inhibitory ligands such as PD-1/PD-L1 axis [18]. Many of cancer types use the PD-1/PD-L1 pathway, which is associated with inhibitory PD-1 function and PD-L1 overexpression on cancer cells to deactivate T cells [65, 66]. In recent years, cancer treatment using antibodies to block inhibitory immune checkpoints has enabled the development of biotherapeutics that led to an increase in the market value of biopharmaceuticals which has continued to rise [16, 19].

In this study, we describe an in depth and exhaustive investigation on the binding sites of PD-1 and PD-L1 to find the most appropriate binding sites for antibody optimization. Then thousands of variants have been generated and assessed on a large scale to enhance their efficacy for immune check point blockade against PD-1 and PD-L1. We compared the binding energy values of the mAb-PD-1, mAb-PD-L1, and PD-1-PD-L1 complexes using three methods: experimental data, MM-PBSA, and Rosetta. According to the experimental data (ΔG) presented in Table 2, binding sites 1, 3, 6, and 7 for PD-1 and binding site 9 for PD-L1 had the highest binding affinities. Comparison of the binding sites showed that the experimental data were consistent with the outcomes of Rosetta for binding sites 1, 6, 7, and 9 and the outcomes of MM-PBSA for binding sites 1 and 6. We then focused on the key interacting residues at the 11 binding sites to understand their roles in PPIs (S4 Table in S1 File). However, the current experimental methods cannot accommodate accurate structural details of PPIs and the dynamics of the structures to differentiate between 11 different binding sites on PD-1 and PD-L1.

First, Rosetta antibody design protocol was employed to introduce mutations in CDR sequences. The binding energies of the mutations have been calculated and changes in the binding affinity upon mutations have been quantitatively predicted. As per the data presented in Table 3 from the Rosetta mutation binding energy results, some mutations had significant changes and could directly affect the interactions of antibodies with PD-1 and PD-L1. However, other mutations did not show significant changes, and some mutations had a negative effect. It seems that the activity of all or most mutations contribute to specific receptor-antibody interactions and increased antibody binding affinity, which means that some mutations may contribute in the recognition process, whereas others directly involved in the interaction and improve the binding affinity of the antibody. Based on the results, the most successful design rates and the highest binding improvement of the designs were related to the binding sites 1, 6, and 7 on PD-1, and 9 and 11 on PD-L1.

In the next step, MD simulation has been employed to investigate the dynamic and flexibility of the structures, and describe the influence of mutations on the interaction of designs with targets (PD-1 and PD-L1). To understand the roles of the loops in interaction with designed antibodies, the RMSD graphs for PD-1 with and without BC, C′D and FG loops have been calculated (S67-S73 Figs in S1 File). Analysis of conformational changes of PD-1 loops when bound to the antibodies (S67-S73 Figs in S1 File) as well as contact analysis of the binding interface (Fig 7 and S31-S40 Figs in S1 File), showed that the conformational changes of the BC, FG, C′D, and CC′ loops in PD-1 significantly contribute to the binding process of PD-1 to designed antibodies. Consistent with our results, Mittal *et al*. showed that the flexibility of PD-1 loops (N, BC, FG, C′D, and CC′ loops) are responsible for the molecular recognition and binding process to mAbs [63]. As shown in S67 Fig in S1 File, the FG loop had an RMSD value of 0.2 Å in all designs and control-5ggs (pembrolizumab-PD-1), and no significant fluctuation was observed. However, the C′D loop in design-6013 and the BC loop in designs 1022 and 6013 had considerable RMSD fluctuations. Also, the RMSD values of PD-1 with and without C′D and BC loops had a significant difference when bound to design-6013.

Contact pattern analysis was used to understand more details from the protein–protein interaction of control and designed complexes during the simulation. Based on the contact pattern results, the pattern and number of contacts for CDRs of designs in complex with their targets (PD-1 and PD-L1) had changed compared to those of controls. Mutations with positive changes in binding energy (≤ -1 REU) mostly increased the number of contacts (more negative energy is preferred). Our results showed that design-3753 (binding site1, Fig 7); design-9886 (binding site2, S31 Fig in S1 File); design-9609 (binding site 3, S32 Fig in S1 File); design-7357 (binding site 4, S33 Fig in S1 File); design-3128 (binding site 5, S34 Fig in S1 File); design-8773 (binding site 6, S35 Fig in S1 File); control-7cgw (binding site 7, S36 Fig in S1 File); design-8421 (binding site 8, S37 Fig in S1 File); design-4233 (binding site 9, S38 Fig in S1 File); design-5344 (binding site 10, S39 Fig in S1 File); design-4503 (binding site 11, S40 Fig in S1 File) had significant changes in the pattern and number of contacts, it seems that these mutations play a fundamental role in the interaction of antibodies with PD-1 and PD-L1 receptors.

Two-dimensional (2D) interaction maps of residue-residue in PD-1-antibody and PD-L1-antibody complexes from the last five frames of the MD simulation were analyzed. According to the results, some mutations in the designs enhanced the binding affinity by the formation of new interactions between targets and their designs including new hydrogen bonds in design-3128 (binding site 5, S44c Fig in S1 File), design-4233 (binding site 9, S48c Fig in S1 File), design-5344 (binding site 10, S49b Fig in S1 File), and design-4503 (binding site 11, S50d Fig in S1 File), new hydrophobic interactions in design-3753 (binding site 1, Fig 8c), design-9609 (binding site 3, S42f Fig in S1 File), design-7357 (binding site 4, S43d Fig in S1 File), design-8773 (binding site 6, S45d Fig in S1 File), design-7173 (binding site 8, S47c Fig in S1 File), and design-4233 (binding site 9, S48c Fig in S1 File), and new salt bridges design-9886 (binding site 2, S41e Fig in S1 File) and design-5344 (binding site 10, S49b Fig in S1 File), whereas some other mutations did not increase the affinity (S46b-S46d Fig in S1 File).

In the last step, the binding free energies of the designs were calculated using the MM-PBSA method, in which binding sites 1 and 3 on PD-1 and binding site 10 and 11 on PD-L1 had the highest binding affinities (Fig 3). The MM-PBSA analysis was also in accordance with the results from interaction pattern and contact numbers analyses, and showed that mutations in the residues at the CDR interface area improved the affinity of the designs as well as their structural stabilities. According to some reports, a series of point mutations within the sequences of the variable regions of heavy (V_H_) and light (V_L_) chains of antibodies increases the binding affinity [24, 25, 67–71]. Ye *et al*. optimized the binding affinity of antibodies 42A1 (approximately 2.6 fold) and I4A3 (approximately 3.7 fold) to target the liver cancer antigen glypican-3 and severe acute respiratory syndrome coronavirus 2, respectively, through single mutations in the CDRs [24]. Lippow *et al*. obtained 10 fold and 140 fold affinity improvements for cetuximab (anti-EGFR antibody) and D44.1 (anti-lysozyme antibody), respectively, by combining multiple designed single mutations in all CDRs [70]. On the other hand, some studies reported that mutations in the CDRs interface can improve the structural stability of CDRs as well as the binding affinity of antibody [23, 68, 72]. Warszawski *et al*. showed that mutations in the variable fragment (FV) interface of anti-lysozyme antibody simultaneously enhanced affinity and improved stability by rearrangement of the antigen-binding site and eliminating packing defects of mutations during binding, respectively [23]. Acierno *et al*. determined that mutations in the antibody F10.6.6 correlate with increased binding affinity, and the conformational changes at the V_H_-V_L_ interface improved FV domain stability [72].

For more details, the free energy decomposition analysis of residues at the interface area of the CDRs has been performed. According to our results, the high affinity of the designs can be attributed to the concerted effect of all or most single-point mutations in the CDRs. However, these mutations lead to improved structural stability of CDRs by correction in the packing of the CDRs interface. Adams *et al*. measured the dissociation constant of antibodies and performed the yeast display assay to define their binding free energies. Then, they described epistasis by the binding free energy and suggested that the binding affinity increases in the presence of multiple mutations and intramolecular epistasis that revolved around the evolution of antibodies [73]. For instance, mutations N51D, T147S, and S148D in design-3753 have unfavorable binding free energy contribution (Fig 9b) which seems to be deleterious by themselves, and mutations T29K, S53T, N54S, N58C, Y100K, Y152F, Y173F, and R212A have favorable binding affinity share, however, the total set of mutations at the CDRs region resulted in increased binding affinity (-84.5037 kcal.mol^-1^) compared to the control-5ggs (pembrolizumab-PD-1, -63.5046 kcal.mol^-1^). The conformational dynamic of residues at the protein-protein interface are dependent on physical interactions between residues [74]. According to some studies, multiple mutations with changes in intramolecular interactions lead to specific conformational motions which enhances or disrupts protein function [74, 75]. We suggest that point mutations in the CDRs sequences could change CDRs dynamic, improve binding affinity and structural stability.

In addition, we compared ΔG_binding_ from MM-PBSA with the dG calculated by the RosettaDesign (S1 and S2 Tables in S1 File). According to the results, in 60% of cases, binding affinity trends from Rosetta were in accordance with the MM-PBSA calculation but some designs showed significant differences in the results. For example, in case of design-1799, Rosetta calculation showed higher binding affinity compared to control-5wt9 (nivolumab-PD-1) (-45.1498 and -39.0138 REU, respectively), while the affinity of this design is lower, according to the MM-PSA results (-28.9920 and -45.9166 kcal.mol^-1^ for design-1799 and control-5wt9, respectively). This difference in the calculation of binding energy could be attributed to approximations made in the Rosetta energy function and MM-PBSA method. The Rosetta Energy Function 2015 (REF15) for solvation modeling uses the solvent as bulk water based on the Lazaridis-Karplus (LK) implicit Gaussian exclusion model including isotropic solvation energy (fa_sol) for protein atoms in different residues and anisotropic solvation energy (lk_ball_wtd) for polar atoms [34, 63]. While the MM-PBSA solvation model was an explicit solvent [45] and the TIP3P water model was used to solvate the system. Additionally, MM-PBSA calculates binding free energy from hundreds of frames during simulation in which the complex may have different conformational states, while Rosetta energy function approximation does not consider such huge conformational sampling space compared to MD simulation.

**Fig 14:**
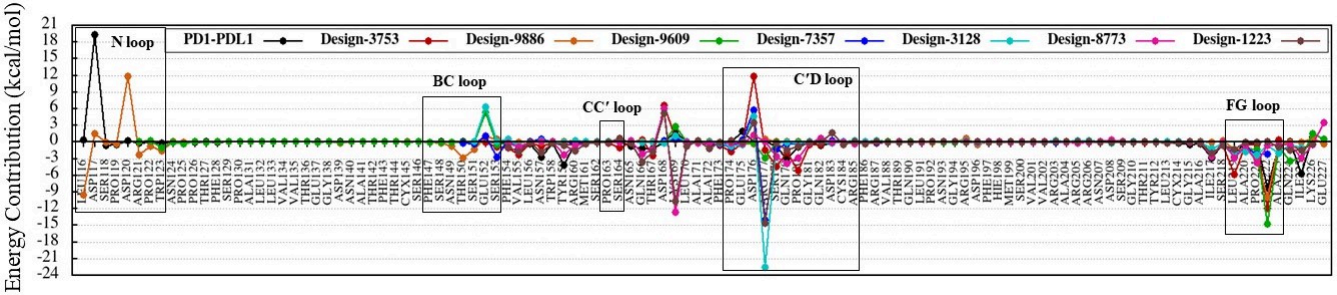
Comparison of binding free energy decomposition of the loops of the PD-1 in complex with designs. The graphs show the binding free energy changes for each residue in PD-1-PD-L1 (black), Design-3753 (red), Design-9886 (orange), Design-9609 (green), Design-7357 (cyan), Design-3128 (blue), Design-8773 (violet), and Design-1223 (magenta).

According to the above results, the structural plasticity of the FG, BC, and C′D of PD-1 loops upon interaction with antibodies has been investigated during simulation. The FG, BC, and C′D loops in the designed and PD-1-PD-L1 complexes had high mobility, and structurally shifted up to 12.6, 12.2, and 16.9 Å, respectively (Fig 11). Although the FG and C′D loops adopt mainly varied conformations upon binding to the designed antibodies, it plays a critical role in the interactions with the designed antibodies and PD-L1. The binding free energies in the residues of FG and C′D loops changed during interactions with the antibodies and PD-L1 (Figs 9, 10 and 14 and S51-S60 Figs in S1 File) and the contributions of the binding free energies of the FG and C′D loops upon binding to antibodies and PD-L1 (Table 5) showed that they could be considered as hotspot on PD-1. Therefore, this varied conformation directly helps the FG loop to bind to the designed antibodies and can be the main druggable target for protein-based drug, such as mAbs [69]. Previous studies showed that the FG loop is a hotspot for mAbs [3, 76, 77], which is in line with our findings. Chen *et al*. reported antibodies GY-5 and GY-14, for PD-1-PD-L1 blocking that FG loop of PD-1 was targeted in the interaction with both mAbs. They performed X-ray crystallography to investigate the conformational variations of the FG loop upon binding to different mAbs and found that the FG loop of PD-1 is a hotspot for mAbs [77]. Consistent with our results, Roither *et al*. [78] indicated that the BC loop is flexible, and conformational changes in the BC loop depend on the binding partner. Tan *et al*. used crystallography experiments to show that when nivolumab binds to PD-1, the BC-loop is shifted at approximately 5.3 Å compared when the PD-L1 binds to PD-1 [27]. Lee *et al*. reported that binding the nivolumab to PD-1 induces a conformational change in the BC loop, which is incompatible with PD-L1 binding mechanism [26]. Jeong *et al*. used crystallography experiments to show that the BC loop of PD-1 is a hotspot for binding of cemiplimab [79]. Previous reports showed that the C′D loop of PD-1 dominates the binding to several antibodies [21, 26, 28, 30, 58, 63, 77]. Based on the results in our study, the C′D loop entered in the groove that is formed by the CDR loops and induced conformational changes in the BC loop (S1 Video, design-3753), and this interaction could improve the binding affinity as the results, which is also confirmed by Lee et al study [26]. Mittal *et al*. employed MD simulations to show that the C′D loop of PD-1 in unbound and bound forms altered from the flexible to the rigid conformation, respectively, and helped to improve the binding affinity of the mAbs which is also confirmed our results [63].

Finally, our results indicated that CDRs of the antibodies cover the PD-L1 binding site and can outcompete anti-PD-1 antibodies to block the PD-1-PD-L1 interaction. In addition, as it is shown in the results, conformational changes of loops play a critical role in the interaction with the designed antibodies and these loops adopt different conformation compared when PD-1 bind to PD-L1. Although the binding sites of the antibodies on PD-1 are different, previous studies have shown that pembrolizumab, nivolumab, MW11-h317, mAb059c, sasanlimab, and tislelizumab antibodies block the interaction between PD-1 and PD-L1 by outcompeting PD-L1 for binding to PD-1 [21, 26, 27, 29–32, 77]. Based on the results, we suggest that the overlapping of antibodies binding sites (Fig 2) on PD-1 and conformational changes of FG, BC, CC′, and C′D loops (Fig 11) are incompatible with PD-L1 binding to PD-1 and can be reasons for mechanisms competing with PD-L1 for binding to PD-1 (design-3753 (S1 Video), design-9886 (S2 Video), design-9609 (S3 Video), design-7357 (S4 Video), design-3128 (S5 Video), design-8773 (S6 Video), and design-1223 (S7 Video)). For example, Zak *et al*. reported that the CC′ loop rearrangement in the form of a 90° twist and changed from the open to the closed conformation [3]. On the other hand, Hong *et al*. showed that the CC′ loop of PD-1 is a targetable region for binding to PD-L1, which uniquely binds to tislelizumab (binding site 7, Fig 2) and the binding site of PD-L1 blockage [32]. Consistent with previous reports, CDRs of design-1223 (binding site 7, Fig 2) bind to the CC’ loop of PD-1 (S22 and S36 Figs in S1 File) and can block the binding site of PD-L1 (S7 Video).

In addition, we found a rearrangement in the side chain of PD-L1 residues involved in its interaction with PD-1. However, the designed antibodies for PD-L1 receptor (similar to the designed antibodies for PD-1) did not significantly induce conformational changes within the structure of PD-L1. The paratopes of designs 7173, 4233, 5344, and 4503 occupy a large part of the PD-1 binding site (binding site 8–11, Fig 2), in addition, regarding the higher affinity of these designs, in comparison with the PD-1-PD-L1 interaction (Fig 3 and S2 Table in S1 File), it is expected that these designs could blockade the PD-1-PD-L1 interaction. In recent years, efforts have been made to develop PD-1/PD-L1 small-molecule inhibitors. Previous studies have shown that small-molecule inhibitors preferentially bind to the PD-1 binding site [14, 15]. Consistent with our results, Acúrcio *et al*. reported that Tyr56, Gln66, Met115, Ala121 Asp122, Tyr123, Lys124, and Arg125 are key interacting residues at the PD-1 binding site [14].

## Conclusion

Checkpoint inhibitor-based therapies are important for the treatment of cancer, which is the most common disease in humans. Previous studies have shown that *in vivo* antibody affinity maturation often fails in targeted therapy, which highlight the importance of computational design methods in this area. The present study aimed to improve the binding affinity of antibodies using affinity maturation technique to optimize the CDR sequences of the available chain in antibodies, employing Rosetta protocols and molecular dynamics simulation to understand the inhibitory mechanisms and specific binding characteristics of mAbs. In this study, 11 preclinical and pharmaceutical antibodies were selected as templates for designs, including pembrolizumab, nivolumab, toripalimab, MW11-h317, mAb059, sasanlimab, and tislelizumab for binding sites 1 to 7 on PD-1 receptor, BMS-936559, avelumab, durvalumab, and atezolizumab for binding sites 8 to 11 on PD-L1 receptor. 10,000 antibodies were designed for each binding site with a total number of 110,000 for all binding sites. Then, MD simulations have been done for the best 36 designs and 12 control complexes for binding sites 1–11 on PD-1 and PD-L1. Finally, 21 designs have been obtained for 11 binding sites. We showed that our designs cover different areas of the receptor binding site (PD-1 or PD-L1) and effectively block ligand-receptor interactions by steric occlusion. Binding sites 1, 3 and 6 for PD-1 and binding sites 9 and 11 for PD-L1 could be regarded as the best inhibition spots in PD-1-PD-L1 interaction. This study provides comprehensive information regarding the potential binding epitopes on PD-1, including the N, BC, C′D, and FG loops, which could be considered hotspots for designing potential biopharmaceuticals. In addition, our findings demonstrated that specific and collective point mutations in the CDRs sequences could rearrange the interaction pattern between antibody and receptor (PD-1 or PD-L1) and capable of changing the dynamics in the CDRs regions, enhancing structural stability and improving binding affinity. This study might be helpful for developing novel and more efficient antibodies against PD-1 or PD-L1 for checkpoint inhibition in immunotherapy.

## Supporting information

S1 File (DOCX)

S1 Video Conformational changes of PD-1 loops upon binding to design-3753 (binding site 1). (MP4)

S2 Video Conformational changes of PD-1 loops upon binding to design-9886 (binding site 2). (MP4)

S3 Video Conformational changes of PD-1 loops upon binding to design-9609 (binding site 3). (MP4)

S4 Video Conformational changes of PD-1 loops upon binding to design-7357 (binding site 4). (MP4)

S5 Video Conformational changes of PD-1 loops upon binding to design-3128 (binding site 5). (MP4)

S6 Video Conformational changes of PD-1 loops upon binding to design-8773 (binding site 6). (MP4)

S7 Video Conformational changes of PD-1 loops upon binding to design-1223 (binding site 7). (MP4)
