# Supplementary material for: An in silico investigation on the binding site preference of PD-1 and PD-L1 for designing antibodies for targeted cancer therapy
Source: PLoS One. 2024 Jul 25;19(7):e0304270. doi: 10.1371/journal.pone.0304270 (PMC11271968; doi:10.1371/journal.pone.0304270)
Supplement: S1 File — (DOCX) [file pone.0304270.s001.docx]

**Supplementary Information File**

**An *in silico* investigation on the binding site preference of PD-1 and PD-L1 for designing antibodies for targeted cancer therapy**

**Sarah Abdolmaleki^1^, Mazdak Ganjalikhani hakemi^2,3^, Mohamad Reza Ganjalikhany^1^***

^1^ Department of Cell and Molecular Biology & Microbiology, University of Isfahan, Isfahan, Iran

^2^ Regenerative and Restorative Medicine Research Center (REMER), Research Institute for Health Sciences and Technologies (SABITA), Istanbul Medipol University, Istanbul, Turkey

^3^ Department of Immunology, faculty of Medicine, Isfahan University of Medical Sciences, Isfahan, Iran

* Email: m.ganjalikhany@sci.ui.ac.ir

**Supplementary contents:**

S1 Fig: Redesigned CDR sequences for nivolumab in complex with PD-1 (control-5wt9).

**S2 Fig.** Redesigned CDR sequences for toripalimab in complex with PD-1 (control-6jbt).

**S3 Fig.** Redesigned CDR sequences for MW11-H317 in complex with PD-1 (control-6jjp).

**S4 Fig.** Redesigned CDR sequences for MAB059C in complex with PD-1 (control-6k0y).

**S5 Fig.** Redesigned CDR sequences for sasanlimab in complex with PD-1 (control-6xkr).

**S6 Fig.** Redesigned CDR sequences for tislelizumab in complex with PD-1 (control-7cgw).

**S7 Fig.** Redesigned CDR sequences for BMS936559 in complex with PD-L1 (control-5ggt).

**S8 Fig.** Redesigned CDR sequences for avelumab in complex with PD-L1 (control-5grj).

**S9 Fig.** Redesigned CDR sequences for durvalumab in complex with PD-L1 (control-5xj4).

**S10 Fig.** Redesigned CDR sequences for atezolizumab in complex with PD-L1 (control-5xxy).

**S11 Fig.** RMSD graphs for control-5wt9 (nivolumab-PD-1) complex during 50 ns of MD simulation.

**S12 Fig.** The RMSF graphs of PD-1 and antibodies during 50 ns of MD simulation (control-5wt9).

**S13 Fig.** RMSD graphs for control-6jbt (toripalimab_PD-1) during 50 ns of MD simulation.

**S14 Fig.** The RMSF graphs of PD-1 and antibodies during 50 ns of MD simulation (control-6jbt).

**S15 Fig.** RMSD graphs for control-6jjp (MW11-H317_PD-1) complex during 50 ns of MD simulation.

**S16 Fig.** The RMSF graphs of PD-1 and antibodies during 50 ns of MD simulation (control-6jjp).

**S17 Fig.** RMSD graphs for control-6k0y(MAB059C_PD-1) complex during 50 ns of MD simulation.

**S18 Fig.** The RMSF graphs of PD-1 and antibodies during 50 ns of MD simulation (control-6k0y).

**S19 Fig.** RMSD graphs for control-6xkr (sasanlimab_PD-1) complex during 50 ns of MD simulation.

**S20 Fig.** The RMSF graphs of PD-1 and antibodies during 50 ns of MD simulation (control-6xkr).

**S21 Fig.** RMSD graphs for control-7cgw (tislelizumab_PD-1) complex during 50 ns of MD simulation.

**S22 Fig.** The RMSF graphs of PD-1 and antibodies during 50 ns of MD simulation (control-7cgw).

**S23 Fig.** RMSD graphs for control-5ggt (BMS936559_PD-L1) complex during 50 ns of MD simulation.

**S24 Fig.** The RMSF graphs of PD-1 and antibodies during 50 ns of MD simulation (control-5ggt).

**S25 Fig.** RMSD graphs for control-5grj (avelumab_PD-L1) complex during 50 ns of MD simulation.

**S26 Fig.** The RMSF graphs of PD-1 and antibodies during 50 ns of MD simulation (control-5grj).

**S27 Fig.** RMSD graphs for control-5xj4 (durvalumab_PD-L1) complex during 50 ns of MD simulation.

**S28 Fig.** The RMSF graphs of PD-1 and antibodies during 50 ns of MD simulation (control-5xj4).

**S29 Fig.** RMSD graphs for control-5xxy (atezolizumab_PD-L1) complex during 50 ns of MD simulation.

**S30 Fig.** The RMSF graphs of PD-1 and antibodies during 50 ns of MD simulation (control-5xxy).

**S31 Fig.** Contact patterns of complexes during 50 ns of MD simulation.

**S32 Fig.** Contact patterns of complexes during 50 ns of MD simulation.

**S33 Fig.** Contact patterns of complexes during 50 ns of MD simulation.

**S34 Fig.** Contact patterns of complexes during 50 ns of MD simulation.

**S35 Fig.** Contact patterns of complexes during 50 ns of MD simulation.

**S36 Fig.** Contact patterns of complexes during 50 ns of MD simulation.

**S37 Fig.** Contact patterns of complexes during 50 ns of MD simulation.

**S38 Fig.** Contact patterns of complexes during 50 ns of MD simulation.

**S39 Fig.** Contact patterns of complexes during 50 ns of MD simulation.

**S40 Fig.** Contact patterns of complexes during 50 ns of MD simulation.

**S41 Fig.** 2D interaction maps of heavy and light chains of antibodies in complex with PD-1 (control-5wt9).

**S42 Fig.** 2D interaction maps of heavy and light chains of antibodies in complex with PD-1 (control-6jbt).

**S43 Fig.** 2D interaction maps of heavy and light chains of antibodies in complex with PD-1 (control-6jjp).

**S44 Fig.** 2D interaction maps of heavy and light chains of antibodies in complex with PD-1 (control-6k0y).

**S45 Fig.** 2D interaction maps of heavy and light chains of antibodies in complex with PD-1 (control-6xkr).

**S46 Fig.** 2D interaction maps of heavy and light chains of antibodies in complex with PD-1 (control-7cgw).

**S47 Fig.** 2D interaction maps of heavy and light chains of antibodies in complex with PD-L1 (control-5ggt).

**S48 Fig.** 2D interaction maps of heavy and light chains of antibodies in complex with PD-L1 (control-5grj).

**S49 Fig.** 2D interaction maps of heavy and light chains of antibodies in complex with PD-L1 (control-5xj4).

**S50 Fig.** 2D interaction maps of heavy and light chains of antibodies in complex with PD-L1 (control-5xxy).

**S51 Fig.** Binding free energy decomposition of the residues in the control and designed complexes (control-5wt9).

**S52 Fig.** Binding free energy decomposition of the residues in the control and designed complexes (control-6jbt).

**S53 Fig.** Binding free energy decomposition of the residues in the control and designed complexes (control-6jjp).

**S54 Fig.** Binding free energy decomposition of the residues in the control and designed complexes (control-6k0y).

**S55 Fig.** Binding free energy decomposition of the residues in the control and designed complexes (control-6xkr).

**S56 Fig.** Binding free energy decomposition of the residues in the control and designed complexes (control-7cgw).

**S57 Fig.** Binding free energy decomposition of the residues in the control and designed complexes (control-5ggt).

**S58 Fig.** Binding free energy decomposition of the residues in the control and designed complexes (control-5grj).

**S59 Fig.** Binding free energy decomposition of the residues in the control and designed complexes (control-5xj4).

**S60 Fig.** Binding free energy decomposition of the residues in the control and designed complexes (control-5xxy).

**S61 Fig.**Analysis of conformational changes within PD-1 loops in the design-9886 complex.

**S62 Fig.**Analysis of conformational changes within PD-1 loops in the design-9609 complex.

**S63 Fig.**Analysis of conformational changes within PD-1 loops in the design-7357 complex.

**S64 Fig.**Analysis of conformational changes within PD-1 loops in the design-3128 complex.

**S65 Fig.**Analysis of conformational changes within PD-1 loops in the design-8773 complex.

**S66 Fig.**Analysis of conformational changes within PD-1 loops in the design-1223 complex.

**S67 Fig.** RMSD graphs for PD-1 with and without loops during 50 ns of MD simulation (control-5ggs).

**S68 Fig.** RMSD graphs for PD-1 with and without loops during 50 ns of MD simulation (control-5wt9).

**S69 Fig.** RMSD graphs for PD-1 with and without loops during 50 ns of MD simulation (control-6jbt).

**S70 Fig.** RMSD graphs for PD-1 with and without loops during 50 ns of MD simulation (control-6jjp).

**S71 Fig.** RMSD graphs for PD-1 with and without loops during 50 ns of MD simulation (control-6k0y).

**S72 Fig.** RMSD graphs for PD-1 with and without loops during 50 ns of MD simulation (control-6xkr).

**S73 Fig.** RMSD graphs for PD-1 with and without loops during 50 ns of MD simulation (control-7cgw).

**S1 Table.** The interface characteristics of the antibody-PD-1 and antibody-PD-L1 complexes were calculated by RosettaInterfaceAnalyzer.

**S2 Table.** MMPBSA binding energies (kcal·mol ^-1^) for control and design complexes.

**S3 Table.** The types of interactions between the CDRs of the designs and binding sites 1-11 during simulations.

**S4 Table .** List of key residues of PD-1 and PD-L1 receptors at binding sites 1-11.

**S5 Table.** The binding free energy differences between the mutations and residues of designs and control in binding site 1 (kcal.mol^-1^).

**S1 Video.** Conformational changes of PD-1 loops upon binding to design-3753 (binding site 1).

**S2 Video.** Conformational changes of PD-1 loops upon binding to design-9886 (binding site 2).

**S3 Video.** Conformational changes of PD-1 loops upon binding to design-9609 (binding site 3).

**S4 Video.** Conformational changes of PD-1 loops upon binding to design-7357 (binding site 4).

**S5 Video.** Conformational changes of PD-1 loops upon binding to design-3128 (binding site 5).

**S6 Video.** Conformational changes of PD-1 loops upon binding to design-8773 (binding site 6).

**S7 Video.** Conformational changes of PD-1 loops upon binding to design-1223 (binding site 7).


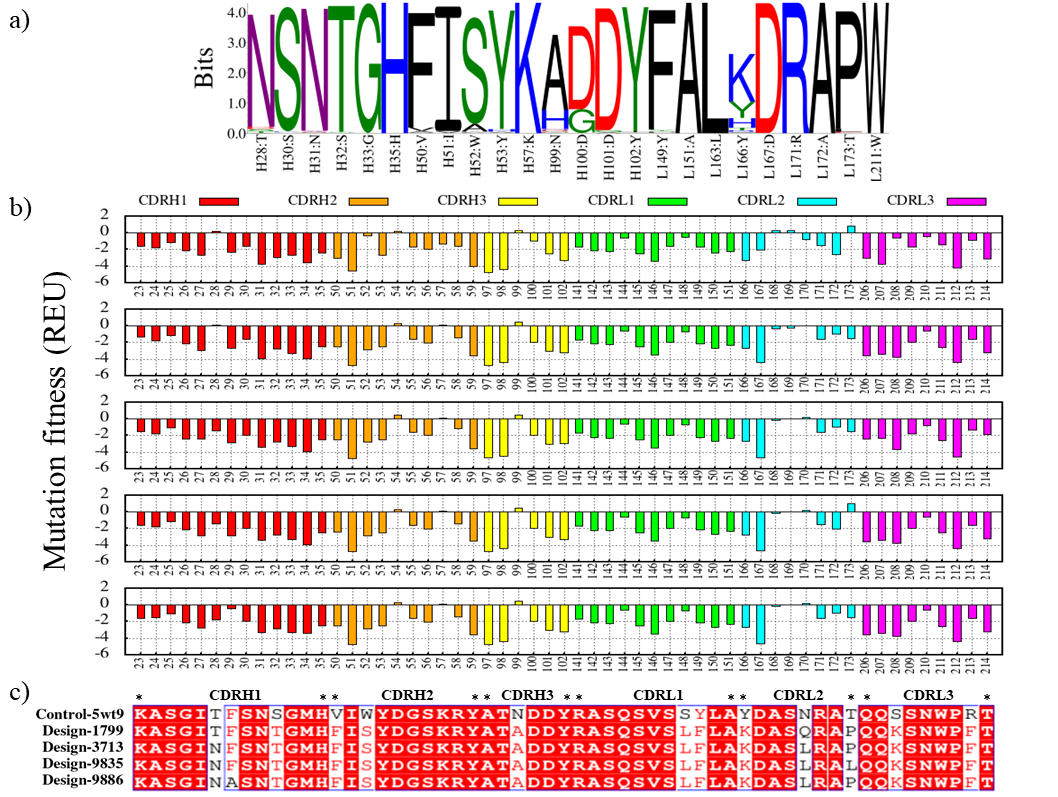


**Fig S1. Redesigned CDR sequences for nivolumab in complex with PD-1 (control-5wt9).** (a) The sequence logo in the 10000 designs. (b) Mutational fitness analysis of each residue in nivolumab CDR sequences. Mutational energy shown as a function of individual mutations; a more negative energy score is preferred. The CDRH1, CDRH2, CDRH3, CDRL1, CDRL2, and CDRL3 were colored in red, orange, yellow, green, cyan, and magenta, respectively. (c) Alignment of CDR sequences in the control-5wt9 and design groups.


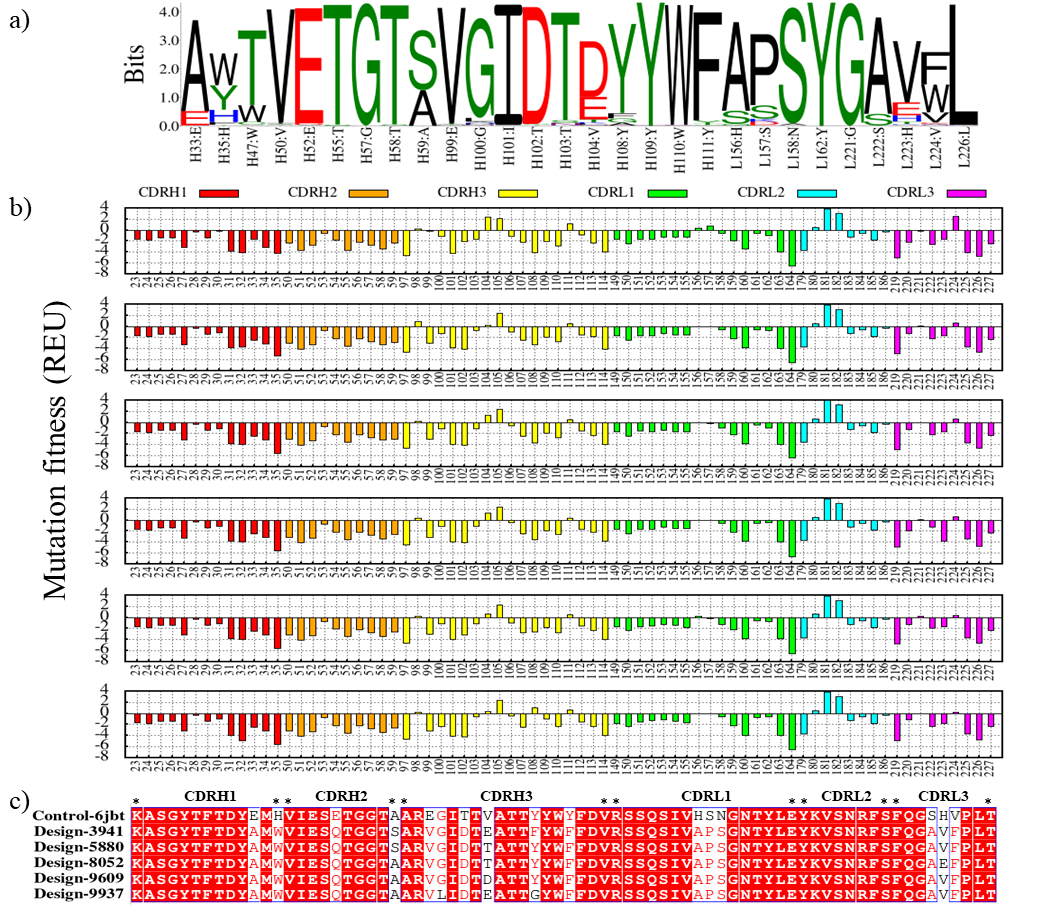


**S2 Fig. Redesigned CDR sequences for** **toripalimab in complex with PD-1 (control-6jbt).** (a) The sequence logo in the 10,000 designs. (b) Mutational fitness analysis of each residue in toripalimab CDR sequences. Mutational energy shown as a function of individual mutations; a more negative energy score is preferred. The CDRH1, CDRH2, CDRH3, CDRL1, CDRL2, and CDRL3 were colored in red, orange, yellow, green, cyan, and magenta, respectively. (c) Alignment of CDR sequences in the control-6jbt and design groups.


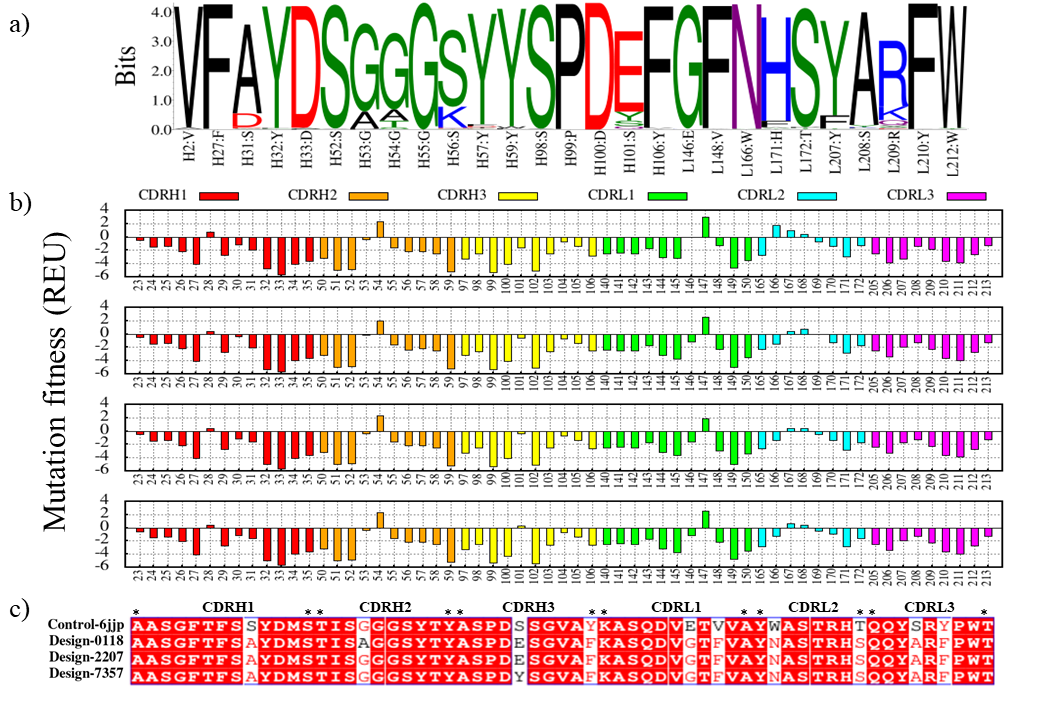


**S3 Fig. Redesigned CDR sequences for** **MW11-H317 in complex with PD-1 (control-6jjp).** (a) The sequence logo in the 10,000 designs. (b) Mutational fitness analysis of each residue in MW11-H317 CDR sequences. Mutational energy shown as a function of individual mutations; a more negative energy score is preferred. The CDRH1, CDRH2, CDRH3, CDRL1, CDRL2, and CDRL3 were colored in red, orange, yellow, green, cyan, and magenta, respectively. (c) Alignment of CDR sequences in the control-6jjp and design groups.


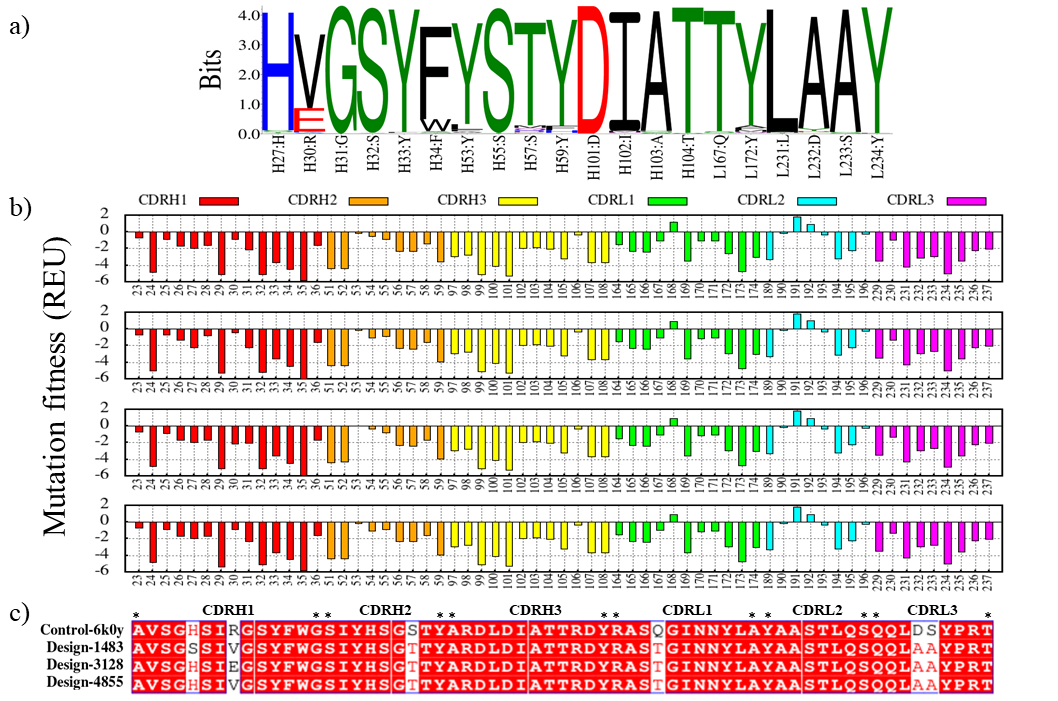


**S4 Fig. Redesigned CDR sequences for** **MAB059C in complex with PD-1 (control-6k0y).** (a) The sequence logo in the 10,000 designs. (b) Mutational fitness analysis of each residue in MAB059C CDR sequences. Mutational energy shown as a function of individual mutations; a more negative energy score is preferred. The CDRH1, CDRH2, CDRH3, CDRL1, CDRL2, and CDRL3 were colored in red, orange, yellow, green, cyan, and magenta, respectively. (c) Alignment of CDR sequences in the control-6k0y and design groups.


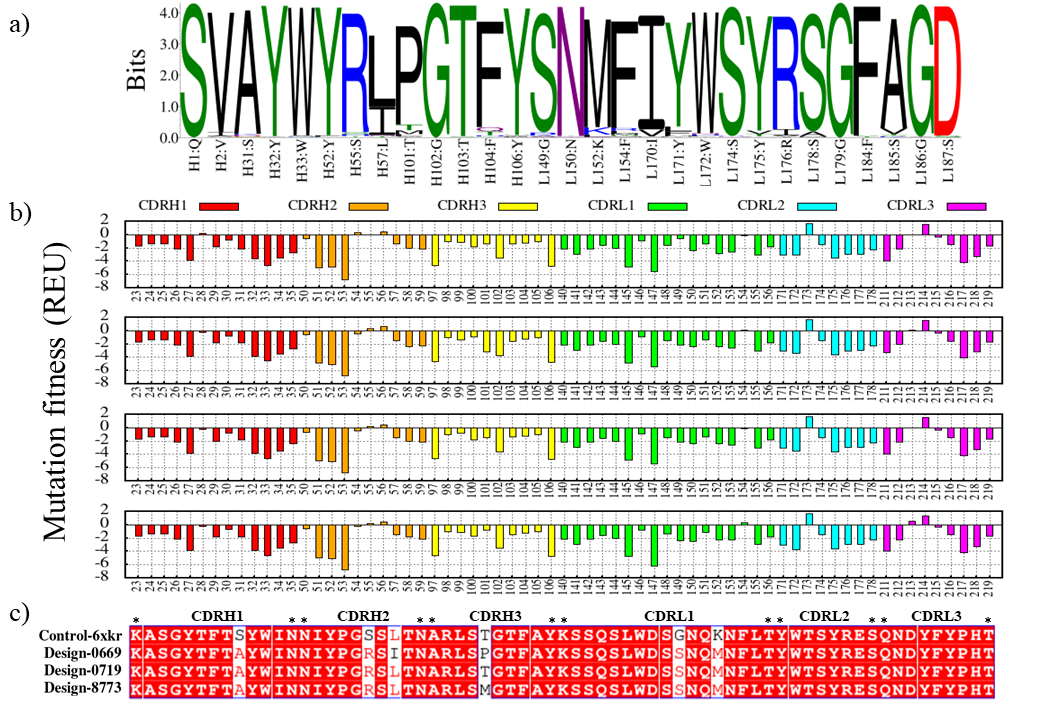


**S5 Fig. Redesigned CDR sequences for** **sasanlimab in complex with PD-1 (control-6xkr).** (a) The sequence logo in the 10,000 designs. (b) Mutational fitness analysis of each residue in sasanlimab CDR sequences. Mutational energy shown as a function of individual mutations; a more negative energy score is preferred. The CDRH1, CDRH2, CDRH3, CDRL1, CDRL2, and CDRL3 were colored in red, orange, yellow, green, cyan, and magenta, respectively. (c) Alignment of CDR sequences in the control-6xkr and design groups.


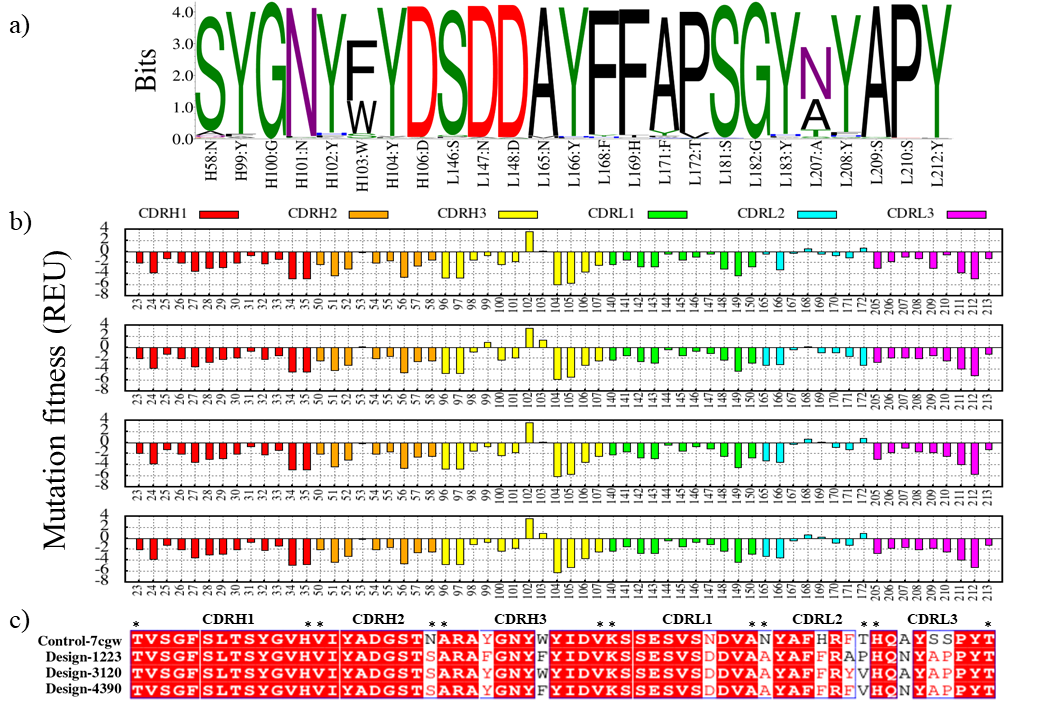


**S6 Fig. Redesigned CDR sequences for** **tislelizumab in complex with PD-1 (control-7cgw).** (a) The sequence logo in the 10,000 designs. (b) Mutational fitness analysis of each residue in tislelizumab CDR sequences. Mutational energy shown as a function of individual mutations; a more negative energy score is preferred. The CDRH1, CDRH2, CDRH3, CDRL1, CDRL2, and CDRL3 were colored in red, orange, yellow, green, cyan, and magenta, respectively. (c) Alignment of CDR sequences in the control-7cgw and design groups.


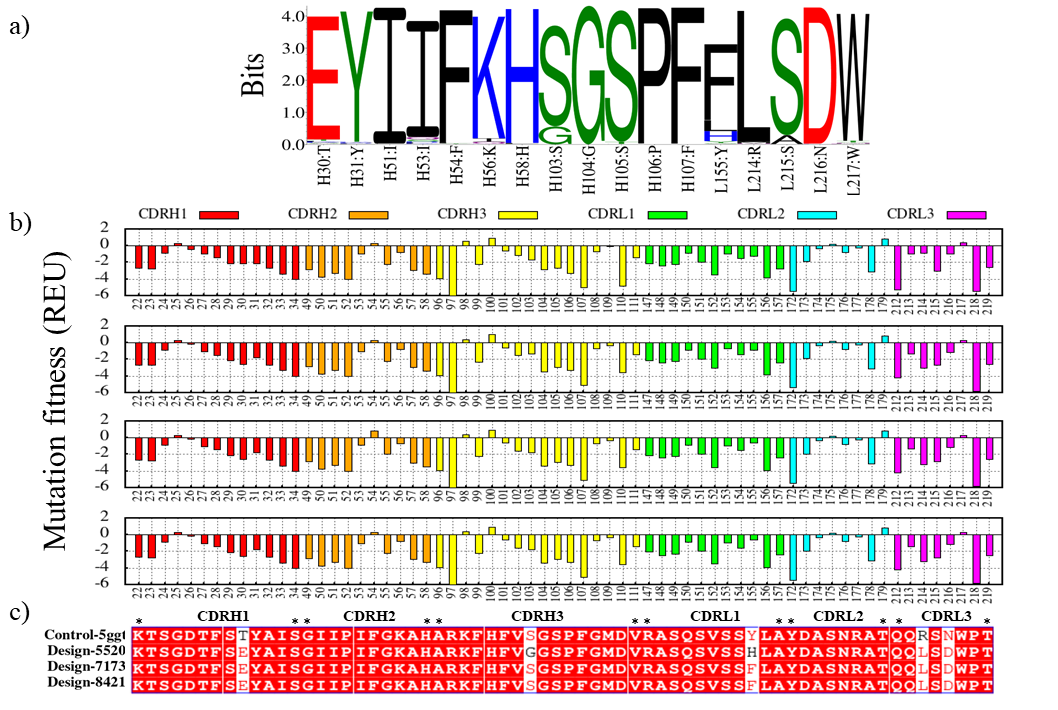


**S7 Fig. Redesigned CDR sequences for** **BMS936559 in complex with PD-1 (control-5ggt).** (a) The sequence logo in the 10,000 designs. (b) Mutational fitness analysis of each residue in BMS936559 CDR sequences. Mutational energy shown as a function of individual mutations; a more negative energy score is preferred. The CDRH1, CDRH2, CDRH3, CDRL1, CDRL2, and CDRL3 were colored in red, orange, yellow, green, cyan, and magenta, respectively. (c) Alignment of CDR sequences in the control-5ggt and design groups.


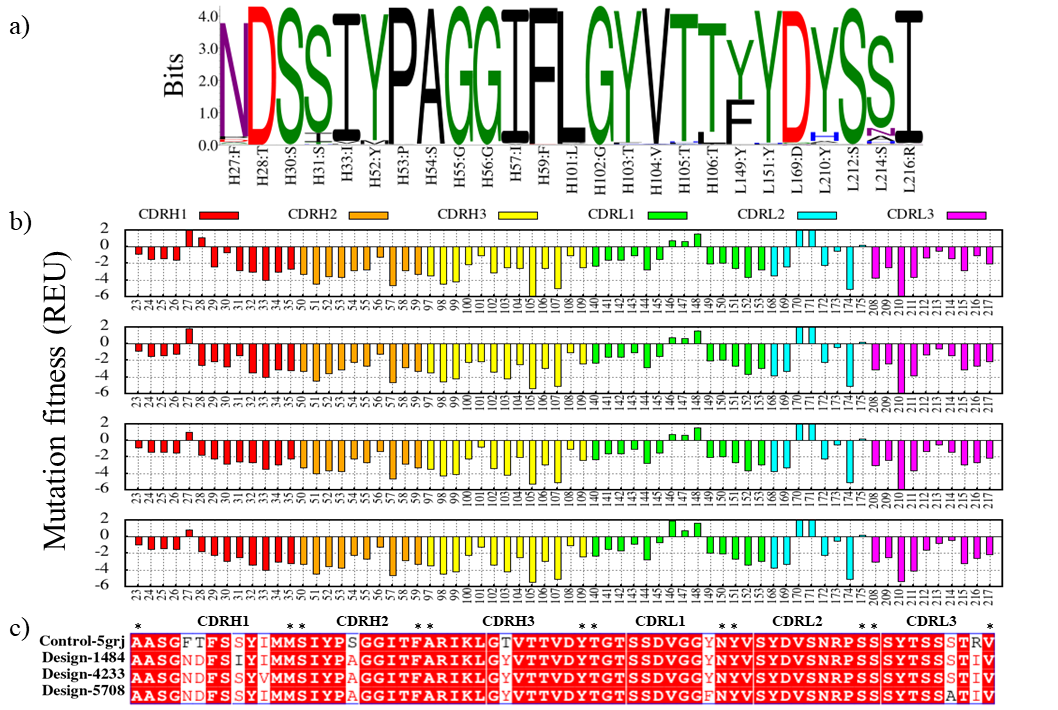


**S8 Fig. Redesigned CDR sequences for** **avelumab in complex with PD-1 (control-5grj).** (a) The sequence logo in the 10,000 designs. (b) Mutational fitness analysis of each residue in avelumab CDR sequences. Mutational energy shown as a function of individual mutations; a more negative energy score is preferred. The CDRH1, CDRH2, CDRH3, CDRL1, CDRL2, and CDRL3 were colored in red, orange, yellow, green, cyan, and magenta, respectively. (c) Alignment of CDR sequences in the control-5grj and design groups.


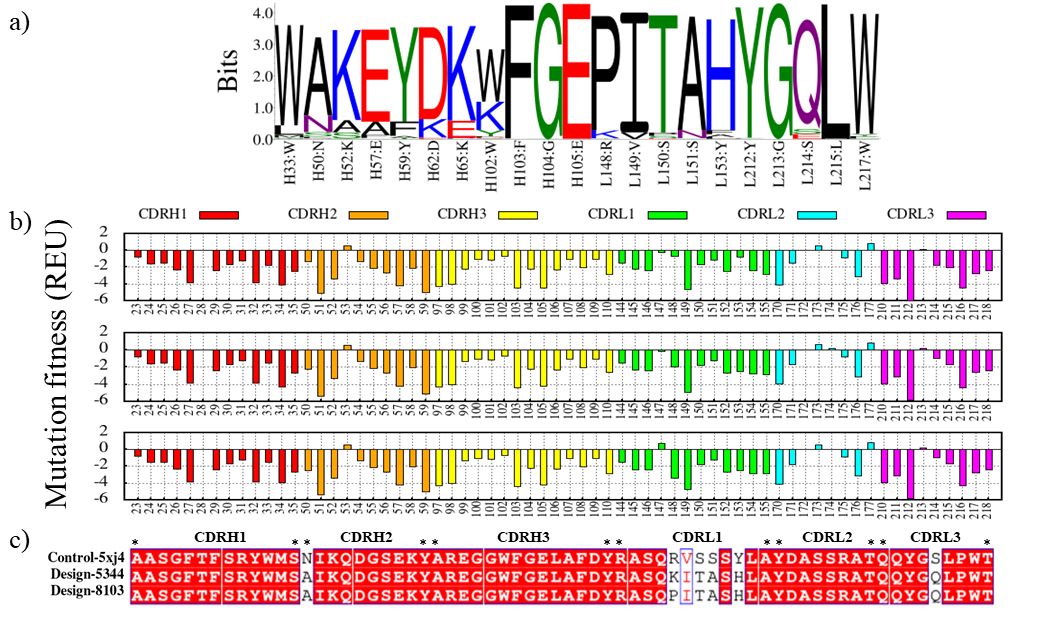


**S9 Fig. Redesigned CDR sequences for** **durvalumab in complex with PD-1 (control-5xj4).** (a) The sequence logo in the 10,000 designs. (b) Mutational fitness analysis of each residue in durvalumab CDR sequences. Mutational energy shown as a function of individual mutations; a more negative energy score is preferred. The CDRH1, CDRH2, CDRH3, CDRL1, CDRL2, and CDRL3 were colored in red, orange, yellow, green, cyan, and magenta, respectively. (c) Alignment of CDR sequences in the control-5xj4 and design groups.


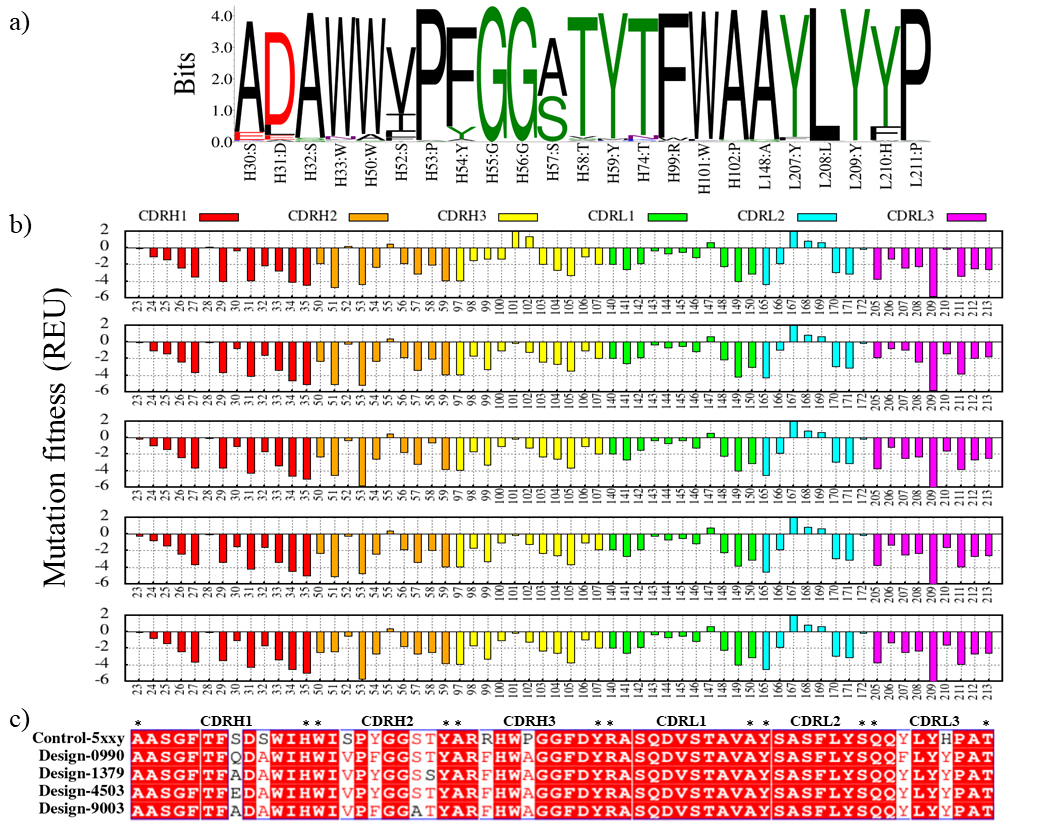


**S10 Fig. Redesigned CDR sequences for** **atezolizumab in complex with PD-1 (control-5xxy).** (a) The sequence logo in the 10,000 designs. (b) Mutational fitness analysis of each residue in atezolizumab CDR sequences. Mutational energy shown as a function of individual mutations; a more negative energy score is preferred. The CDRH1, CDRH2, CDRH3, CDRL1, CDRL2, and CDRL3 were colored in red, orange, yellow, green, cyan, and magenta, respectively. (c) Alignment of CDR sequences in the control-5xxy and design groups.


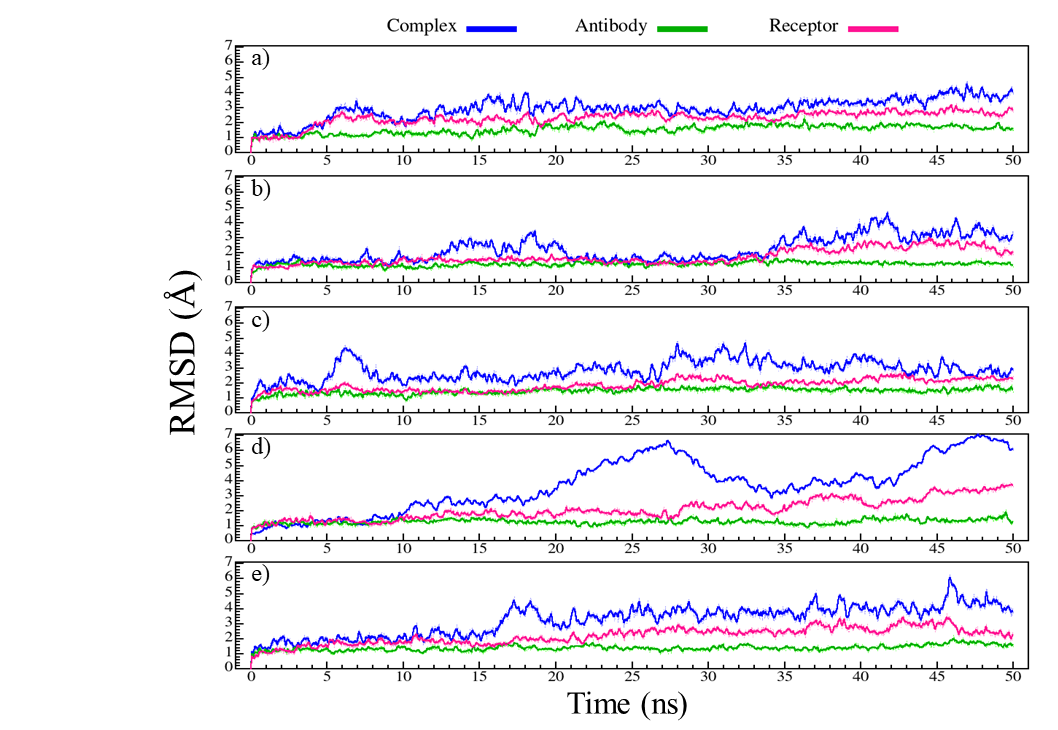


**S11 Fig. RMSD graphs for complex (blue), antibody (green), and PD-1 (magenta) during 50 ns of MD simulation.** (a) control-5wt9 (nivolumab-PD-1). (b) design-1799. (c) design-3713. (d) design-9835. (e) design-9886.


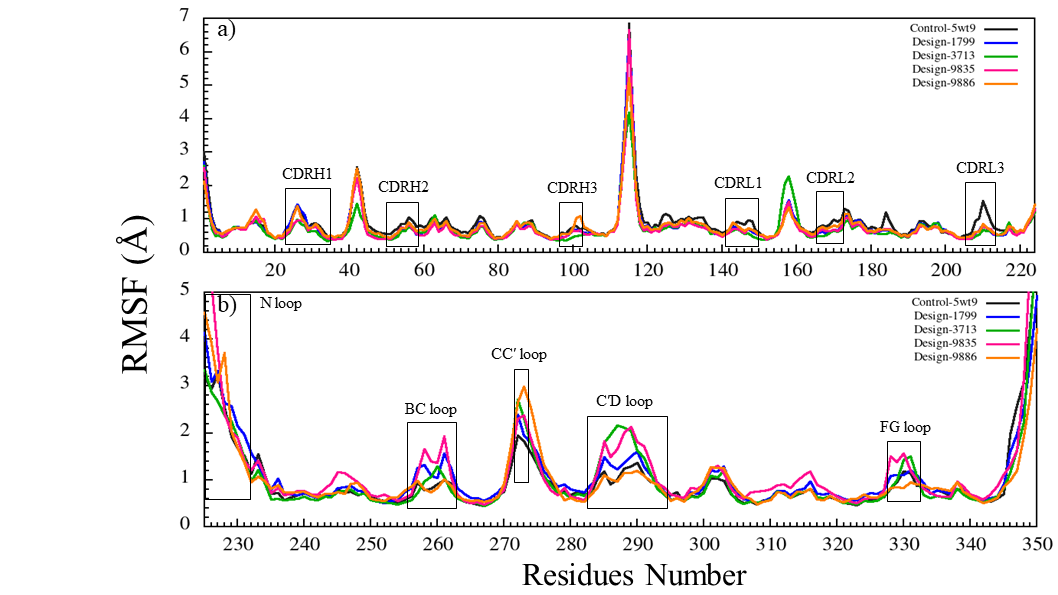


**S12 Fig. The RMSF graphs of PD-1 and antibodies during 50 ns of MD simulation.** RMSF graphs of PD-1 for control-5wt9 (nivolumab-PD-1, dark gray), design-1799 (blue), design-3713 (green), design-9835 (magenta), and design-9886 (orange). (a) Comparison of RMSF graphs of antibodies bound to PD-1. (b) Comparison of RMSF graphs of PD-1 when bound to antibodies.


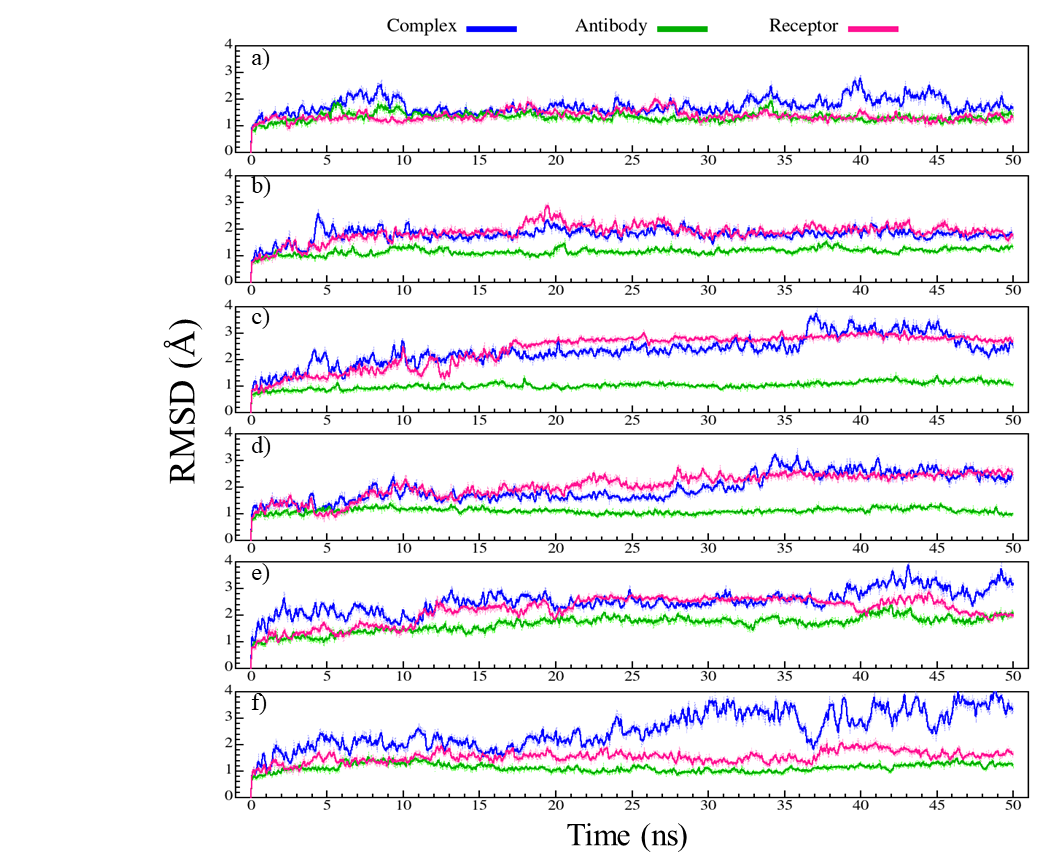


**S13 Fig. RMSD graphs for complex (blue), antibody (green), and PD-1 (magenta) during 50 ns of MD simulation.** (a) control-6jbt (toripalimab-PD-1). (b) design-3941. (c) design-5880. (d) design-8052. (e) design-9609. (f) design-9937.


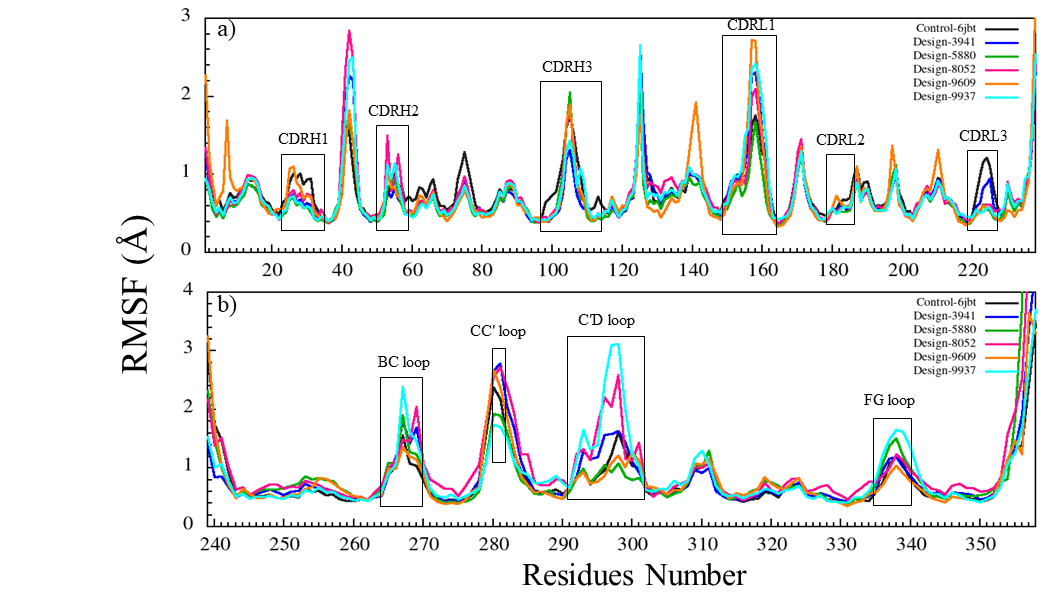


**S14 Fig. The RMSF graphs of PD-1 and antibodies during 50 ns of MD simulation.** RMSF graphs of PD-1 for control-6jbt (toripalimab-PD-1, dark gray), design-3941 (blue), design-5880 (green), design-8052 (magenta), design-9609 (orange), and design-9937 (cyan). (a) Comparison of RMSF graphs of antibodies bound to PD-1. (b) Comparison of RMSF graphs of PD-1 when bound to antibodies.


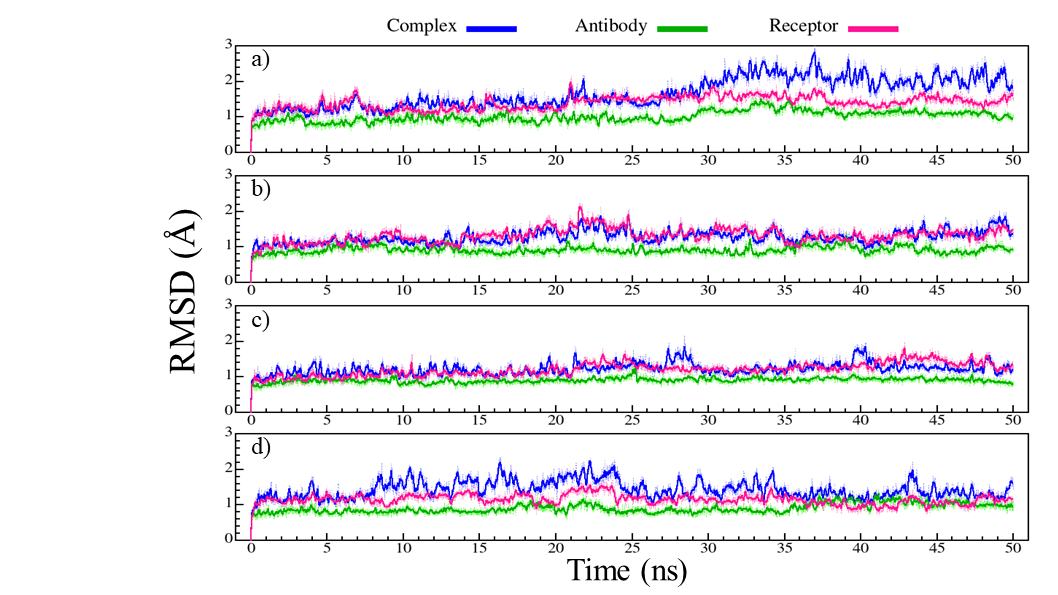


**S15 Fig. RMSD graphs for complex (blue), antibody (green), and PD-1 (magenta) during 50 ns of MD simulation.** (a) control-6jjp (MW11-h317-PD-1). (b) design-0118. (c) design-2207. (d) design-7357.


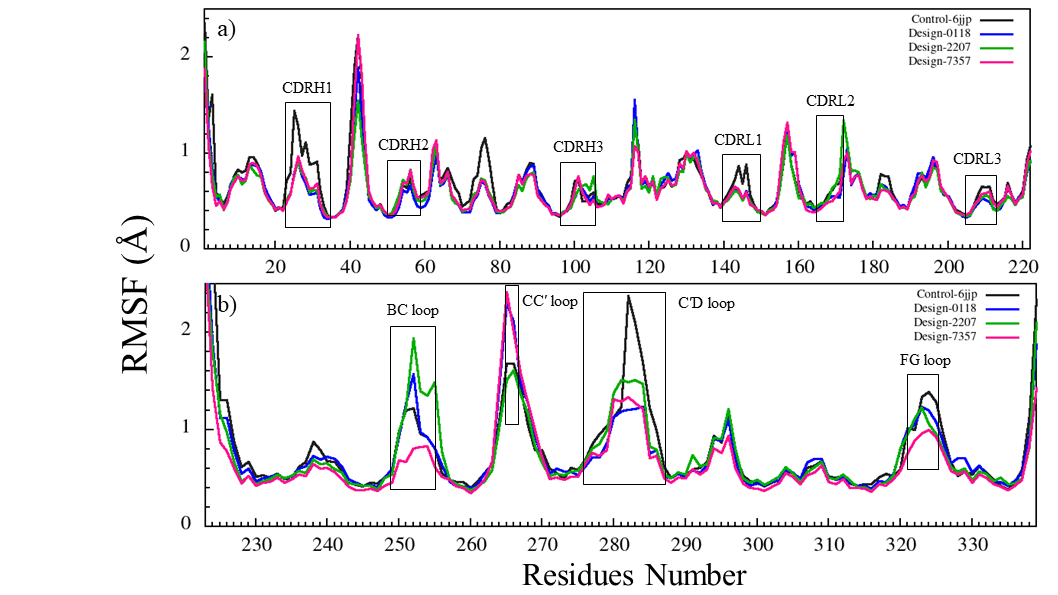


**S16 Fig. The RMSF graphs of PD-1 and antibodies during 50 ns of MD simulation.** RMSF graphs of PD-1 for control-6jjp (MW11-h317-PD-1, dark gray), design-0118 (blue), design-2207 (green), design-7357 (magenta). (a) Comparison of RMSF graphs of antibodies bound to PD-1. (b) Comparison of RMSF graphs of PD-1 when bound to antibodies.


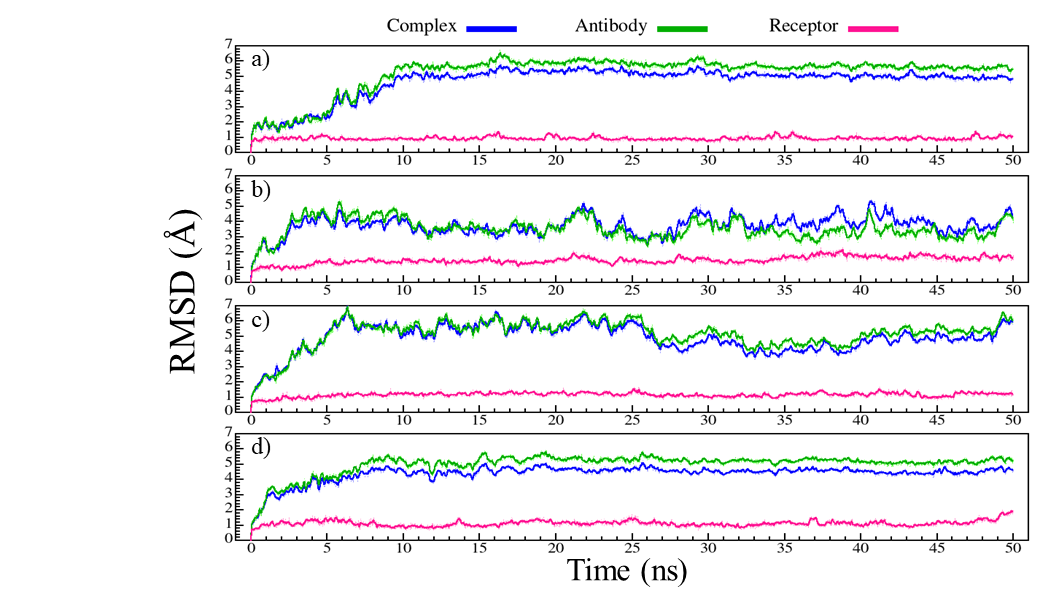


**S17 Fig. RMSD graphs for complex (blue), antibody (green), and PD-1 (magenta) during 50 ns of MD simulation.** (a) control-6k0y (mAb059c-PD-1). (b) design-1483. (c) design-3128. (d) design-4855.


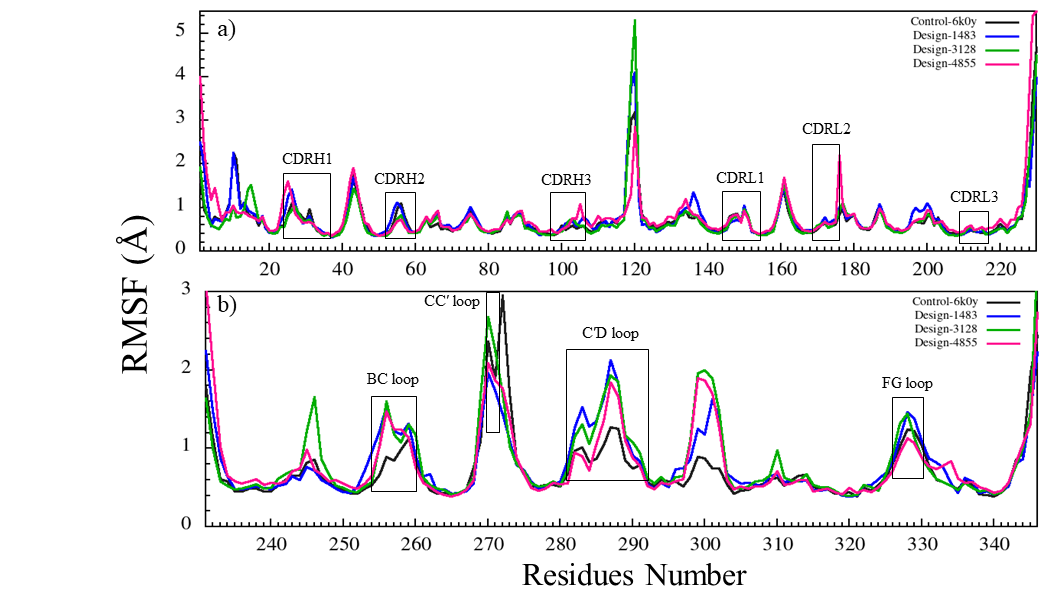


**S18 Fig. The RMSF graphs of PD-1 and antibodies during 50 ns of MD simulation.** RMSF graphs of PD-1 for control-6k0y (mAb059c-PD-1, dark gray), design-1483 (blue), design-3128 (green), design-4855 (magenta). (a) Comparison of RMSF graphs of antibodies bound to PD-1. (b) Comparison of RMSF graphs of PD-1 when bound to antibodies.


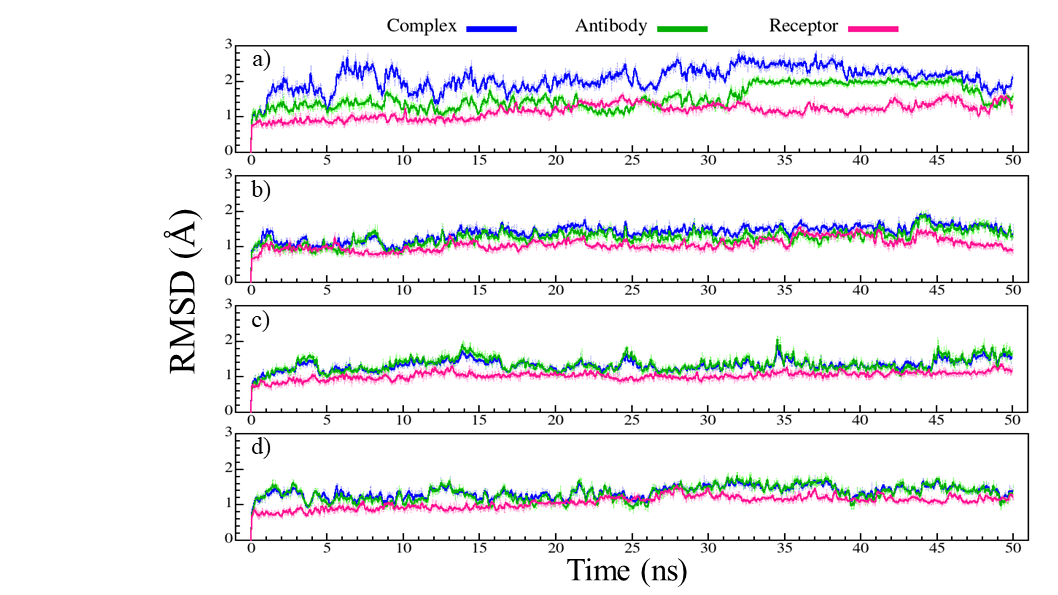


**S19 Fig. RMSD graphs for complex (blue), antibody (green), and PD-1 (magenta) during 50 ns of MD simulation.** (a) control-6xkr (sasanlimab-PD-1). (b) design-0669. (c) design-0719. (d) design-8773.


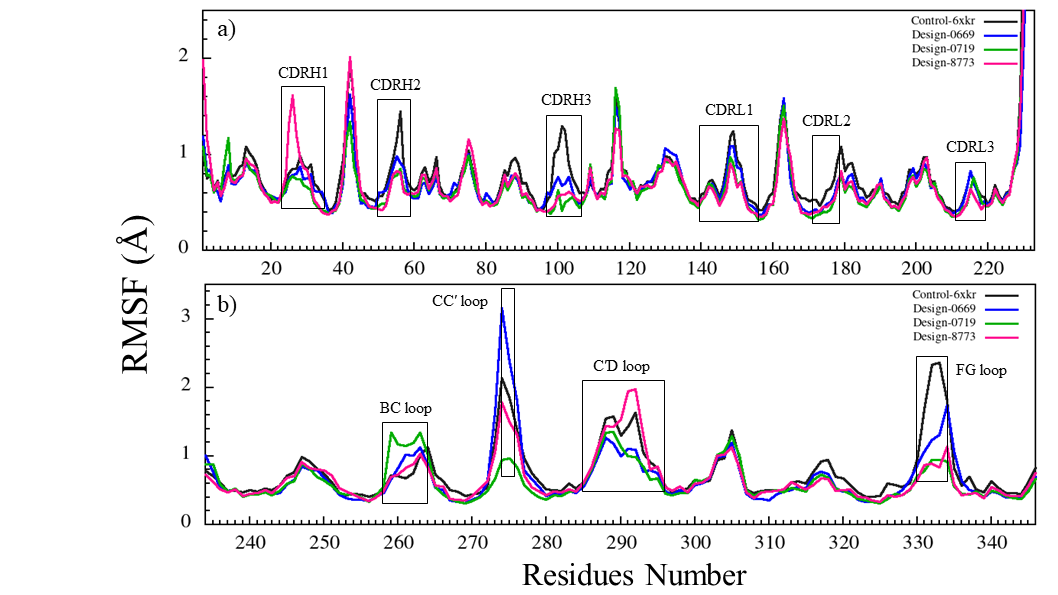


**S20 Fig. The RMSF graphs of PD-1 and antibodies during 50 ns of MD simulation.** RMSF graphs of PD-1 for control-6xkr (sasanlimaPD-1, dark gray), design-0669 (blue), design-0719 (green), design-8773 (magenta). (a) Comparison of RMSF graphs of antibodies bound to PD-1. (b) Comparison of RMSF graphs of PD-1 when bound to antibodies.


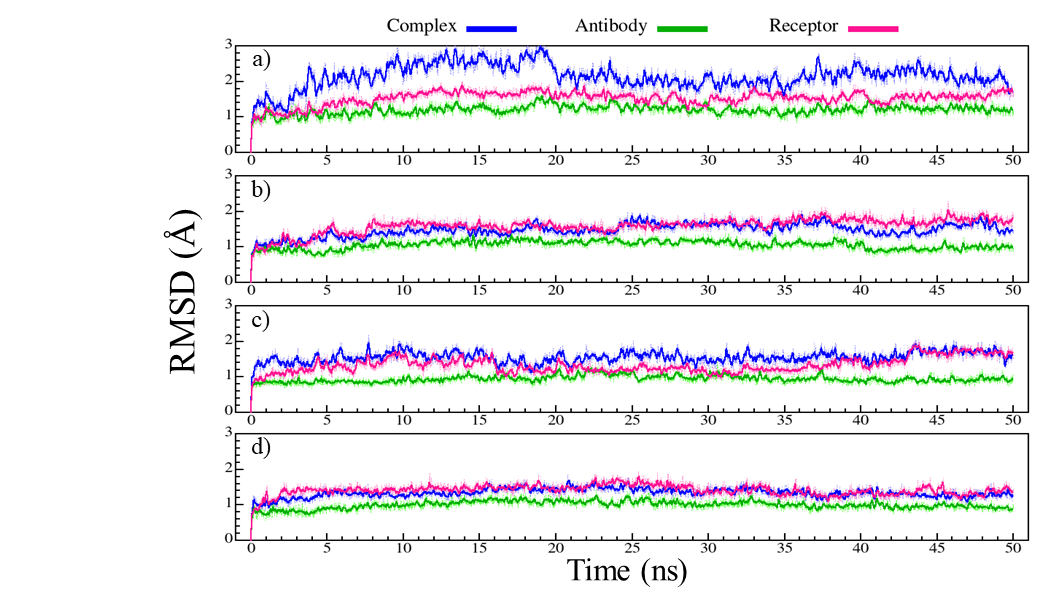


**S21 Fig. RMSD graphs for complex (blue), antibody (green), and PD-1 (magenta) during 50 ns of MD simulation.** (a) control-7cgw (tislelizumab-PD-1). (b) design-1223. (c) design-3120. (d) design-4390.


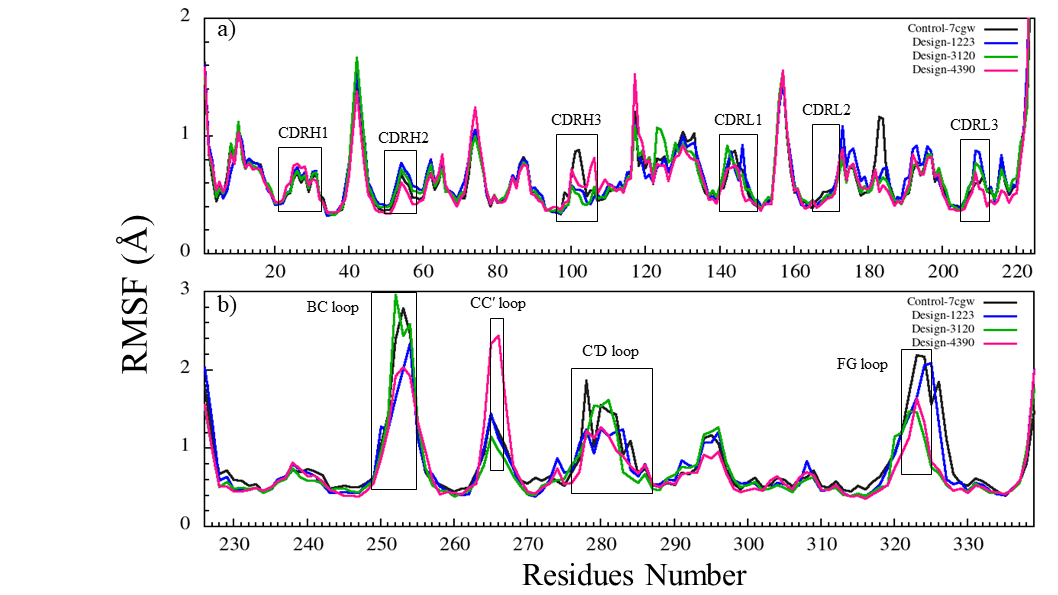


**S22 Fig. The RMSF graphs of PD-1 and antibodies during 50 ns of MD simulation.** RMSF graphs of PD-1 for control-7cgw (tislelizumab-PD-1, dark gray), design-1223 (blue), design-3120 (green), design-4390 (magenta). (b) Comparison of RMSF graphs of PD-1 when bound to antibodies.


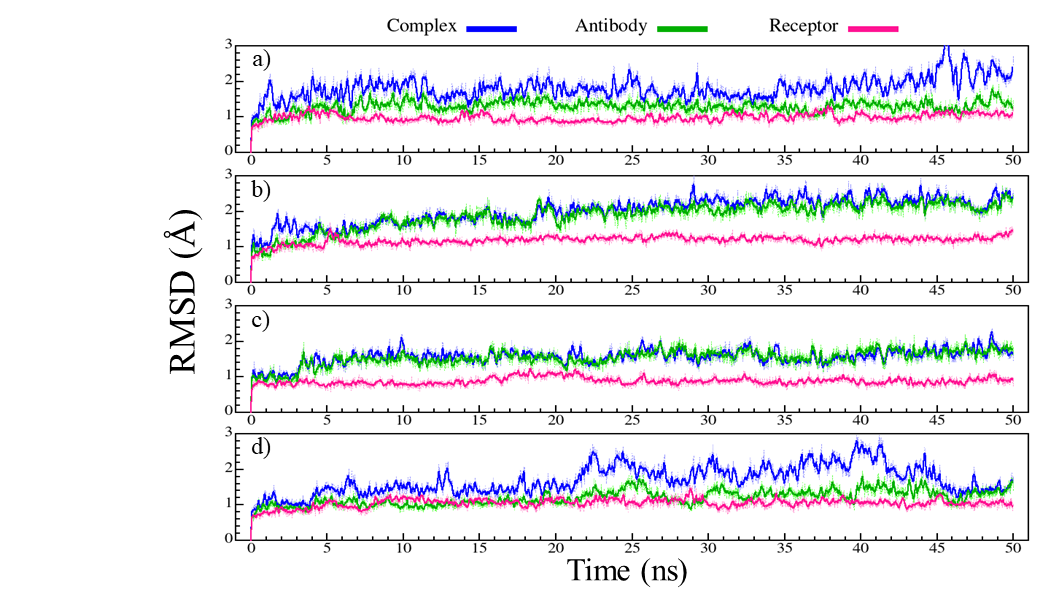


**S23 Fig. RMSD graphs for complex (blue), antibody (green), and PD-1 (magenta) during 50 ns of MD simulation.** (a) control-5ggt (BMS-936559-PD-L1). (b) design-5520. (c) design-7173. (d) design-8421.


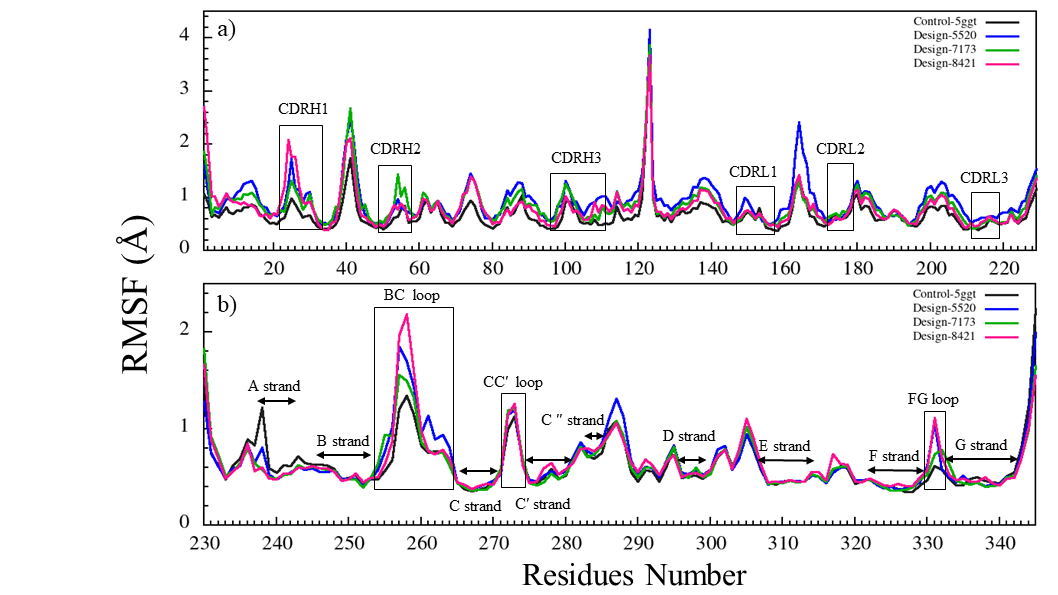


**S24 Fig. The RMSF graphs of PD-1 and antibodies during 50 ns of MD simulation.** RMSF graphs of PD-1 for control-5ggt (BMS-936559-PD-L1, dark gray), design-5520 (blue), design-7173 (green), design-8421 (magenta). (b) Comparison of RMSF graphs of PD-1 when bound to antibodies.


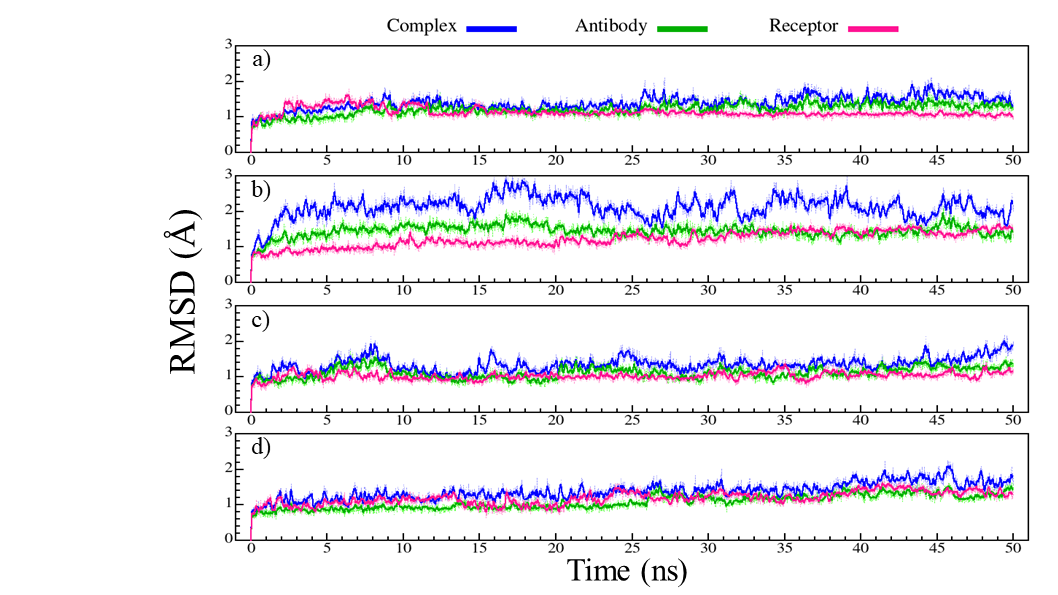


**S25 Fig. RMSD graphs for complex (blue), antibody (green), and PD-1 (magenta) during 50 ns of MD simulation.** (a) control-5grj (avelumab-PD-L1). (b) design-1484. (c) design-4233. (d) design-5708.


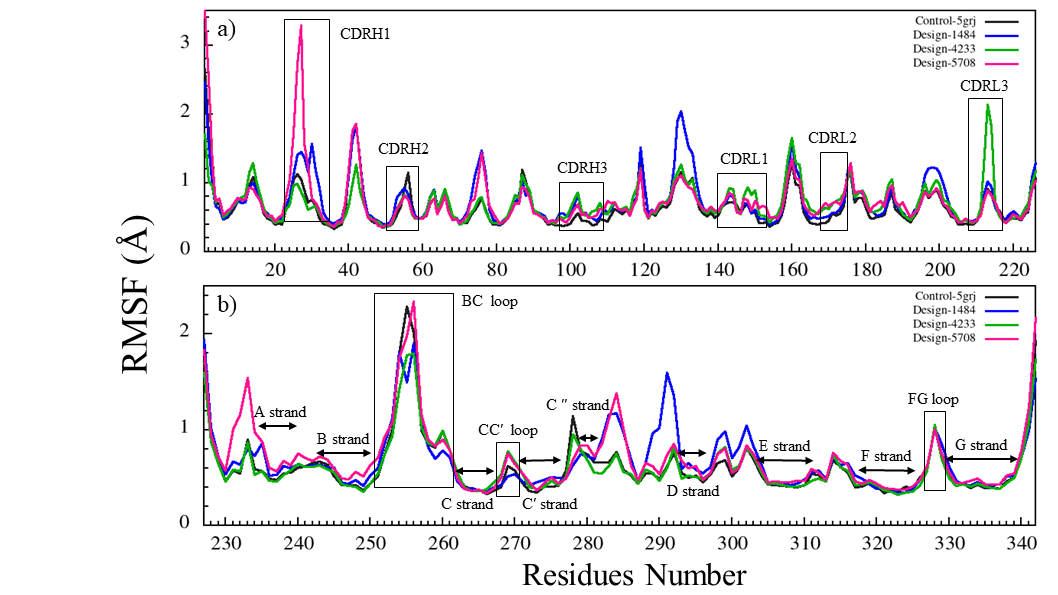


**S26 Fig. The RMSF graphs of PD-1 and antibodies during 50 ns of MD simulation.** RMSF graphs of PD-1 for control-5grj (avelumab-PD-L1, dark gray), design-1484 (blue), design-4233 (green), design-5708 (magenta). (b) Comparison of RMSF graphs of PD-1 when bound to antibodies.


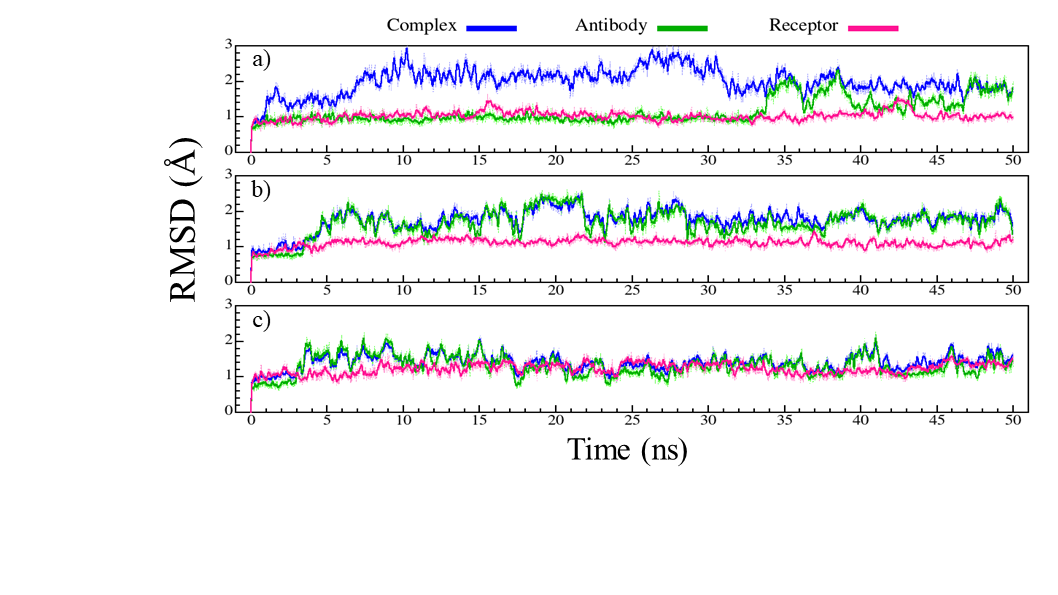


**S27 Fig. RMSD graphs for complex (blue), antibody (green), and PD-1 (magenta) during 50 ns of MD simulation.** (a) control-5xj4 (durvalumab-PD-L1). (b) design-5344. (c) design-8103.


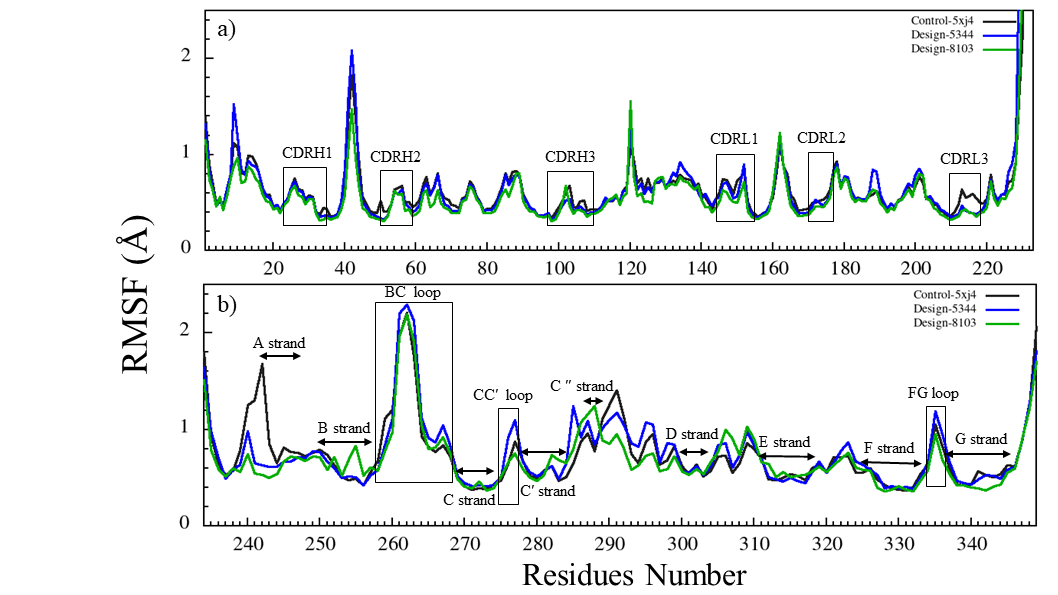


**S28 Fig. The RMSF graphs of PD-1 and antibodies during 50 ns of MD simulation.** RMSF graphs of PD-1 for control-5xj4 (durvalumab-PD-L1, dark gray), design-5344 (blue), design-8103 (green). (b) Comparison of RMSF graphs of PD-1 when bound to antibodies.


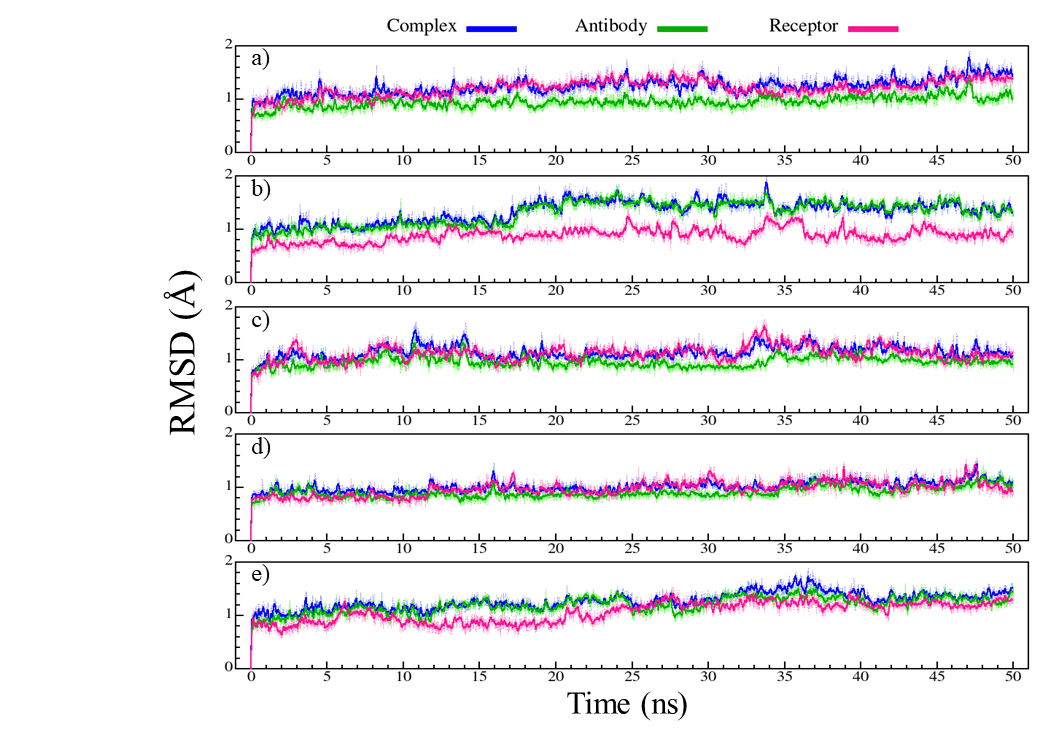


**S29 Fig. RMSD graphs for complex (blue), antibody (green), and PD-1 (magenta) during 50 ns of MD simulation.** (a) control-5xxy (atezolizumab-PD-L1). (b) design-0990. (c) design-1379. (d) design-4503. (e) design-9003.


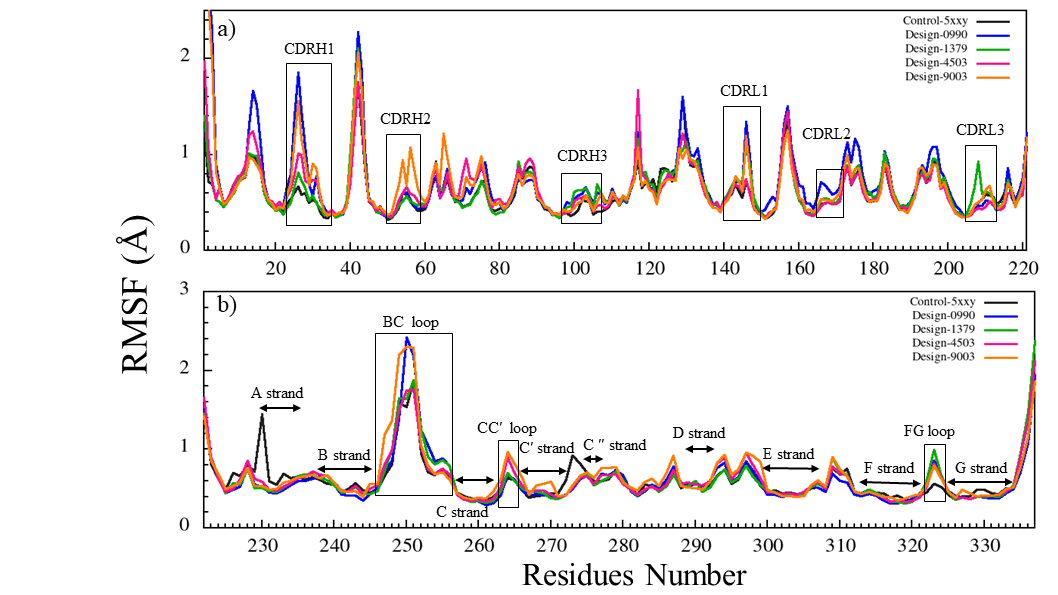


**S30 Fig. The RMSF graphs of PD-1 and antibodies during 50 ns of MD simulation.** RMSF graphs of PD-1 for control-5xxy (atezolizumab-PD-L1, dark gray), design-0990 (blue), design-1379 (green), design-4503 (magenta), and design-9003 (orange). (b) Comparison of RMSF graphs of PD-1 when bound to antibodies.


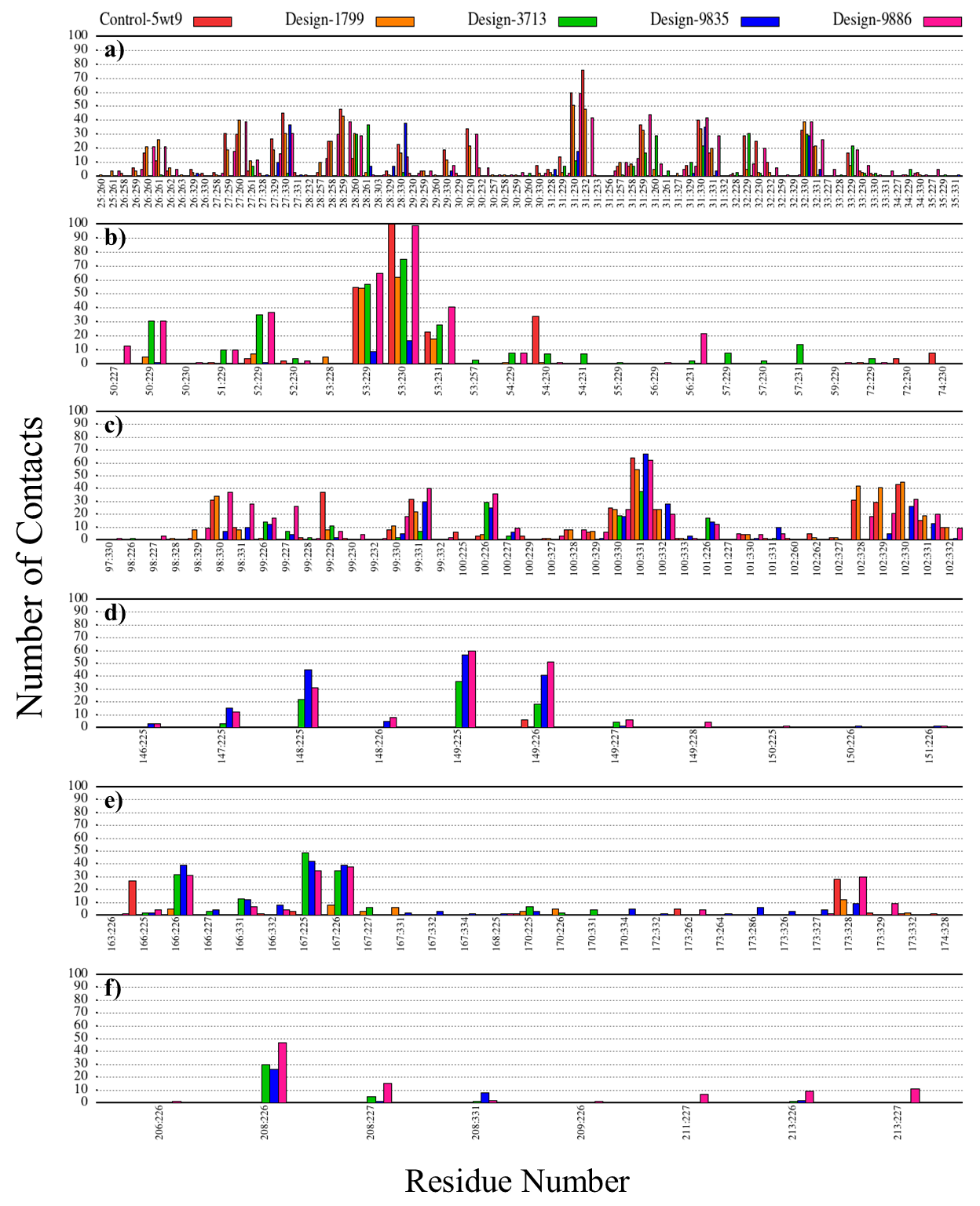


**S31 Fig. Contact patterns of complexes during 50 ns of MD simulation.** Number of contacts for residues in (a) CDRH1, (b) CDRH2, (c) CDRH3, (d) CDRL1, (e) CDRL2, and (f) CDRL3. Control-5wt9 (nivolumab-PD-1, red), design-1799-PD-1 (orange), design-3713-PD-1 (green), design-9835-PD-1 (blue), and design-9886-PD-1 (magenta). The x-axis shows pairwise residue interactions of the antibody- receptor complex.


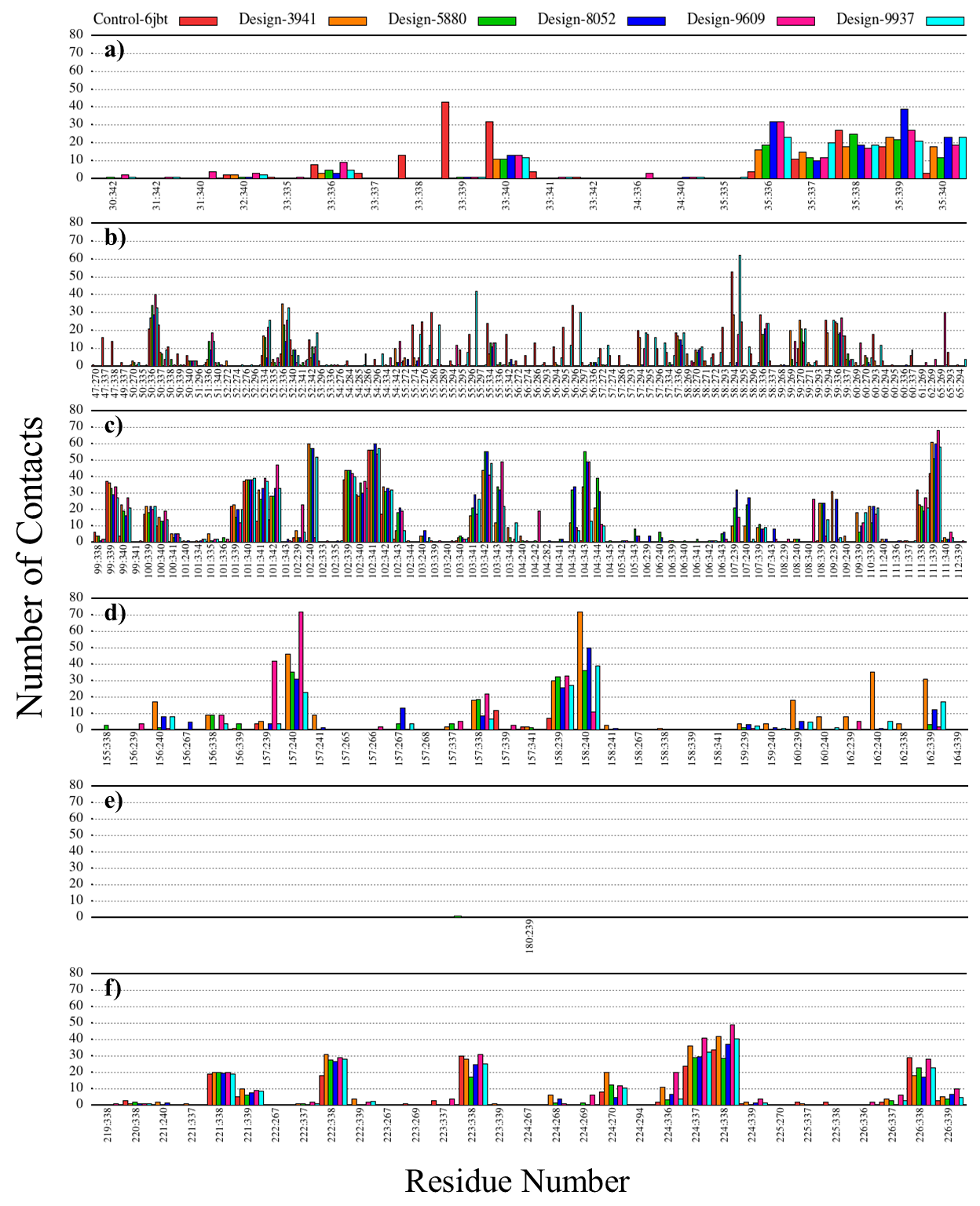


**S32 Fig. Contact patterns of complexes during 50 ns of MD simulation.** Number of contacts for residues in (a) CDRH1, (b) CDRH2, (c) CDRH3, (d) CDRL1, (e) CDRL2, and (f) CDRL3. Control-6jbt (toripalimab-PD-1, red), design-3941-PD-1 (orange), design-5880-PD-1 (green), design-8052-PD-1 (blue), design-9609-PD-1 (magenta), and design-9937-PD-1 (cyan). The x-axis shows pairwise residue interactions of the antibody- receptor complex.


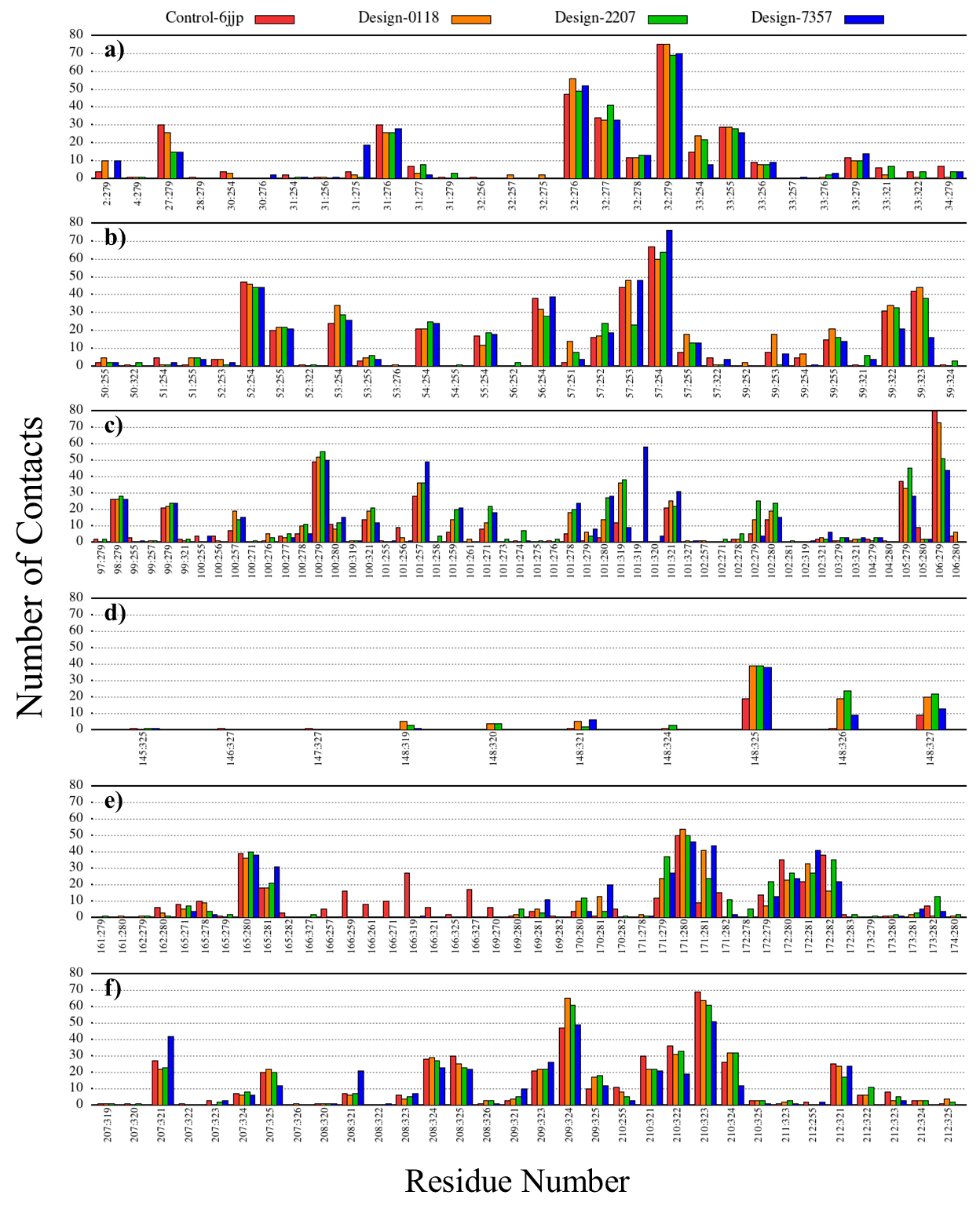


**S33 Fig. Contact patterns of complexes during 50 ns of MD simulation.** Number of contacts for residues in (a) CDRH1, (b) CDRH2, (c) CDRH3, (d) CDRL1, (e) CDRL2, and (f) CDRL3. Control-6jjp (MW11-h317-PD-1, red), design-0118-PD-1 (orange), design-2207-PD-1 (green), and design-7357-PD-1 (blue). The X-axis shows residue-residue contacts of the antibody- receptor. The x-axis shows pairwise residue interactions of the antibody- receptor complex.


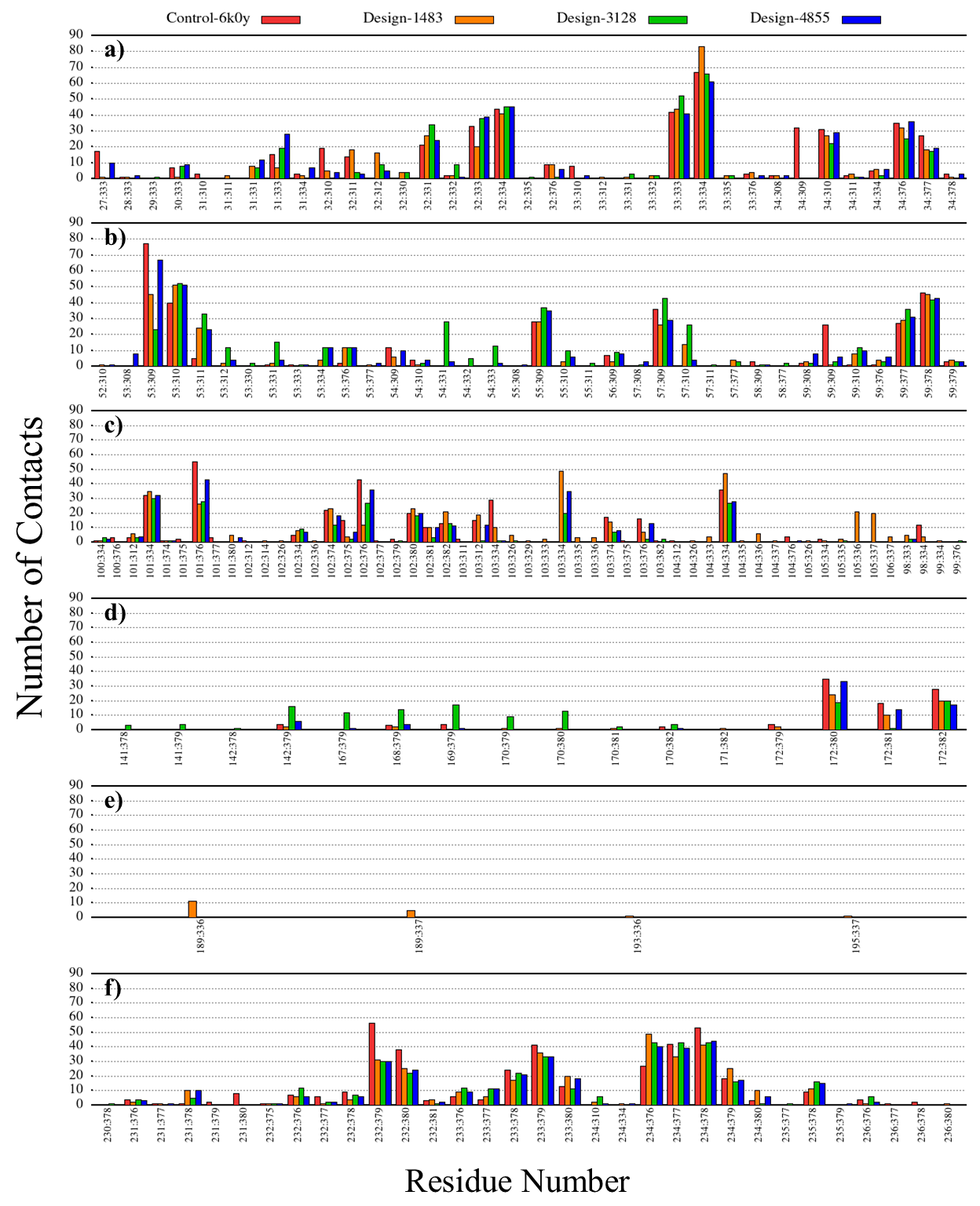


**S34 Fig. Contact patterns of complexes during 50 ns of MD simulation.** Number of contacts for residues in (a) CDRH1, (b) CDRH2, (c) CDRH3, (d) CDRL1, (e) CDRL2, and (f) CDRL3. Control-6k0y (mAb059c-PD-1, red), design-1483-PD-1 (orange), design-3128-PD-1 (green), and design-4855-PD-1 (blue). The x-axis shows pairwise residue interactions of the antibody- receptor complex.


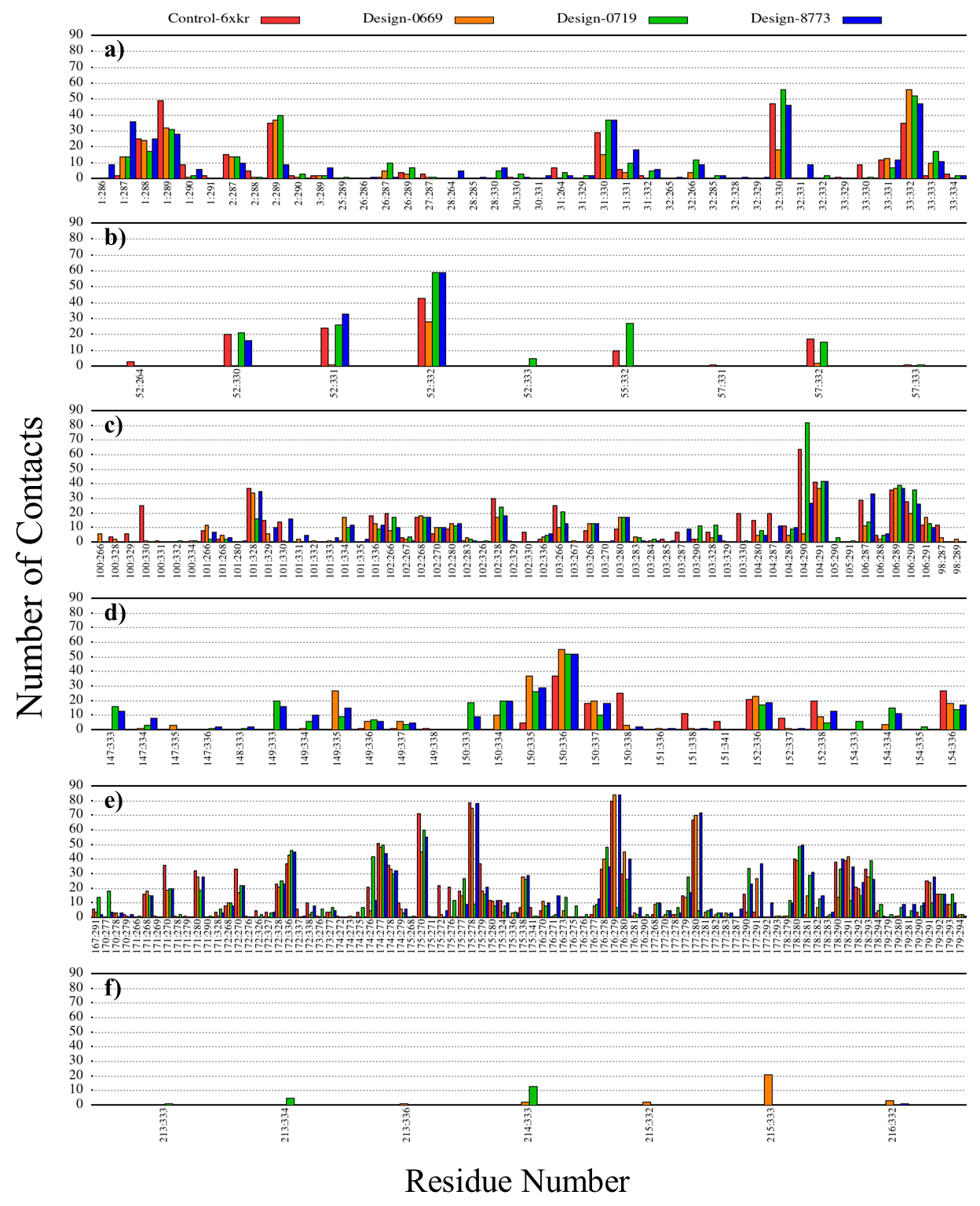


**S35 Fig. Contact patterns of complexes during 50 ns of MD simulation.** Number of contacts for residues in (a) CDRH1, (b) CDRH2, (c) CDRH3, (d) CDRL1, (e) CDRL2, and (f) CDRL3. Control-6xkr (sasanlimab-PD-1, red), design-0619-PD-1 (orange), design-0719-PD-1 (green), and design-8773-PD-1 (blue). The x-axis shows pairwise residue interactions of the antibody- receptor complex.


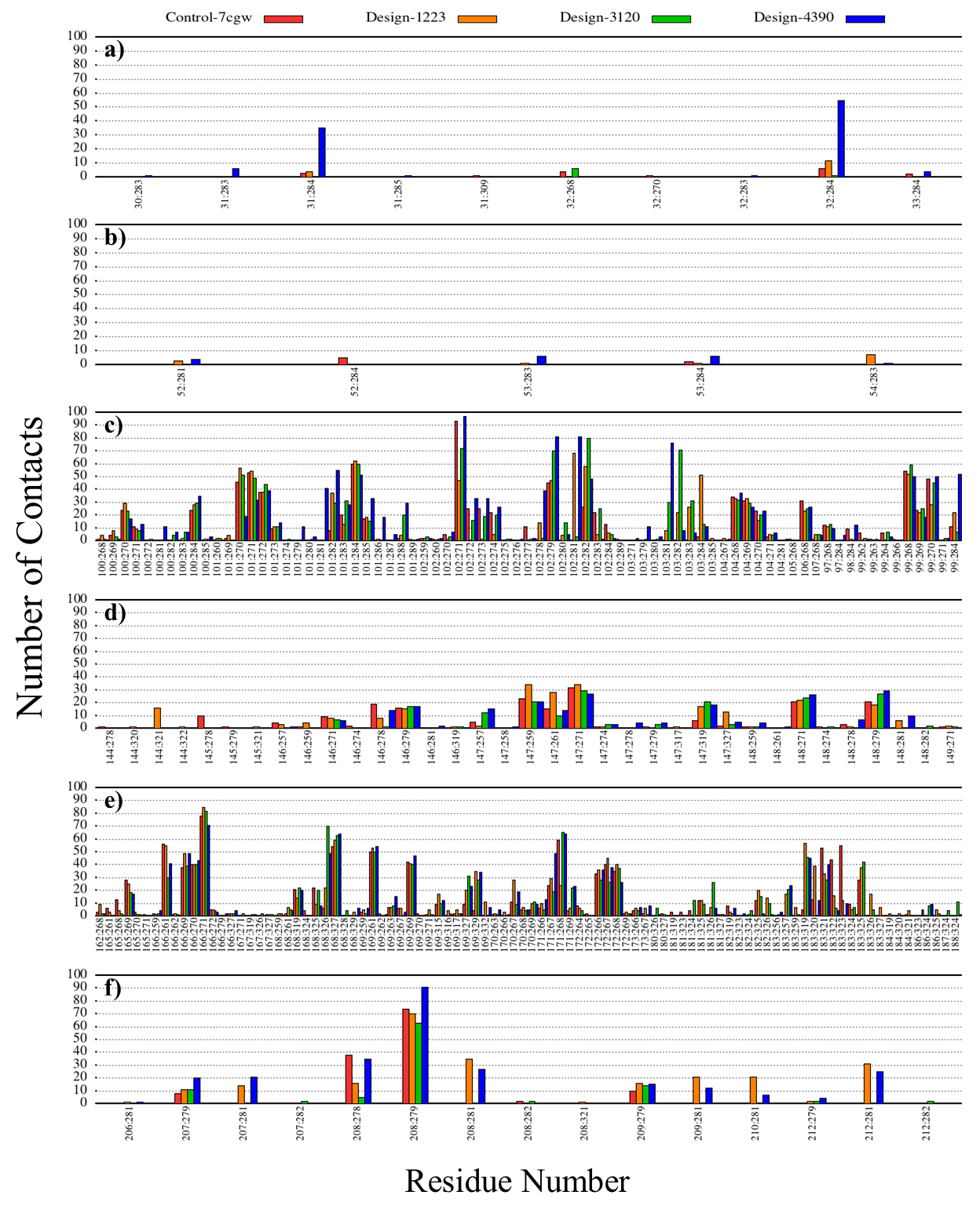


**S36 Fig. Contact patterns of complexes during 50 ns of MD simulation.** Number of contacts for residues in (a) CDRH1, (b) CDRH2, (c) CDRH3, (d) CDRL1, (e) CDRL2, and (f) CDRL3. Control-7cgw (tislelizumab-PD-1, red), design-1223-PD-1 (orange), design-3120-PD-1 (green), and design-4390-PD-1 (blue). The x-axis shows pairwise residue interactions of the antibody- receptor complex.


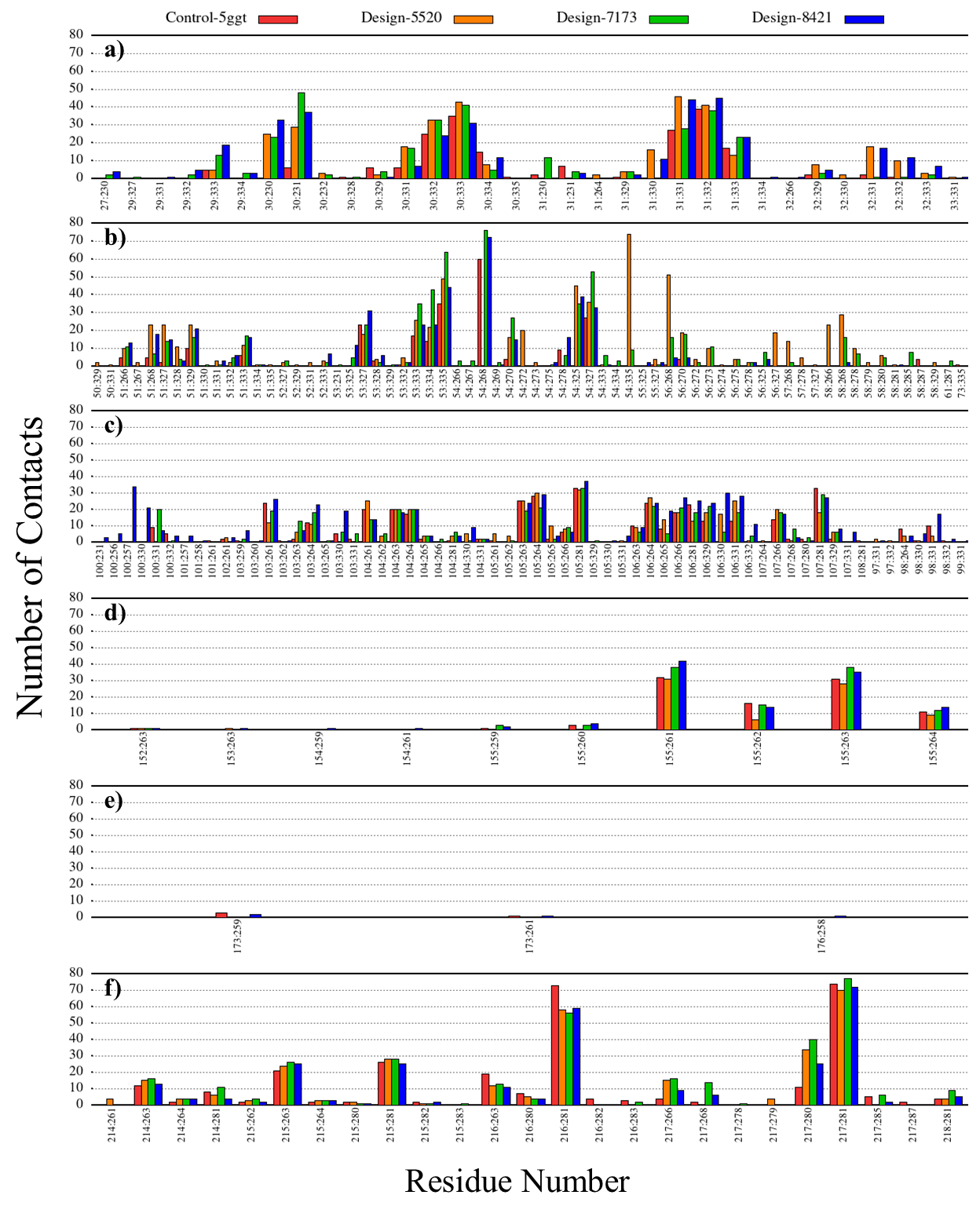


**S37 Fig. Contact patterns of complexes during 50 ns of MD simulation.** Number of contacts for residues in (a) CDRH1, (b) CDRH2, (c) CDRH3, (d) CDRL1, (e) CDRL2, and (f) CDRL3. Control-5ggt (BMS-936559-PD-L1, red), design-5520-PD-L1 (orange), design-7173-PD-L1 (green), and design-8421-PD-L1 (blue). The x-axis shows pairwise residue interactions of the antibody- receptor complex.


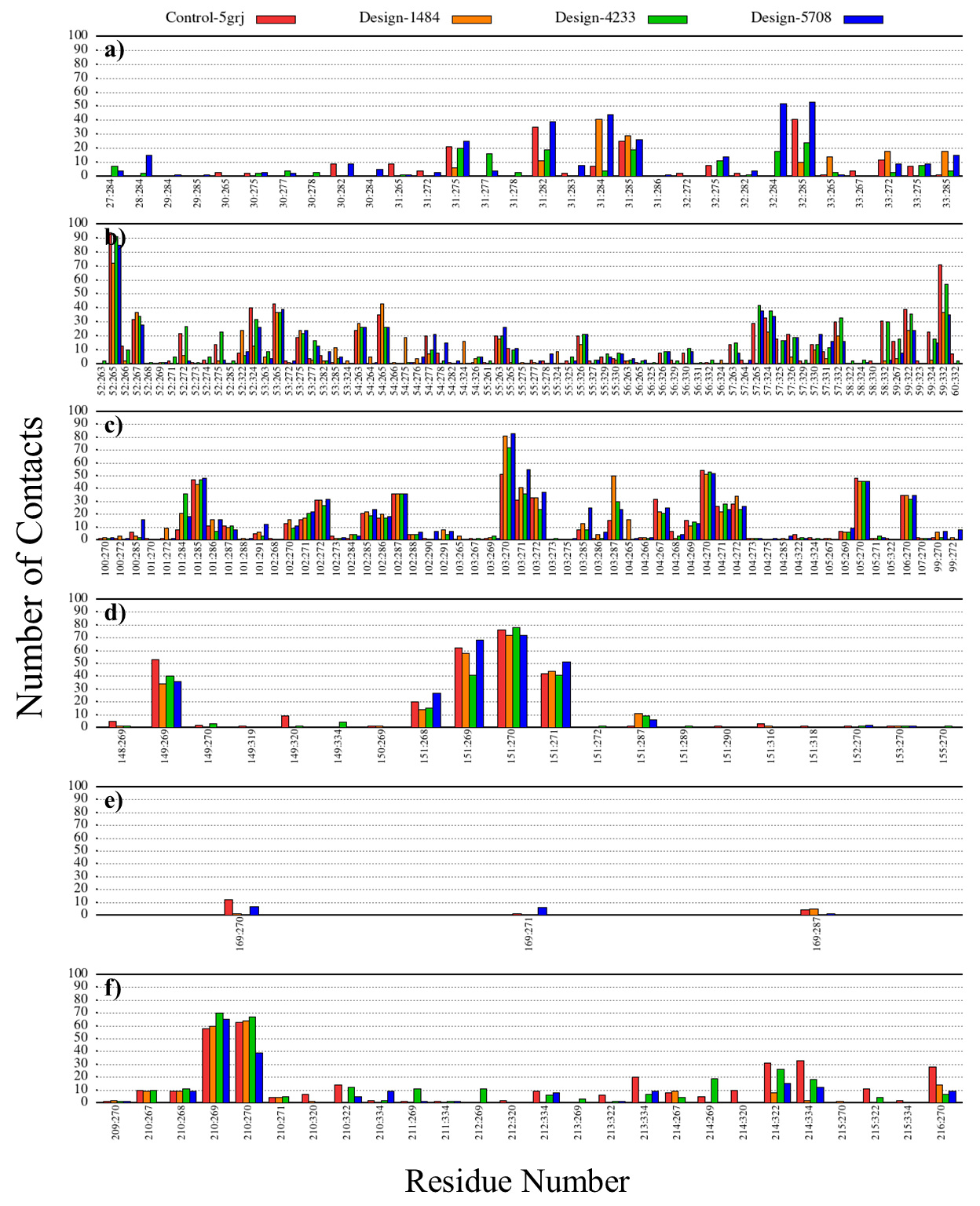


**S38 Fig. Contact patterns of complexes during 50 ns of MD simulation.** Number of contacts for residues in (a) CDRH1, (b) CDRH2, (c) CDRH3, (d) CDRL1, (e) CDRL2, and (f) CDRL3. Control-5jrg (avelumab-PD-L1, red), design-1484-PD-L1 (orange), design-4233-PD-L1 (green), and design-5708-PD-L1 (blue). The x-axis shows pairwise residue interactions of the antibody- receptor complex.


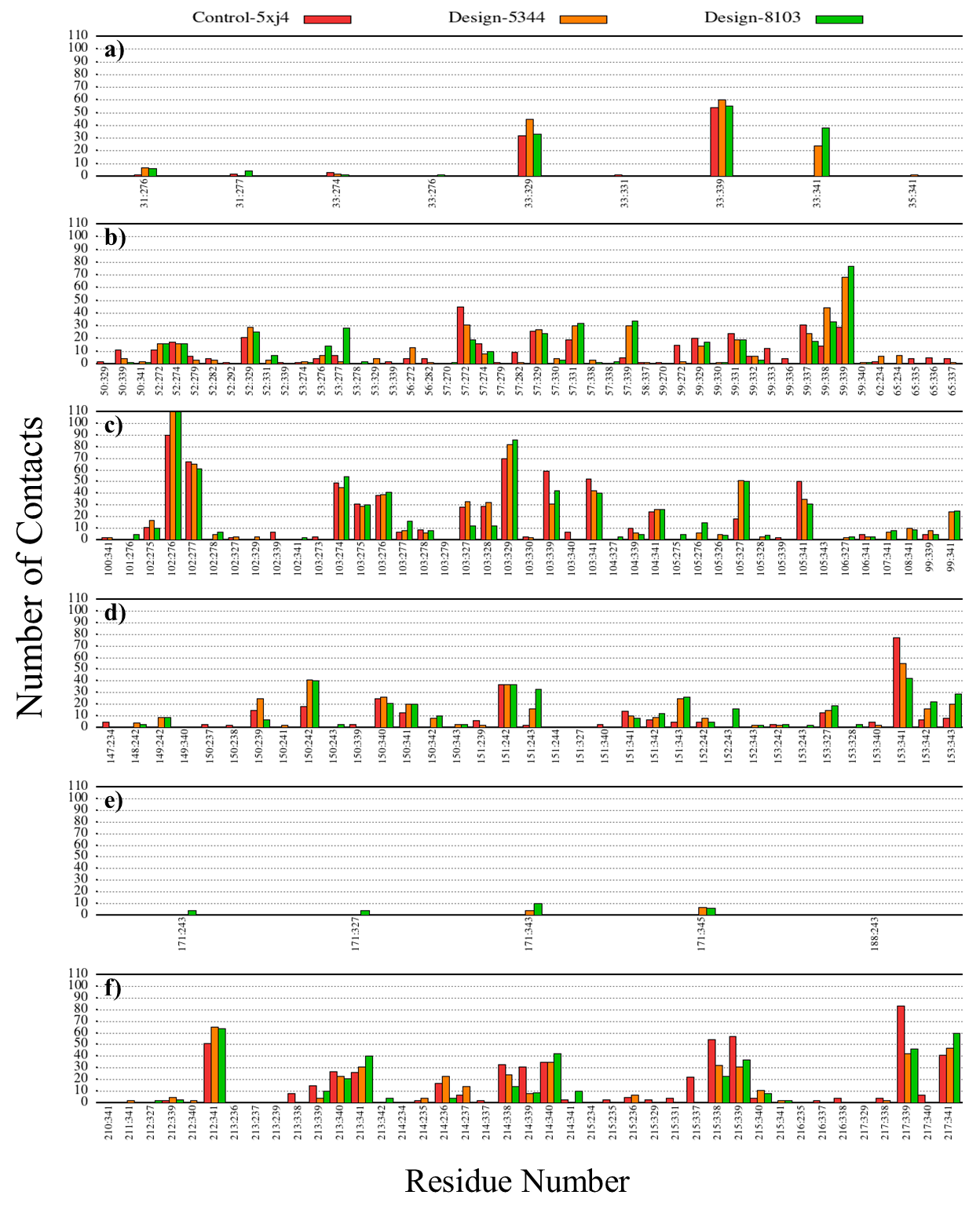


**S39 Fig. Contact patterns of complexes during 50 ns of MD simulation.** Number of contacts for residues in (a) CDRH1, (b) CDRH2, (c) CDRH3, (d) CDRL1, (e) CDRL2, and (f) CDRL3. Control-5xj4 (durvalumab-PD-L1, red), design-5344-PD-L1 (orange), and design-8103-PD-L1 (green). The x-axis shows pairwise residue interactions of the antibody- receptor complex.


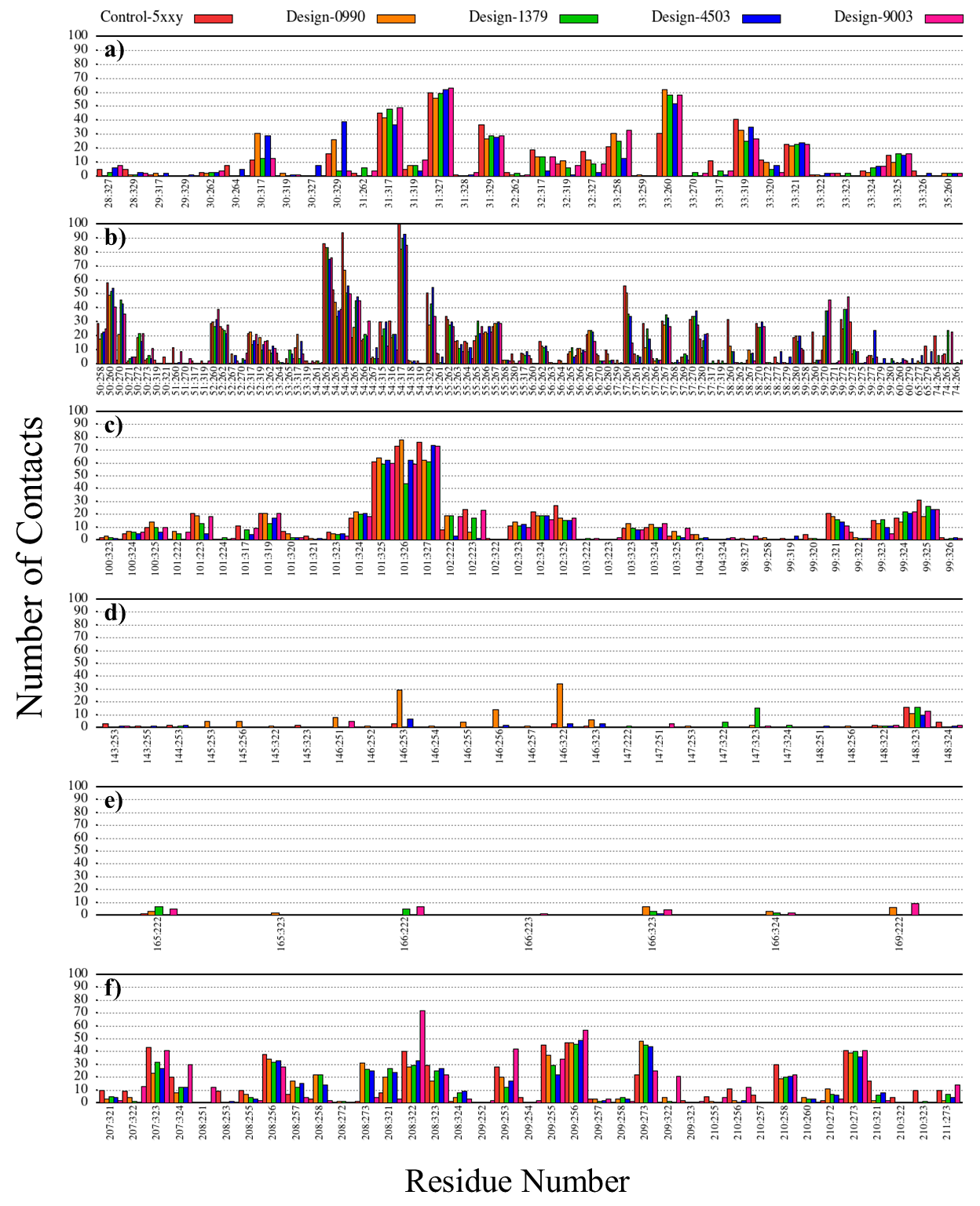


**S40 Fig. Contact patterns of complexes during 50 ns of MD simulation.** Number of contacts for residues in (a) CDRH1, (b) CDRH2, (c) CDRH3, (d) CDRL1, (e) CDRL2, and (f) CDRL3. Control-5xxy (atezolizumab-PD-L1, red), design-0990-PD-L1 (orange), design-1379-PD-L1 (green), design-4503-PD-L1 (blue), and design-9003-PD-L1 (magenta). The x-axis shows pairwise residue interactions of the antibody- receptor complex.


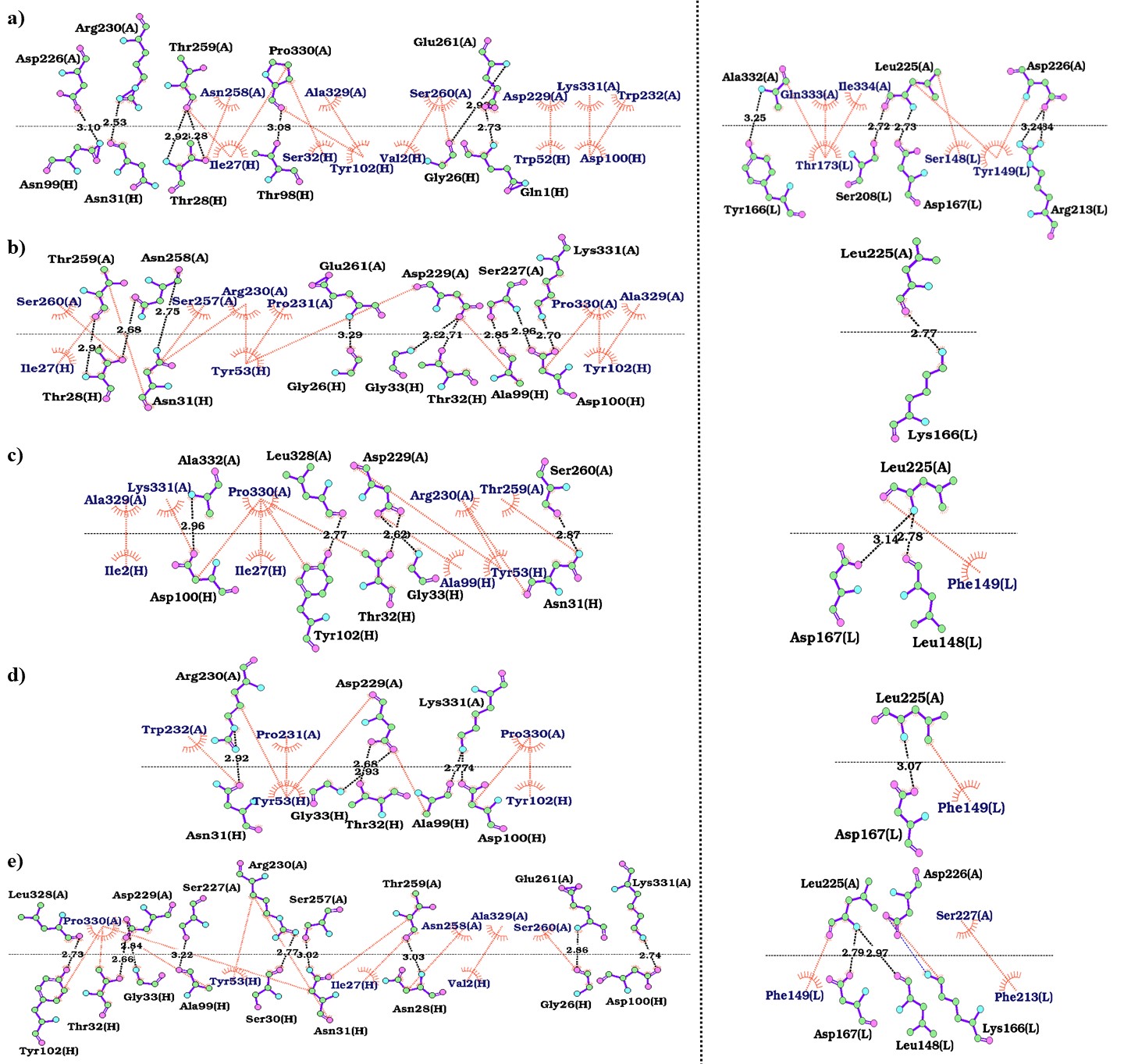


**S41 Fig. 2D interaction maps of heavy (left) and light (right) chains of antibodies in complex with PD-1.** The interactions between the antibody chains and PD-1 in (a) control-5wt9 (nivolumab-PD-1), (b) design-1799, (c) design-3713, (d) design-9835, (e) and design-9886 were analyzed using LigPlot^+^. The hydrogen bonds, hydrophobic interactions and salt-bridges are colored in orange, black and blue lines, respectively. The PD-1 and light and heavy chains of the antibody are labeled A, H, and L, respectively.


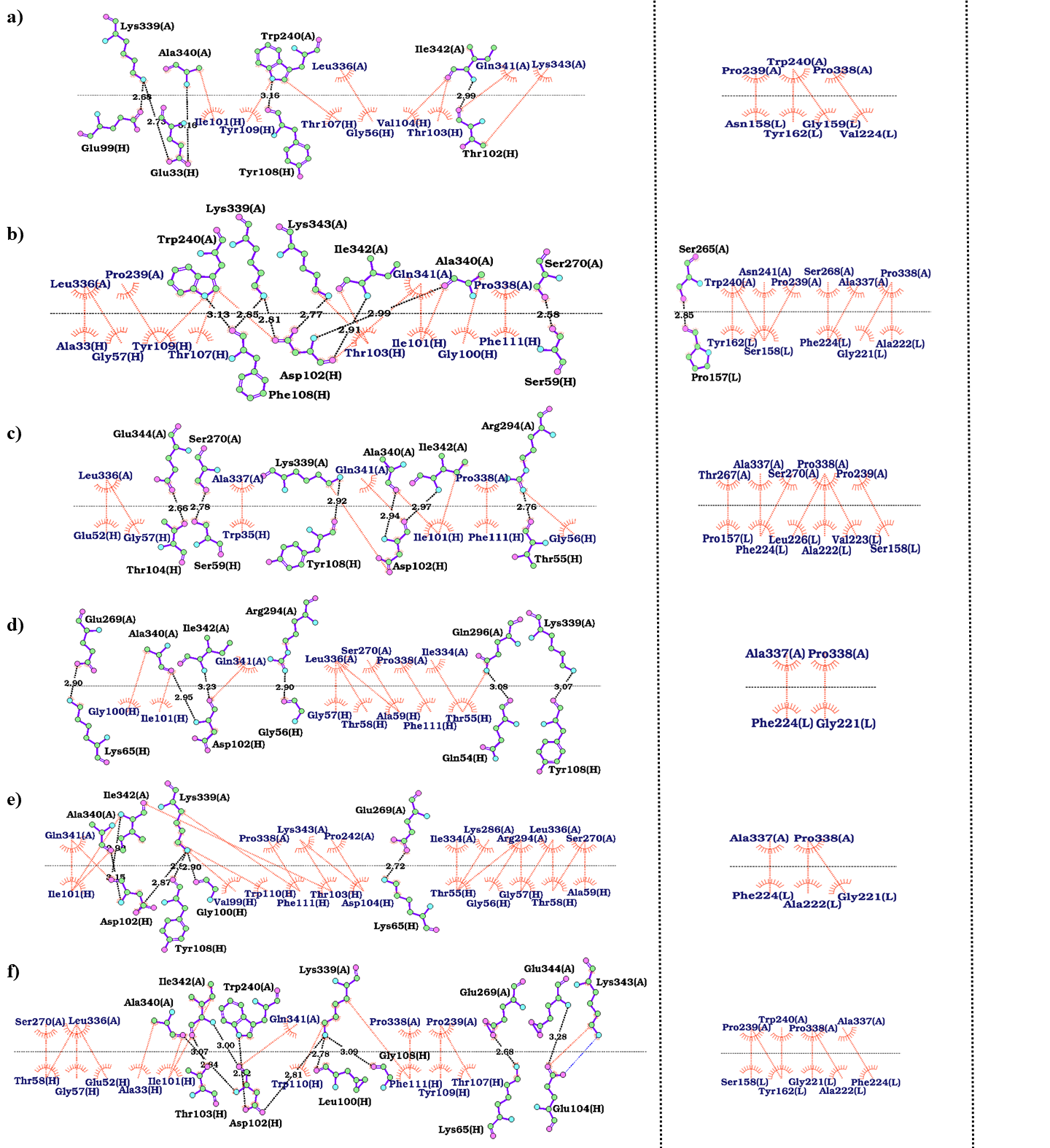


**S42 Fig. 2D interaction maps of heavy (left) and light (right) chains of antibodies in complex with PD-1.** The interactions between the antibody chains and PD-1 in (a) control-6jbt (toripalimab-PD-1), (b) design-3941, (c) design-5880, (d) design-8052, (e) and design-9609, (f) and design-9937 were analyzed using LigPlot^+^. The hydrogen bonds, hydrophobic interactions and salt-bridges are colored in orange, black and blue lines, respectively. The PD-1 and light and heavy chains of the antibody are labeled A, H, and L, respectively.


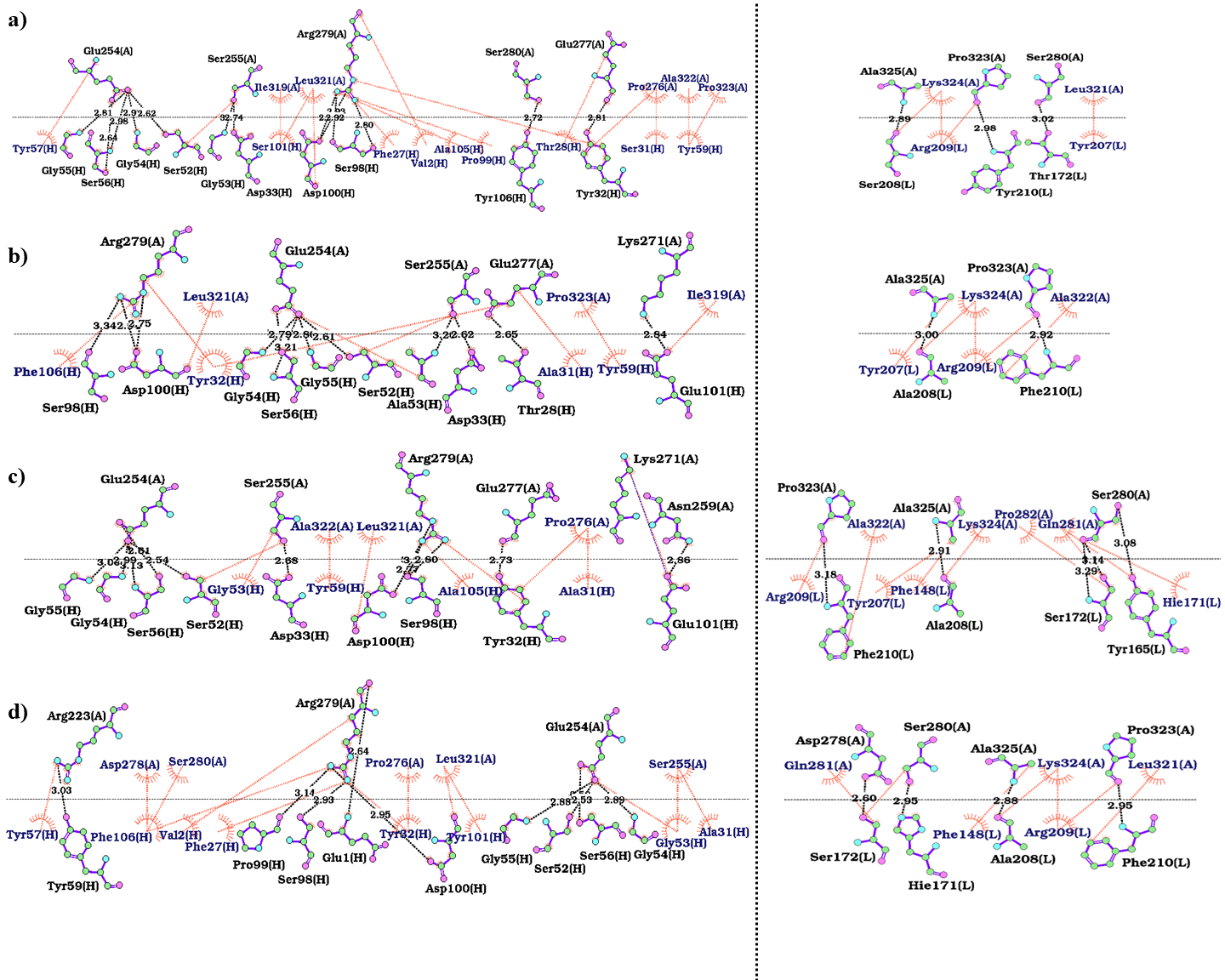


**S43 Fig. 2D interaction maps of heavy (left) and light (right) chains of antibodies in complex with PD-1.** The interactions between the antibody chains and PD-1 in (a) control-6jjp (MW11-h317-PD-1), (b) design-0118, (c) design-2207, and (d) design-7357 were analyzed using LigPlot^+^. The hydrogen bonds and hydrophobic interactions are colored in orange and black, respectively. The PD-1 and light and heavy chains of the antibody are labeled A, H, and L, respectively.


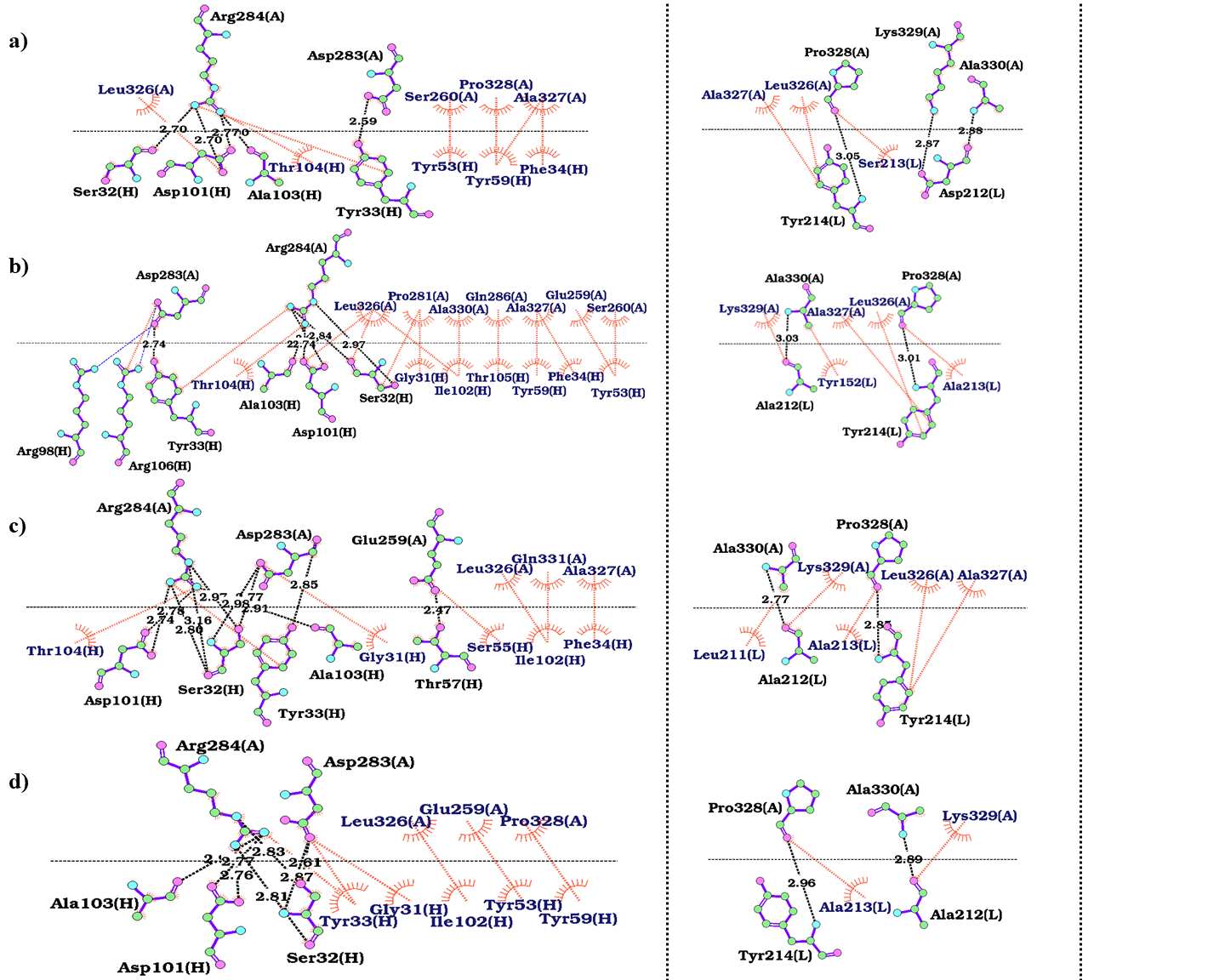


**S44 Fig. 2D interaction maps of heavy (left) and light (right) chains of antibodies in complex with PD-1.** The interactions between the antibody chains and PD-1 in (a) control-6k0y (mAb059c-PD-1), (b) design-1483, (c) design-3128, and (d) design-4855 were analyzed using LigPlot^+^. The hydrogen bonds, hydrophobic interactions and salt-bridges are colored in orange, black and blue lines, respectively. The PD-1 and light and heavy chains of the antibody are labeled A, H, and L, respectively.


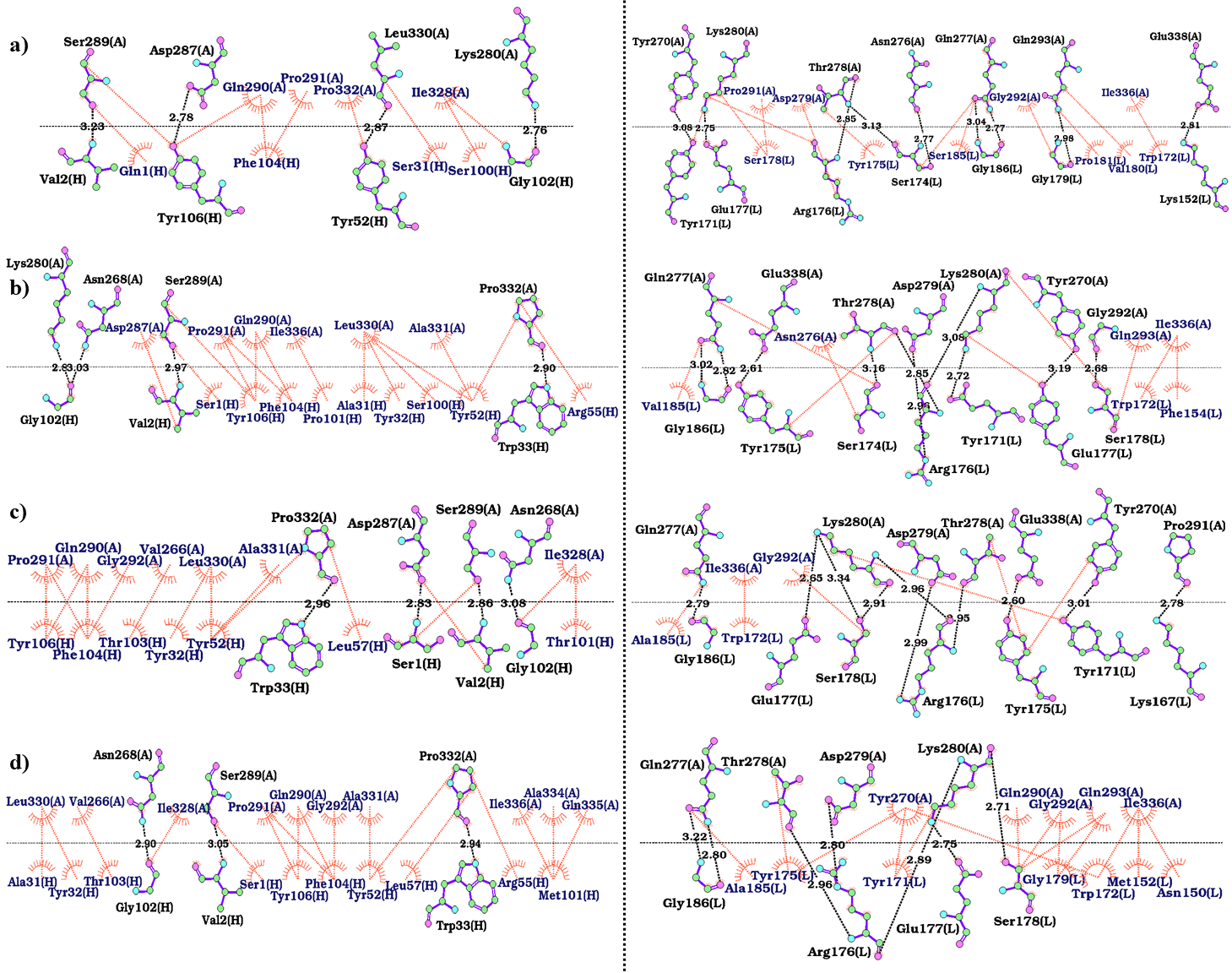


**S45 Fig. 2D interaction maps of heavy (left) and light (right) chains of antibodies in complex with PD-1.** The interactions between the antibody chains and PD-1 in (a) control-6xkr (sasanlimab-PD-1), (b) design-0669, (c) design-0719, and (d) design-8773 were analyzed using LigPlot^+^. The hydrogen bonds and hydrophobic interactions are colored in orange and black lines, respectively. The PD-1 and light and heavy chains of the antibody are labeled A, H, and L, respectively.


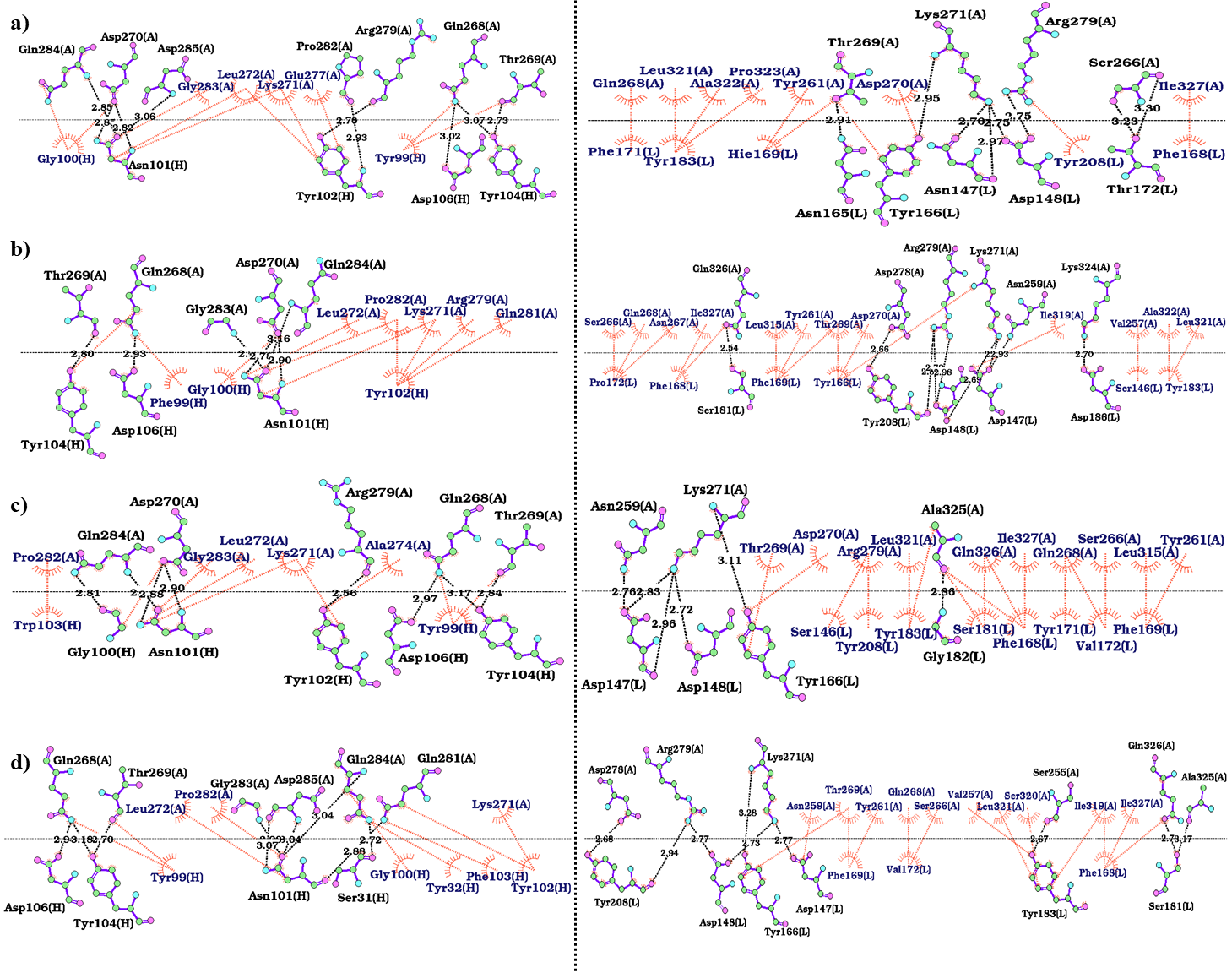


**S46 Fig. 2D interaction maps of heavy (left) and light (right) chains of antibodies in complex with PD-1.** The interactions between the antibody chains and PD-1 in (a) control-7cgw (tislelizumab-PD-1), (b) design-1223, (c) design-3120, and (d) design-4390 were analyzed using LigPlot^+^. The hydrogen bonds and hydrophobic interactions are colored in orange and black lines, respectively. The PD-1 and light and heavy chains of the antibody are labeled A, H, and L, respectively.


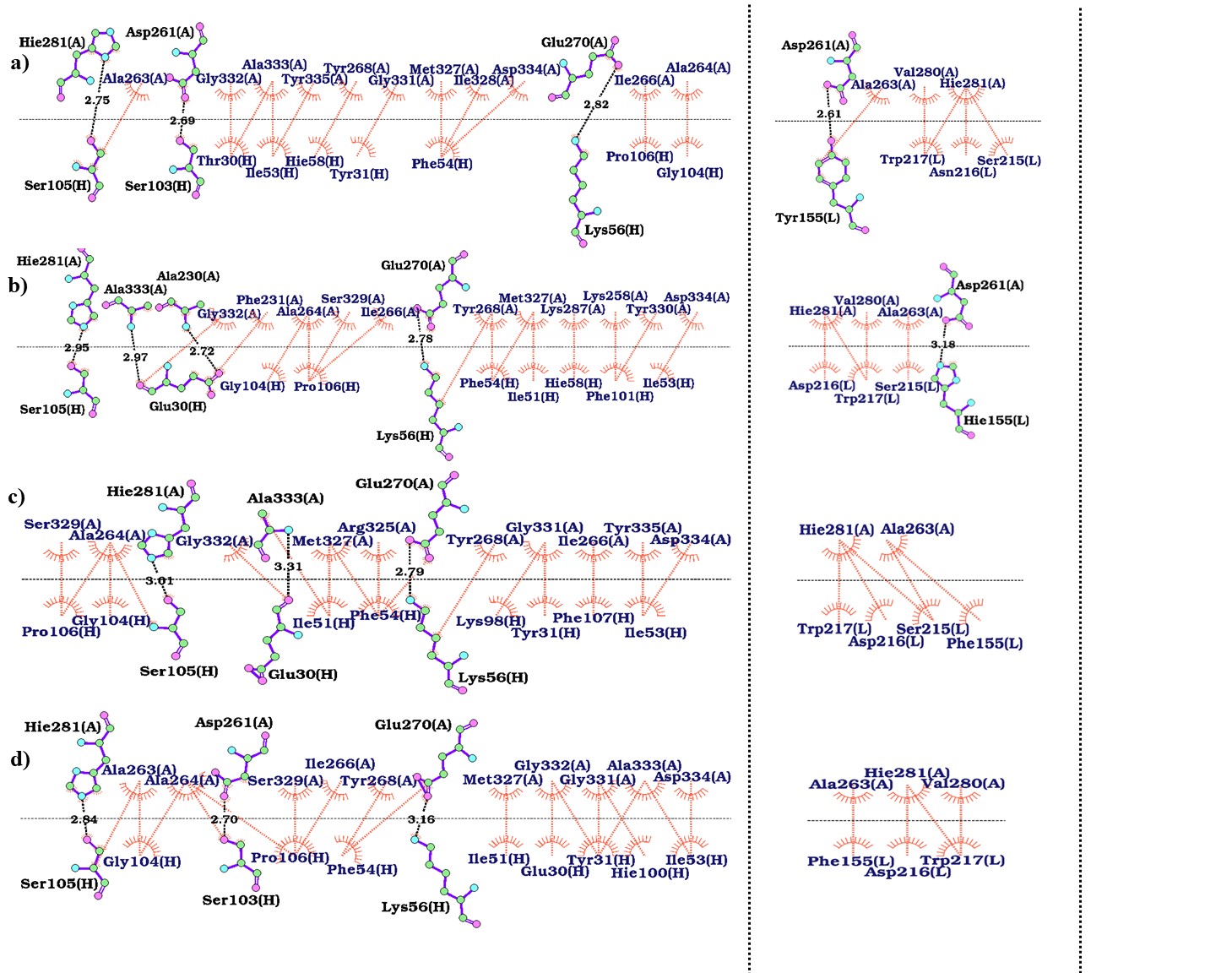


**S47 Fig. 2D interaction maps of heavy (left) and light (right) chains of antibodies in complex with PD-L1.** The interactions between the antibody chains and PD-1 in (a) control-5ggt (BMS-936559-PD-1), (b) design-5520, (c) design-7173, and (d) design-8421 were analyzed using LigPlot^+^. The hydrogen bonds and hydrophobic interactions are colored in orange and black lines, respectively. The PD-1 and light and heavy chains of the antibody are labeled A, H, and L, respectively.


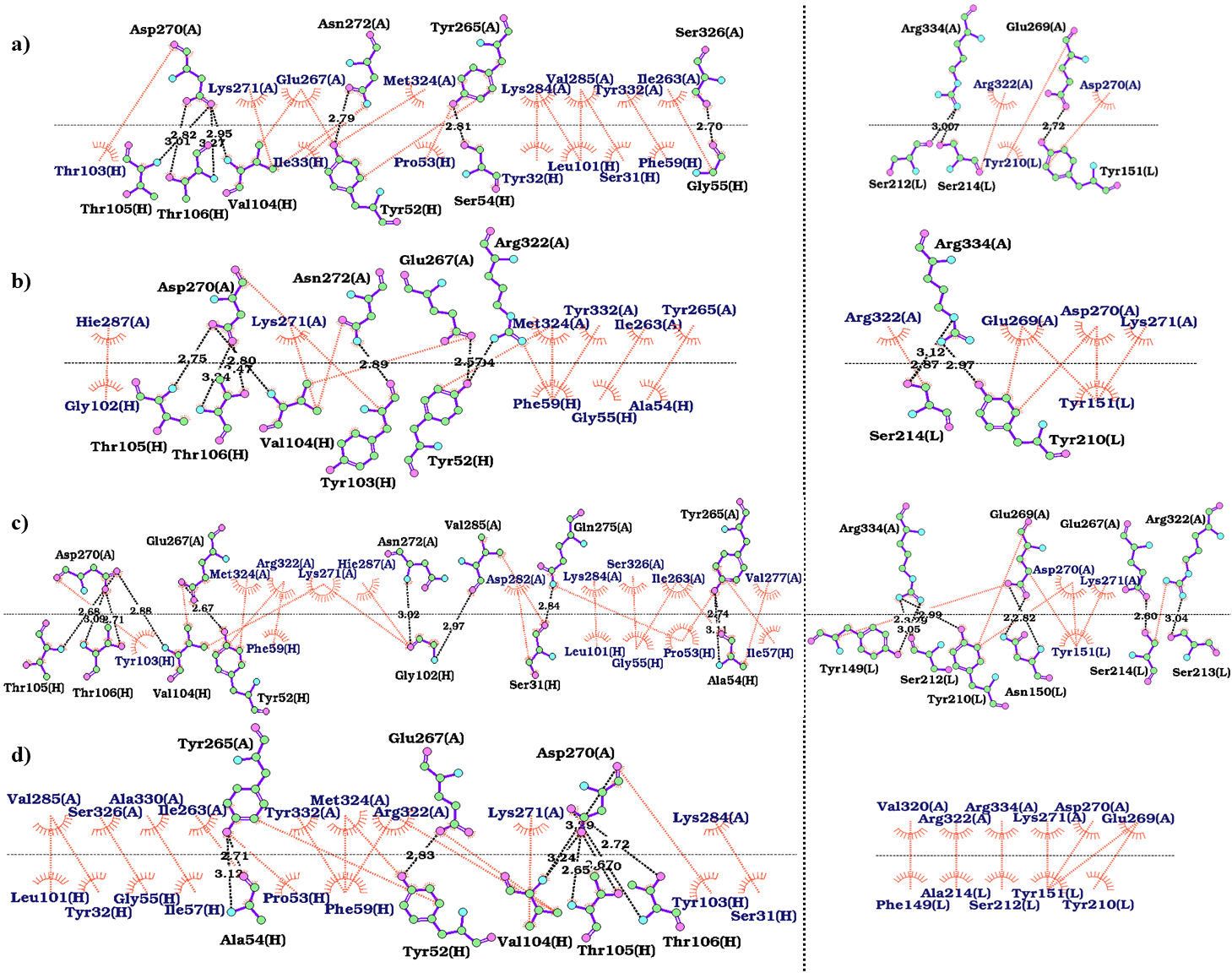


**S48 Fig. 2D interaction maps of heavy (left) and light (right) chains of antibodies in complex with PD-L1.** The interactions between the antibody chains and PD-1 in (a) control-5grj (avelumab-PD-L1), (b) design-1484, (c) design-4233, and (d) design-5708 were analyzed using LigPlot^+^. The hydrogen bonds and hydrophobic interactions are colored in orange and black lines, respectively. The PD-1 and light and heavy chains of the antibody are labeled A, H, and L, respectively.


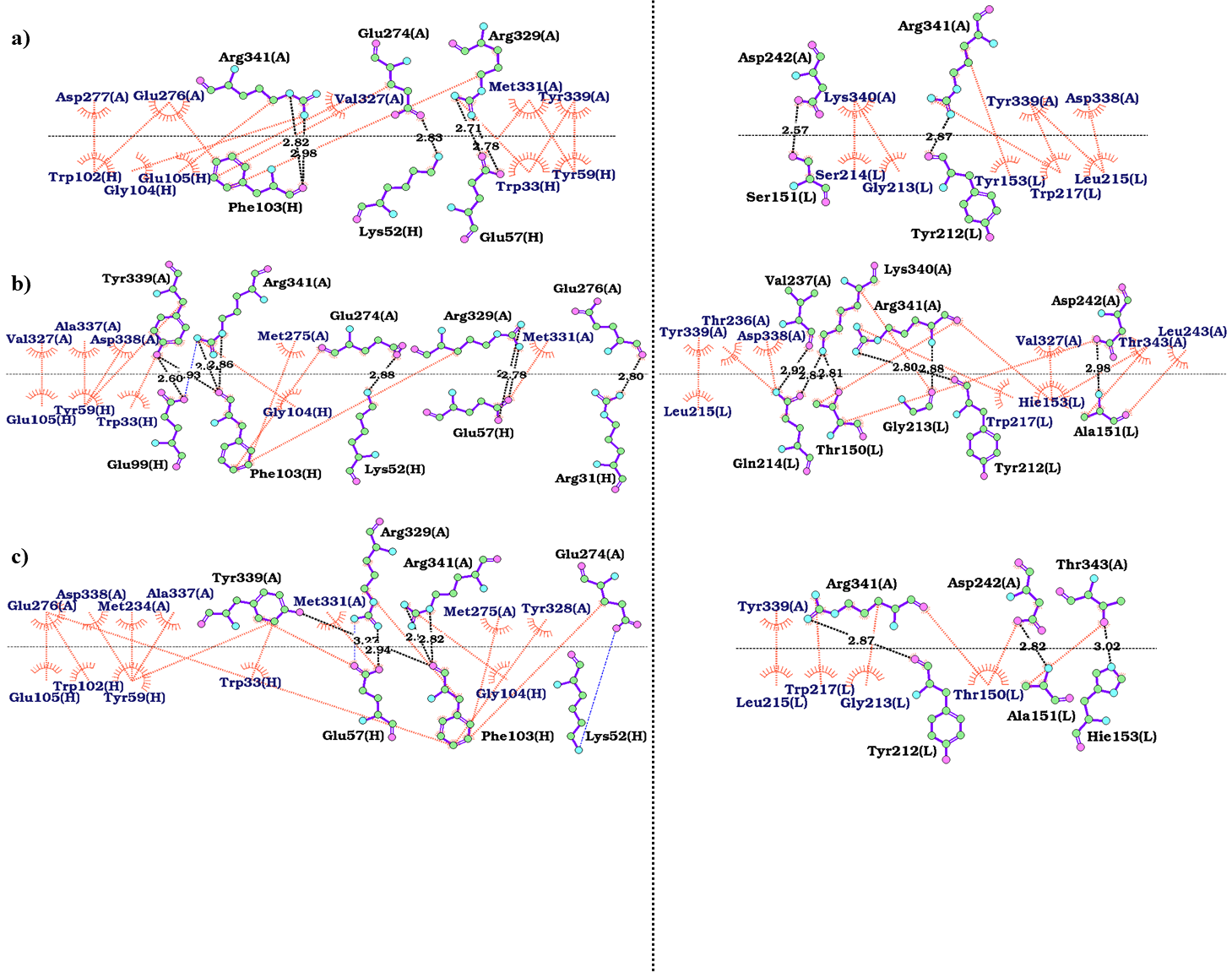


**S49 Fig. 2D interaction maps of heavy (left) and light (right) chains of antibodies in complex with PD-L1.** The interactions between the antibody chains and PD-1 in (a) control-5xj4 (durvalumab-PD-L1), (b) design-5344, and (c) design-8103 were analyzed using LigPlot^+^. The hydrogen bond, hydrophobic, and salt-bridge interactions are colored by the tomato, black, and blue lines, respectively. The PD-1 and light and heavy chains of the antibody are labeled A, H, and L, respectively.


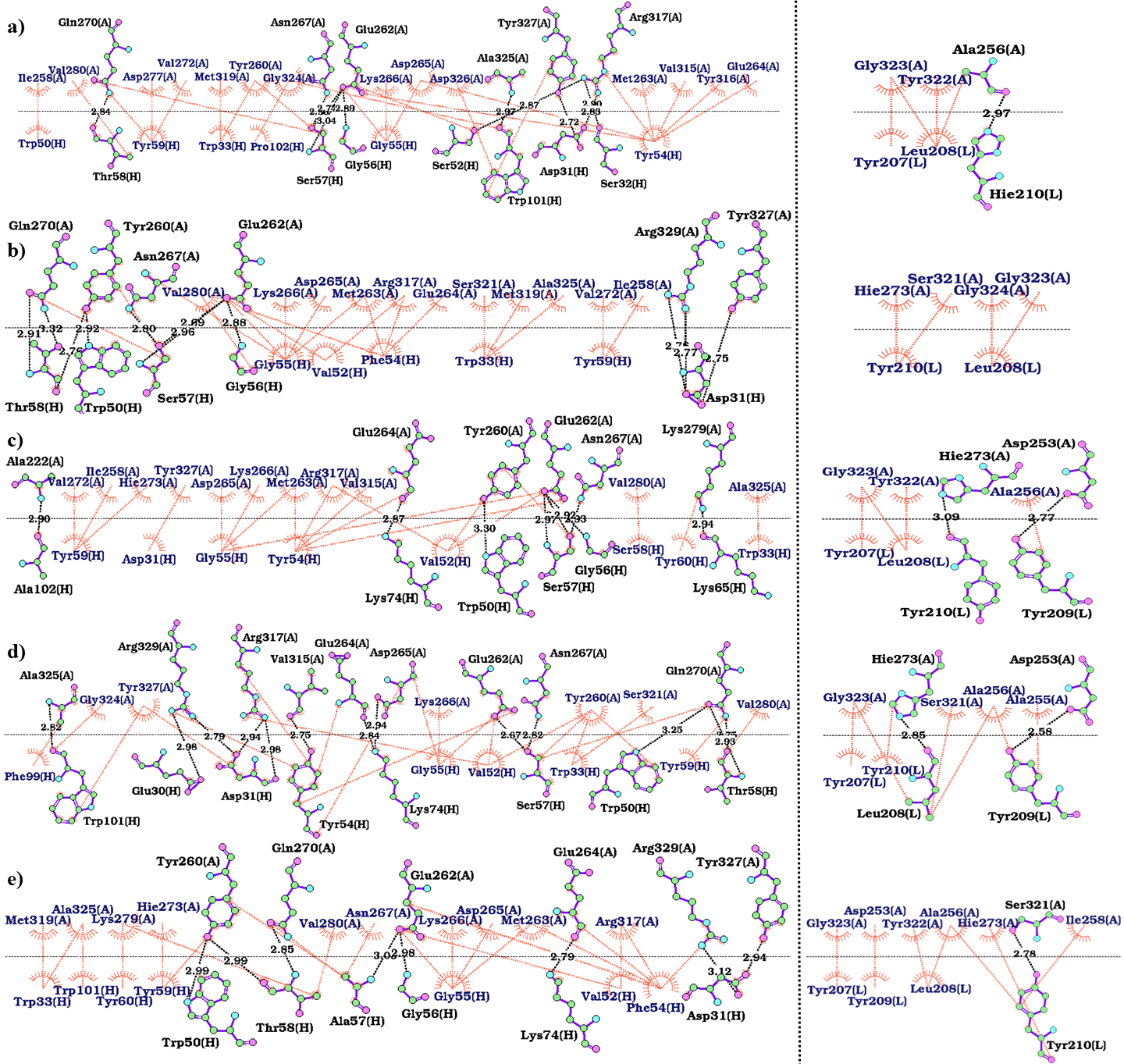


**S50 Fig. 2D interaction maps of heavy (left) and light (right) chains of antibodies in complex with PD-L1.** The interactions between the antibody chains and PD-1 in (a) control-5xxy (atezolizumab-PD-L1), (b) design-0990, (c) design-1379, (d) design-4503, and (e) design-9003 were analyzed using LigPlot^+^. The hydrogen bonds and hydrophobic interactions are colored in orange and black, respectively. The PD-1 and light and heavy chains of the antibody are labeled A, H, and L, respectively.


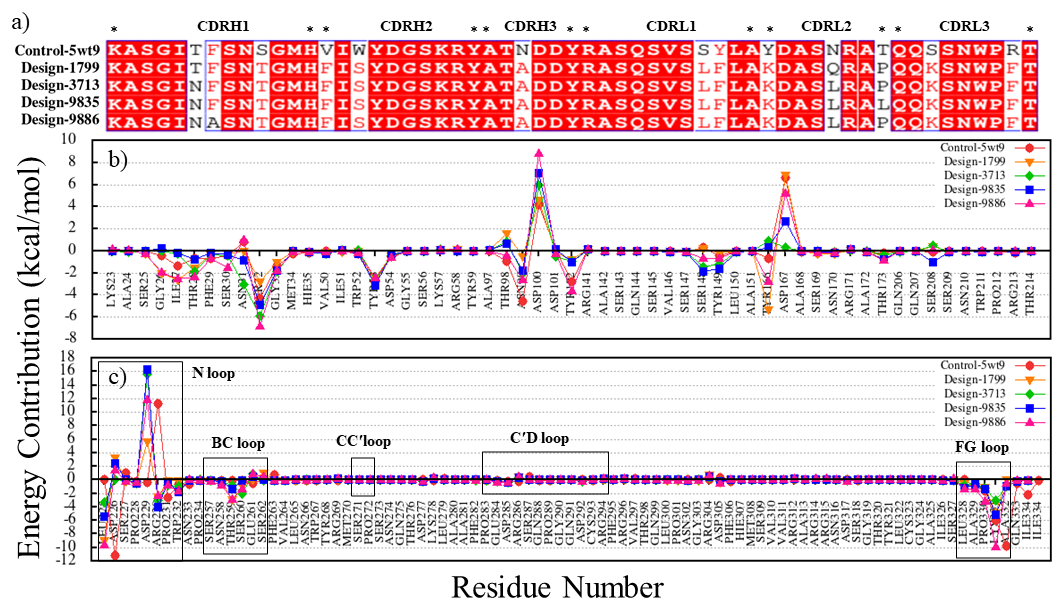


**S51 Fig. Binding free energy decomposition of the residues in the control and designed complexes.** (a) Alignment of CDR sequences in the control-5wt9 (nivolumab-PD-1) and design groups. (b) Energy contribution values of PD-1 residues from MM-PBSA. (c) Energy contribution values of CDR residues from MM-PBSA. The graphs show the binding free energy for each residue in control-5wt9 (red), design-1799 (orange), design-3713 (green), design-9835 (blue), and design-9886 (magenta).


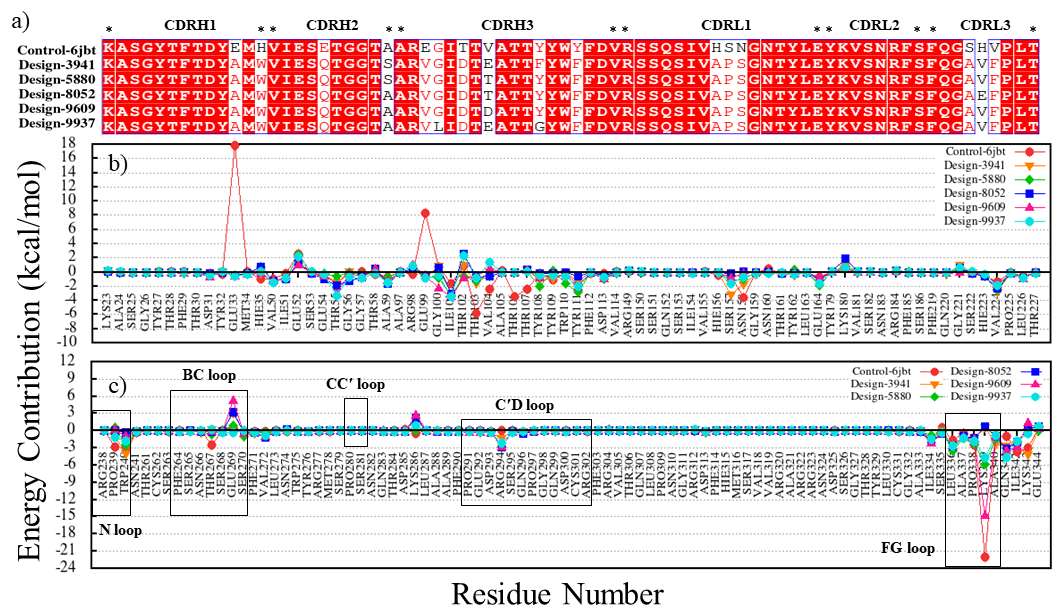


**S52 Fig. Binding free energy decomposition of the residues in the control and designed complexes.** (a) Alignment of CDR sequences in the control-6jbt (toripalimab-PD-1) and design groups. (b) Energy contribution values of PD-1 residues from MM-PBSA. (c) Energy contribution values of CDR residues from MM-PBSA. The graphs show the binding free energy for each residue in control-6jbt (red), design-3941 (orange), design-5880 (green), design-8052 (blue), design-9609 (magenta), and design-9937 (cyan).


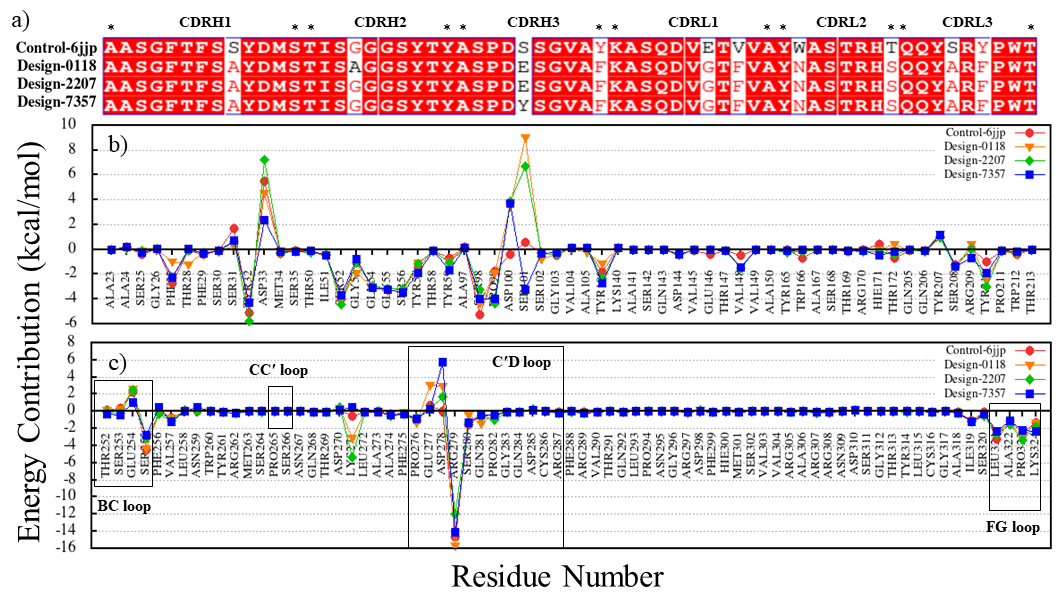


**S53 Fig. Binding free energy decomposition of the residues in the control and designed complexes.** (a) Alignment of CDR sequences in the control-6jjp (MW11-h317-PD-1) and design groups. (b) Energy contribution values of PD-1 residues from MM-PBSA. (c) Energy contribution values of CDR residues from MM-PBSA. The graphs show the binding free energy for each residue in control-6jjp (red), design-0118 (orange), design-2207 (green), and design-7357 (blue).


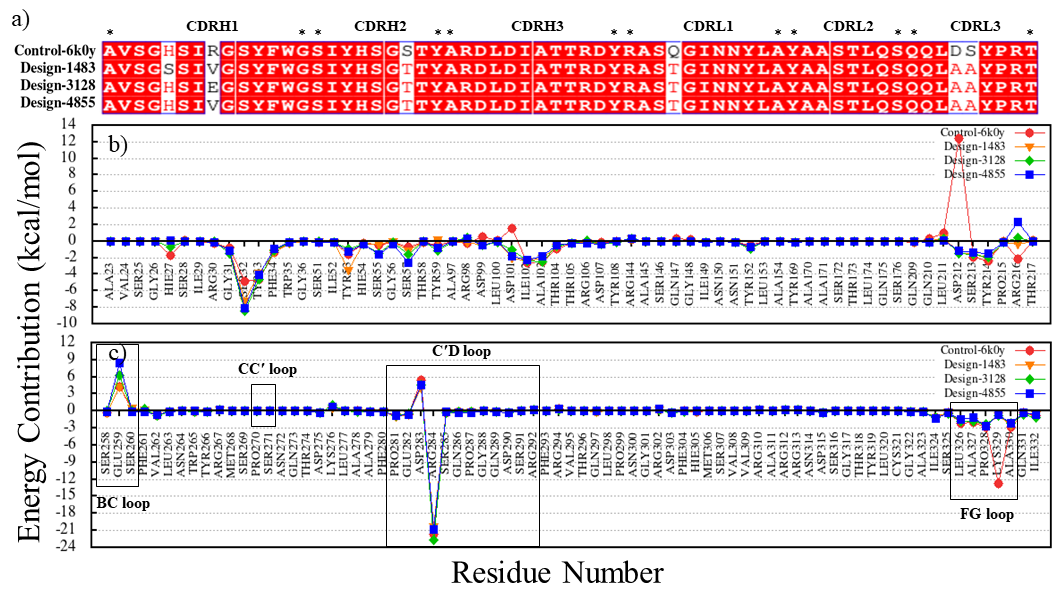


**S54 Fig. Binding free energy decomposition of the residues in the control and designed complexes.** (a) Alignment of CDR sequences in the control-6k0y (mAb059c-PD-1) and design groups. (b) Energy contribution values of PD-1 residues from MM-PBSA. (c) Energy contribution values of CDR residues from MM-PBSA. The graphs show the binding free energy for each residue in control-6k0y (red), design-1483 (orange), design-3128 (green), and design-4855 (blue).


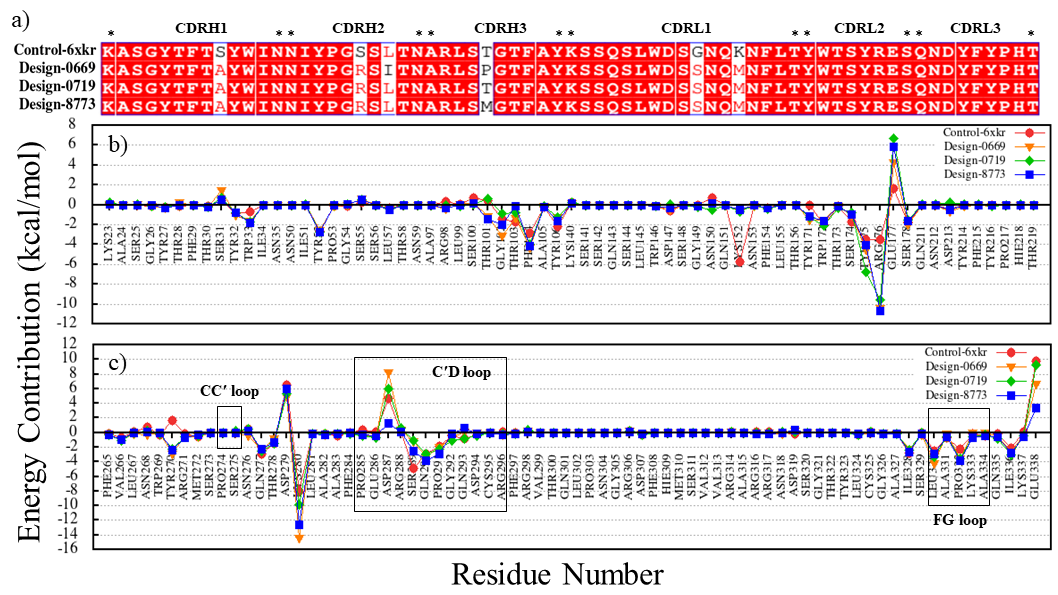


**S55 Fig. Binding free energy decomposition of the residues in the control and designed complexes.** (a) Alignment of CDR sequences in the control-6xkr (sasanlimab-PD-1) and design groups. (b) Energy contribution values of PD-1 residues from MM-PBSA. (c) Energy contribution values of CDR residues from MM-PBSA. The graphs show the binding free energy for each residue in control-6xkr (red), design-0669 (orange), design-0719 (green), and design-8773 (blue).


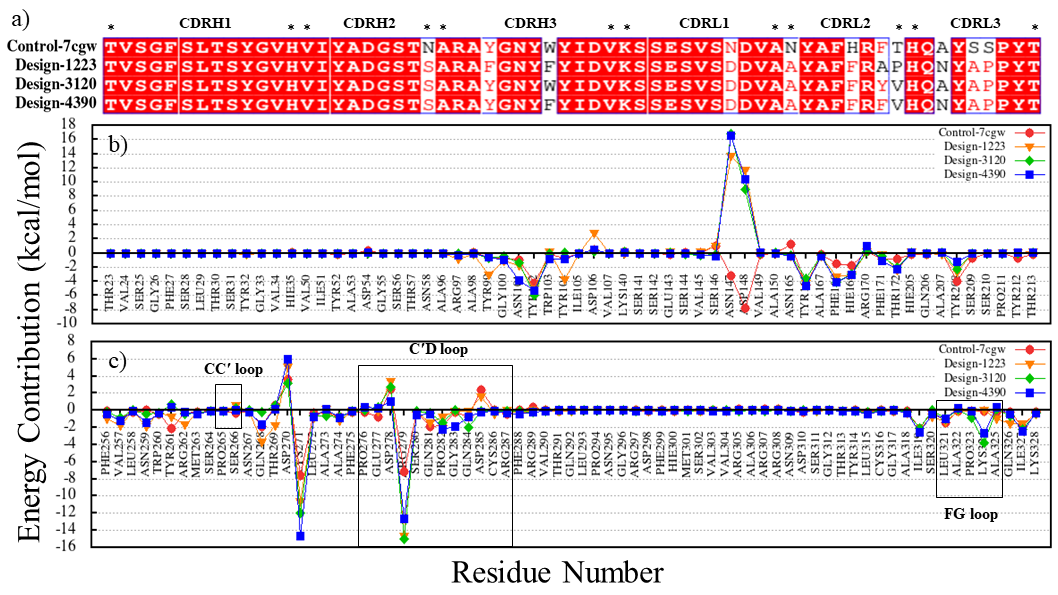


**S56 Fig. Binding free energy decomposition of the residues in the control and designed complexes.** (a) Alignment of CDR sequences in the control-7cgw (tislelizumab-PD-1) and design groups. (b) Energy contribution values of PD-1 residues from MM-PBSA. (c) Energy contribution values of CDR residues from MM-PBSA. The graphs show the binding free energy for each residue in control-7cgw (red), design-1223 (orange), design-3120 (green), and design-4390 (blue).


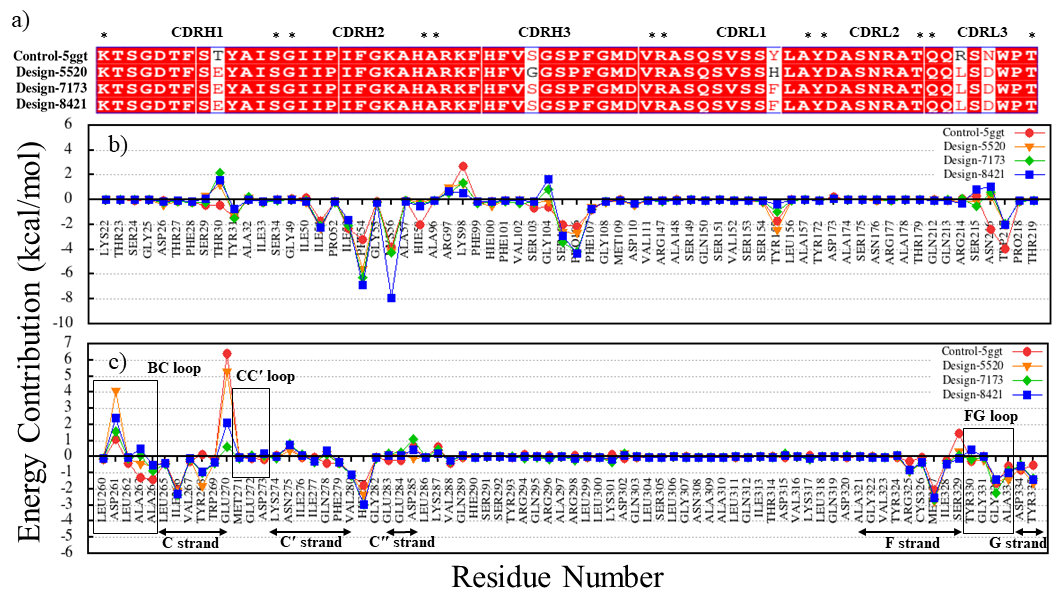


**S57 Fig. Binding free energy decomposition of the residues in the control and designed complexes.** (a) Alignment of CDR sequences in the control-5ggt (BMS-936559-PD-1) and design groups. (b) Energy contribution values of PD-L1 residues from MM-PBSA. (c) Energy contribution values of CDR residues from MM-PBSA. The graphs show the binding free energy for each residue in control-5ggt (red), design-5520 (orange), design-7173 (green), and design-8421 (blue).


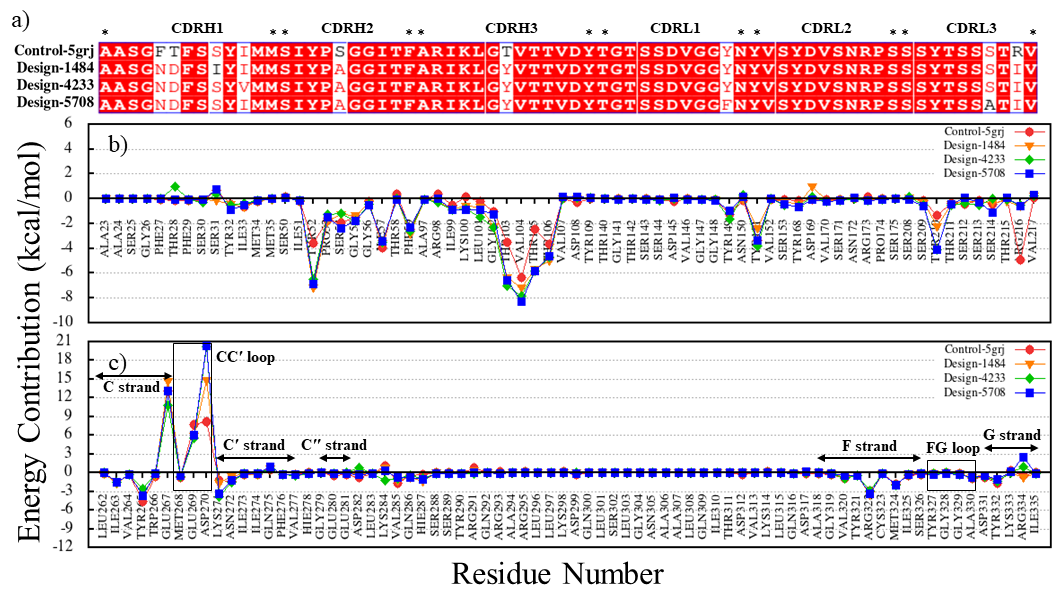


**S58 Fig. Binding free energy decomposition of the residues in the control and designed complexes.** (a) Alignment of CDR sequences in the control-5grj (avelumab-PD-L1) and design groups. (b) Energy contribution values of PD-L1 residues from MM-PBSA. (c) Energy contribution values of CDR residues from MM-PBSA. The graphs show the binding free energy for each residue in control-5grj (red), design-1484 (orange), design-4233 (green), and design-5708 (blue).


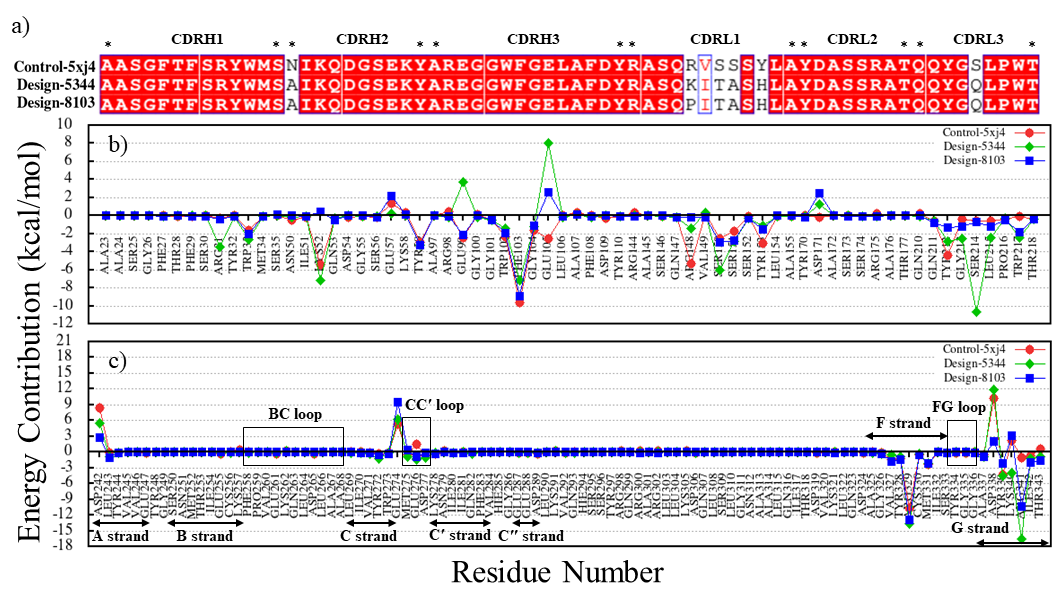


**S59 Fig. Binding free energy decomposition of the residues in the control and designed complexes.** (a) Alignment of CDR sequences in the control-5xj4 (durvalumab-PD-L1) and design groups. (b) Energy contribution values of PD-L1 residues from MM-PBSA. (c) Energy contribution values of CDR residues from MM-PBSA. The graphs show the binding free energy for each residue in control-5xj4 (red), design-5344 (green), and design-8103 (blue).


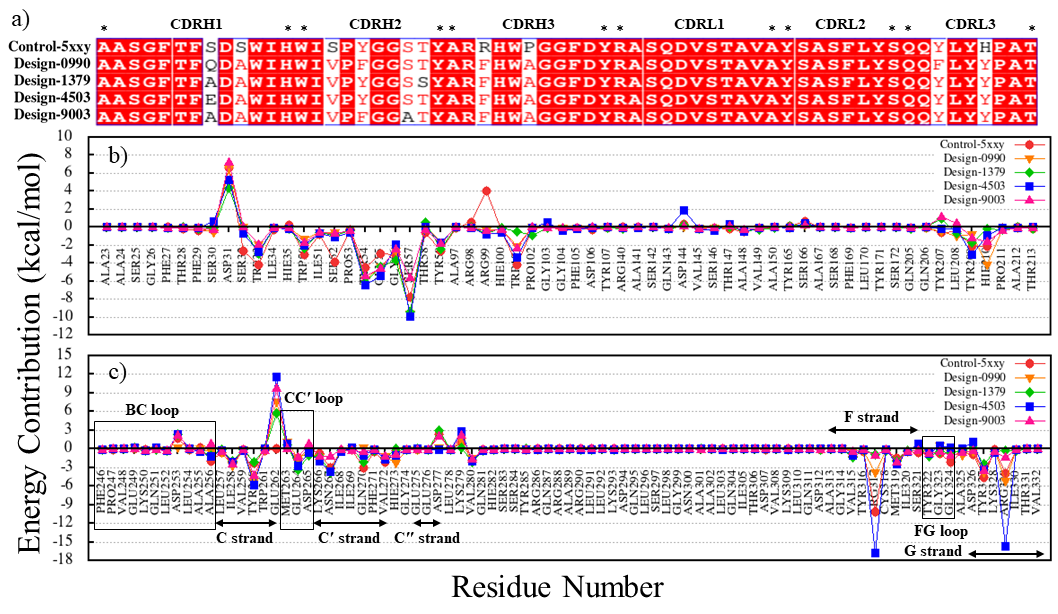


**S60 Fig. Binding free energy decomposition of the residues in the control and designed complexes.** (a) Alignment of CDR sequences in the control-5xxy (atezolizumab-PD-L1) and design groups. (b) Energy contribution values of PD-L1 residues from MM-PBSA. (c) Energy contribution values of CDR residues from MM-PBSA. The graphs show the binding free energy for each residue in control-5xxy (red), design-0990 (orange), design-1379 (green), design-4503 (blue), and design-9003 (magenta).


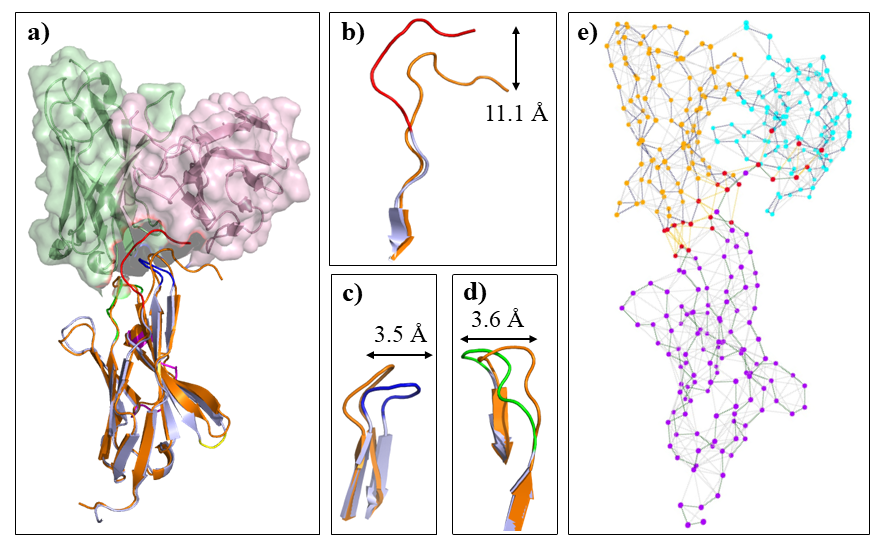


**S61 Fig. Analysis of conformational changes within PD-1 loops in complex with design-9886.** (a) Comparison of conformational changes in the PD-1 loops upon binding to design-9886 and PD-L1. The heavy chain, light chain, PD-1 in complex with design-9886, and PD-1 in complex with PD-L1 are colored pale green, light pink, light blue, and orange, respectively. The FG, BC, and N loops of PD-1 in complex with design-9886 are colored in blue, green, and red, respectively. (b) The N loop of PD-1 shifted 11.1 Å upon binding to design-9886. (c) The FG loop of PD-1 shifted 3.5 Å upon binding to design-9886. (d) The BC loop of PD-1 shifted 3.6 Å upon binding to design-9886. (e) Interaction network between PD-1 loops and the design-9886. The amino acids in the heavy chain, light chain and PD-1 are colored as cyan, gold, and violet nodes, respectively. Red nodes represent the amino acids which are participate in PD-1 interaction.


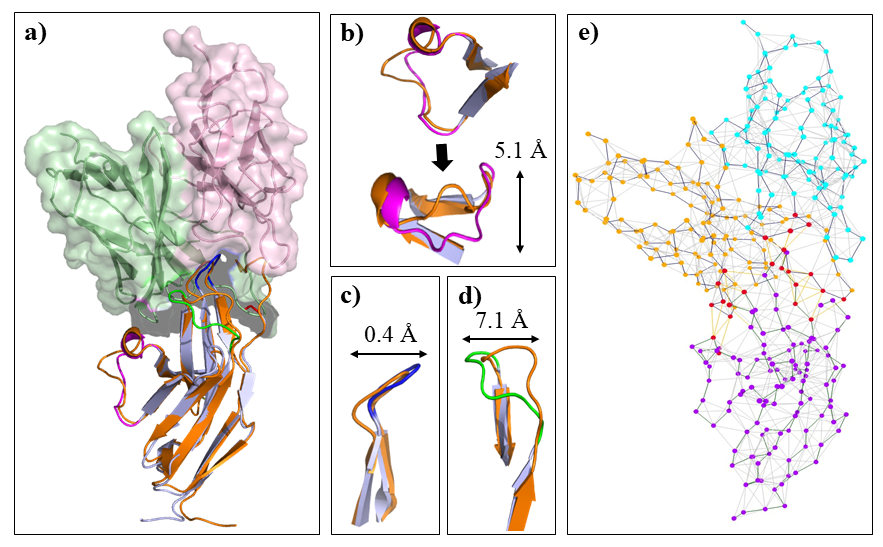


**Fig S62. Analysis of conformational changes within PD-1 loops in the design-9609 complex.** (a) Comparison of conformational changes in the PD-1 loops upon binding to design-9609 and PD-L1. The heavy chain, light chain, PD-1 in complex with design-9609, and PD-1 in complex with PD-L1 are colored pale green, light pink, light blue, and orange, respectively. The FG, BC, and N loops of PD-1 in complex with design-9609 are colored in blue, green, and red, respectively. (b) The C′D loop of PD-1 shifted 5.1 Å upon binding to design-9609. (c) The FG loop of PD-1 shifted 0.4 Å upon binding to design-9609. (d) The BC loop of PD-1 shifted 7.1 Å upon binding to design-9609. (e) Interaction network between PD-1 loops and the design-9609. The amino acids in the heavy chain, light chain and PD-1 are colored as cyan, gold, and violet nodes, respectively. Red nodes represent the amino acids which are participate in PD-1 interaction.


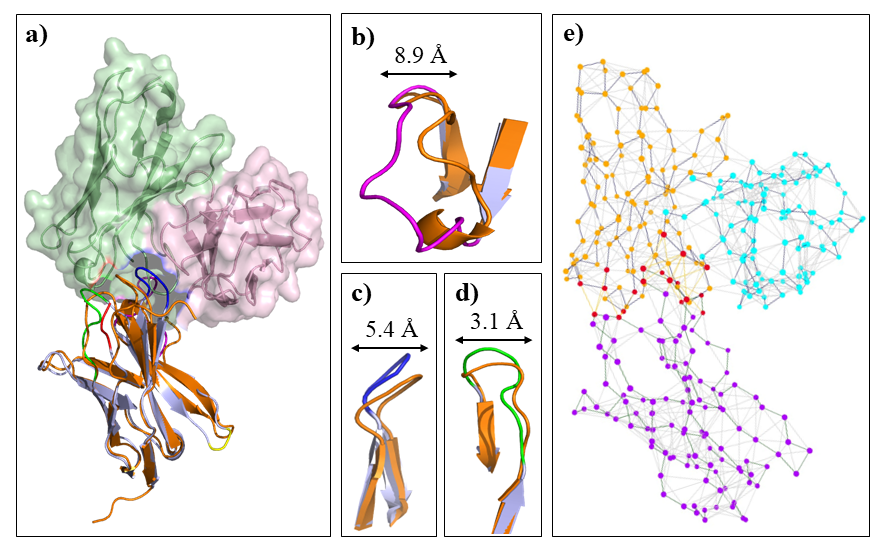


**S63 Fig. Analysis of conformational changes within PD-1 loops in complex with design-7357.** (a) Comparison of conformational changes in the PD-1 loops upon binding to design-7357 and PD-L1. The heavy chain, light chain, PD-1 in complex with design-7357, and PD-1 in complex with PD-L1 are colored pale green, light pink, light blue, and orange, respectively. The C′D, FG, and BC loops of PD-1 in complex with design-7357 are colored in magenta, blue, and green, respectively. (b) The C′D loop of PD-1 shifted 8.9 Å in the groove which was formed by the CDR loops of design-7357. (c) The FG loop of PD-1 shifted 5.4 Å upon the binding to design-7357. (d) The BC loop of PD-1 shifted 3.1 Å upon the binding to design-7357. (e) Interaction network between PD-1 loops and the design-7357. The amino acids in the heavy chain, light chain and PD-1 are colored as cyan, gold, and violet nodes, respectively. Red nodes represent the amino acids which are participate in PD-1 interaction.


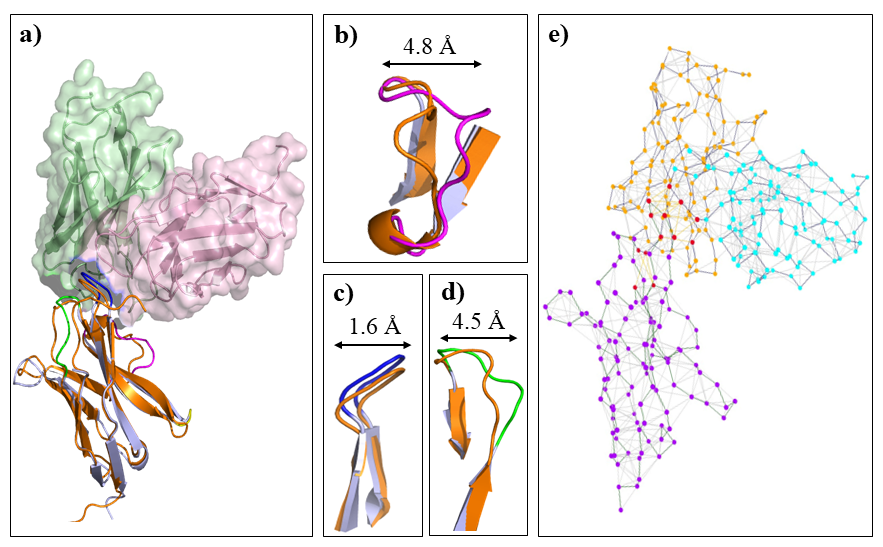


**S64 Fig.  Analysis of conformational changes within PD-1 loops in complex with design-3128.** (a) Comparison of conformational changes in the PD-1 loops upon binding to design-3128 and PD-L1. The heavy chain, light chain, PD-1 in complex with design-3128, and PD-1 in complex with PD-L1 are colored pale green, light pink, light blue, and orange, respectively. The FG, BC, and N loops of PD-1 in complex with design-3128 are colored in blue, green, and red, respectively. (b) The C′D loop of PD-1 shifted 4.8 Å upon binding to design-3128. (c) The FG loop of PD-1 shifted 1.6 Å upon binding to design-3128. (d) The BC loop of PD-1 shifted 4.5 Å upon binding to design-3128. (e) Interaction network between PD-1 loops and the design-3128. The amino acids in the heavy chain, light chain and PD-1 are colored as cyan, gold, and violet nodes, respectively. Red nodes represent the amino acids which are participate in PD-1 interaction.


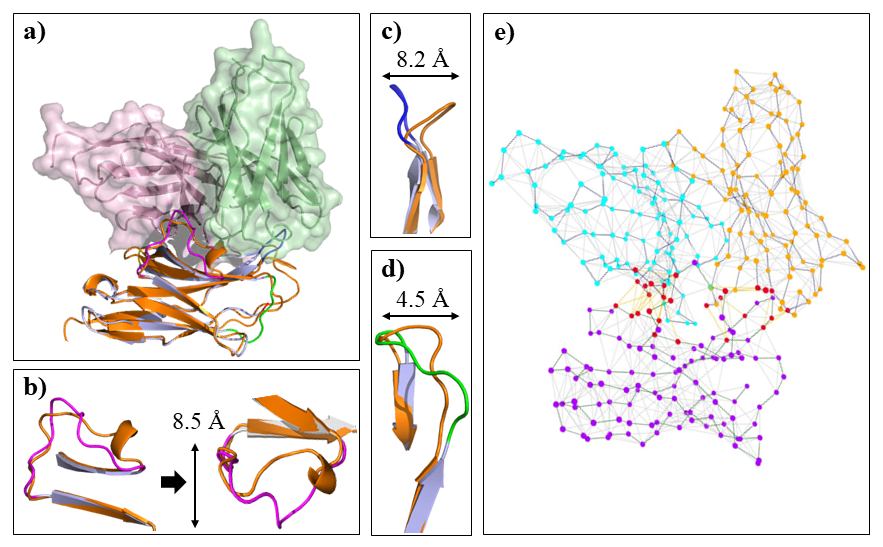


**S65 Fig.  Analysis of conformational changes within PD-1 loops in complex with design-8773**. (a) Comparison of conformational changes in the PD-1 loops upon binding to design-8773 and PD-L1. The heavy chain, light chain, PD-1 in complex with design-8773, and PD-1 in complex with PD-L1 are colored pale green, light pink, light blue, and orange, respectively. The C′D, FG, and BC loops of PD-1 in complex with design-8773 are colored in magenta, blue, and green, respectively. (b) The C′D loop of PD-1 shifted 8.5 Å in the groove which was formed by the CDR loops of design-8773. (c) The FG loop of PD-1 shifted 8.2 Å upon binding to design-8773. (d) The BC loop of PD-1 shifted 4.5 Å upon binding to design-8773. (e) Interaction network between PD-1 loops and the design-8773. The amino acids in the heavy chain, light chain and PD-1 are colored as cyan, gold, and violet nodes, respectively. Red nodes represent the amino acids which are participate in PD-1 interaction.


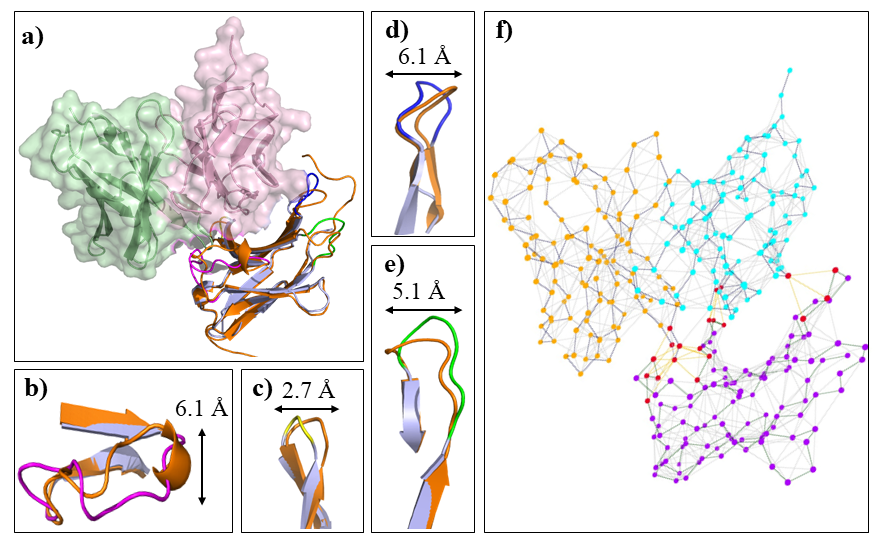


**S66 Fig.  Analysis of conformational changes within PD-1 loops in complex with design-1223.** (a) Comparison of conformational changes in the PD-1 loops upon binding to design-1223 and PD-L1. The heavy chain, light chain, PD-1 in complex with design-1223, and PD-1 in complex with PD-L1 are colored pale green, light pink, light blue, and orange, respectively. The FG, BC, and N loops of PD-1 in complex with design-1223 are colored in blue, green, and red, respectively. (b) The C′D loop of PD-1 shifted 6.1 Å upon binding to design-1223. (c) The CC′ loop of PD-1 shifted 2.7 Å upon binding to design-1223. (d) The FG loop of PD-1 shifted 6.1 Å upon binding to design-1223. (e) The BC loop of PD-1 shifted 5.1 Å upon the binding to design-1223. (f) Interaction network between PD-1 loops and the design-1223. The amino acids in the heavy chain, light chain and PD-1 are colored as cyan, gold, and violet nodes, respectively. Red nodes represent the amino acids which are participate in PD-1 interaction.


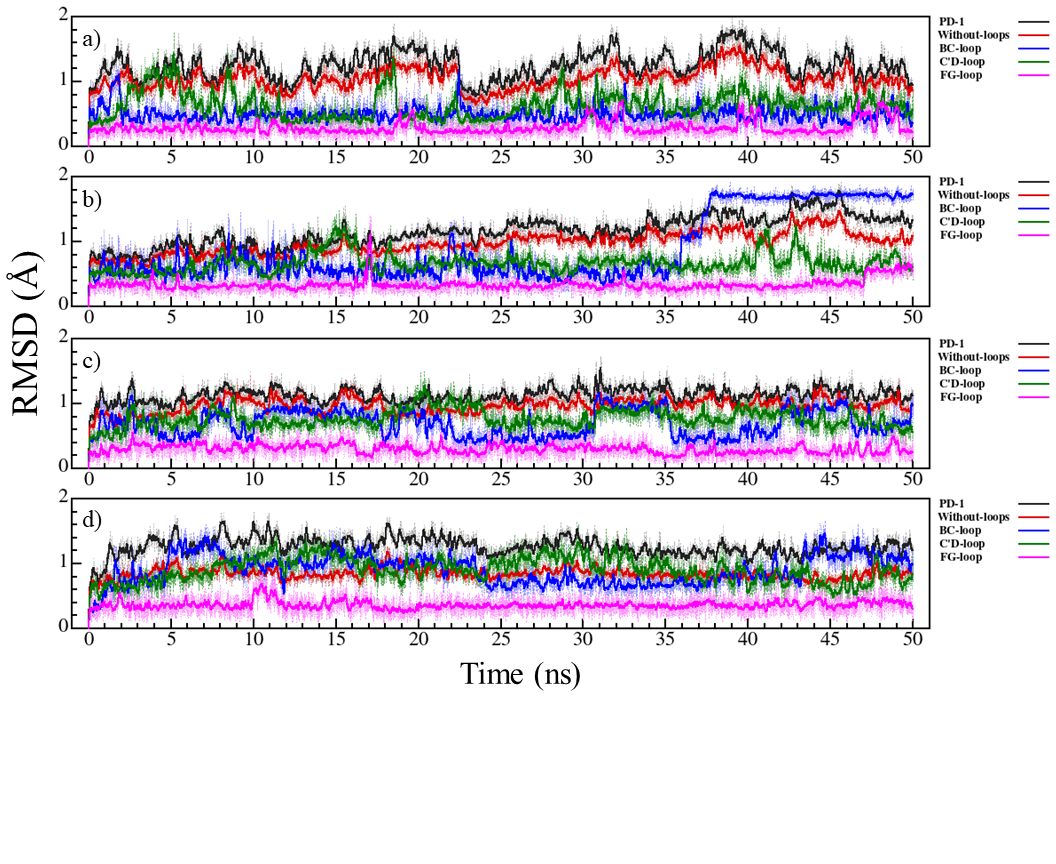


**S67 Fig. RMSD graphs for PD-1 with and without loops during 50 ns of MD simulation.** PD-1 (black), PD-1 without BC, C′D and FG loops (red), BC loop (blue), C′D loop (green), and FG loop (magenta). (a) control-5ggs (pembrolizumab-PD-1), (b) design-1022, (c) design-3753, (d) design-6013.


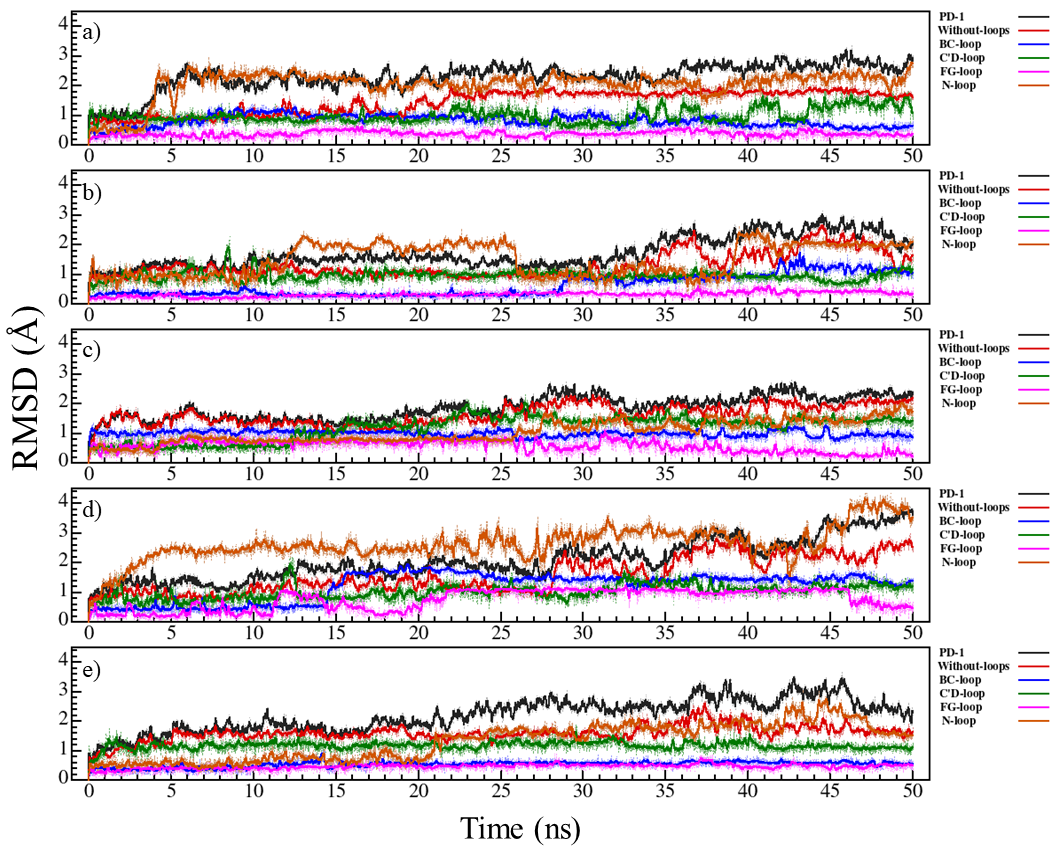


**S68 Fig. RMSD graphs for PD-1 with and without loops during 50 ns of MD simulation.** PD-1 (black), PD-1 without BC, C′D and FG loops (red), BC loop (blue), C′D loop (green), FG loop (magenta), and N loop (orange). (a) control-5wt9 (nivolumab-PD-1). (b) design-1799. (c) design-3713. (d) design-9835. (e) design-9886.


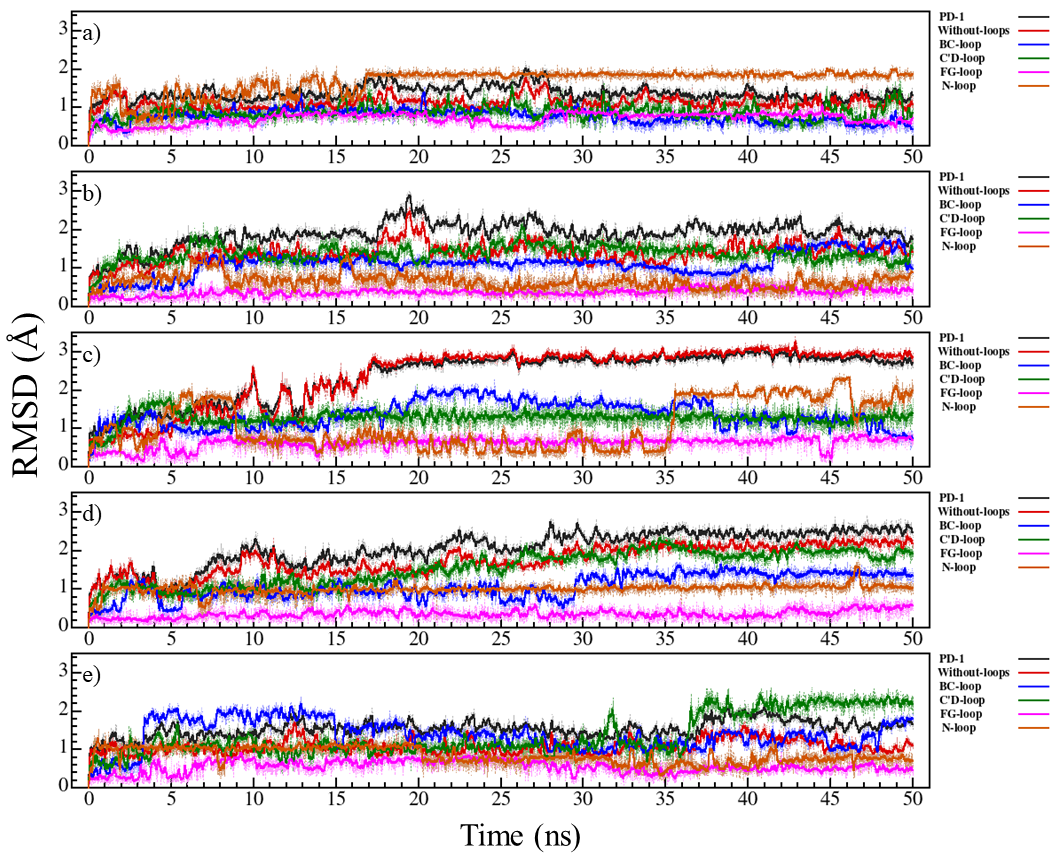


**S69 Fig. RMSD graphs for PD-1 with and without loops during 50 ns of MD simulation.** PD-1 (black), PD-1 without BC, C′D and FG loops (red), BC loop (blue), C′D loop (green), FG loop (magenta), and N loop (orange). (a) control-6jbt (toripalimab-PD-1). (b) design-3941. (c) design-5880. (d) design-8052. (e) design-9609. (f) design-9937.


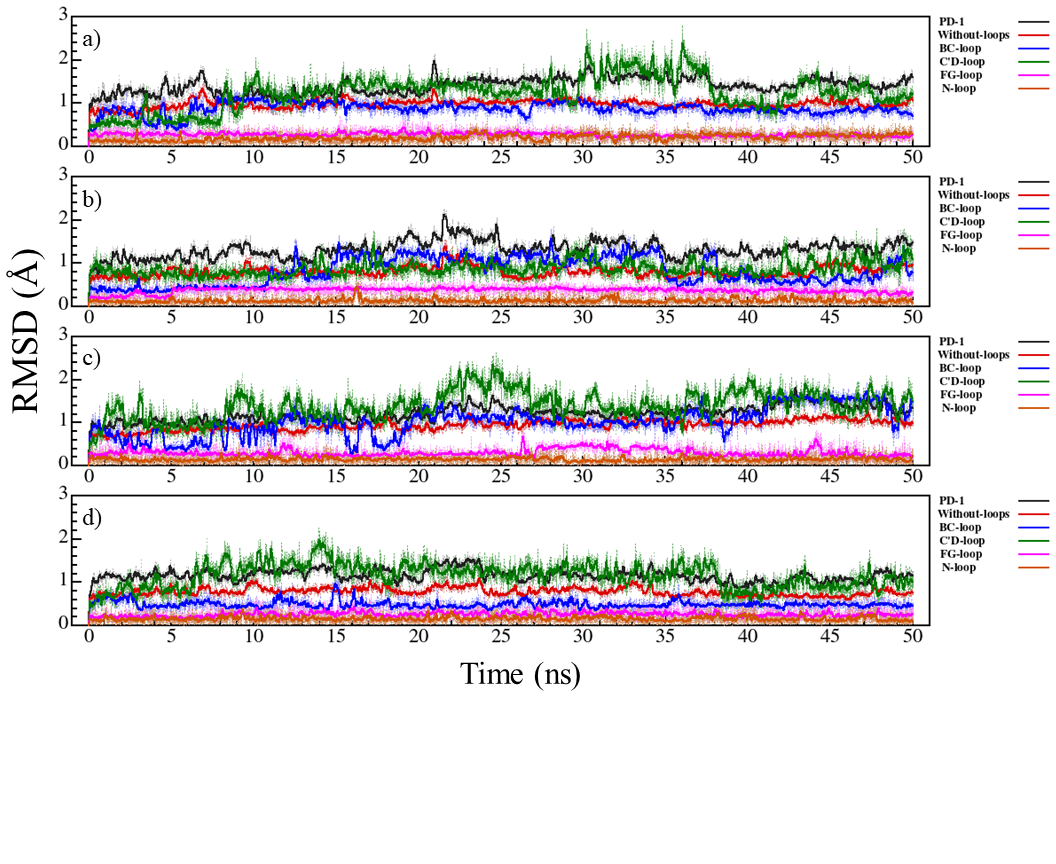


**S70 Fig. RMSD graphs for PD-1 with and without loops during 50 ns of MD simulation.** PD-1 (black), PD-1 without BC, C′D and FG loops (red), BC loop (blue), C′D loop (green), FG loop (magenta), and N loop (orange). (a) control-6jjp (MW11-h317-PD-1). (b) design-0118. (c) design-2207. (d) design-7357.


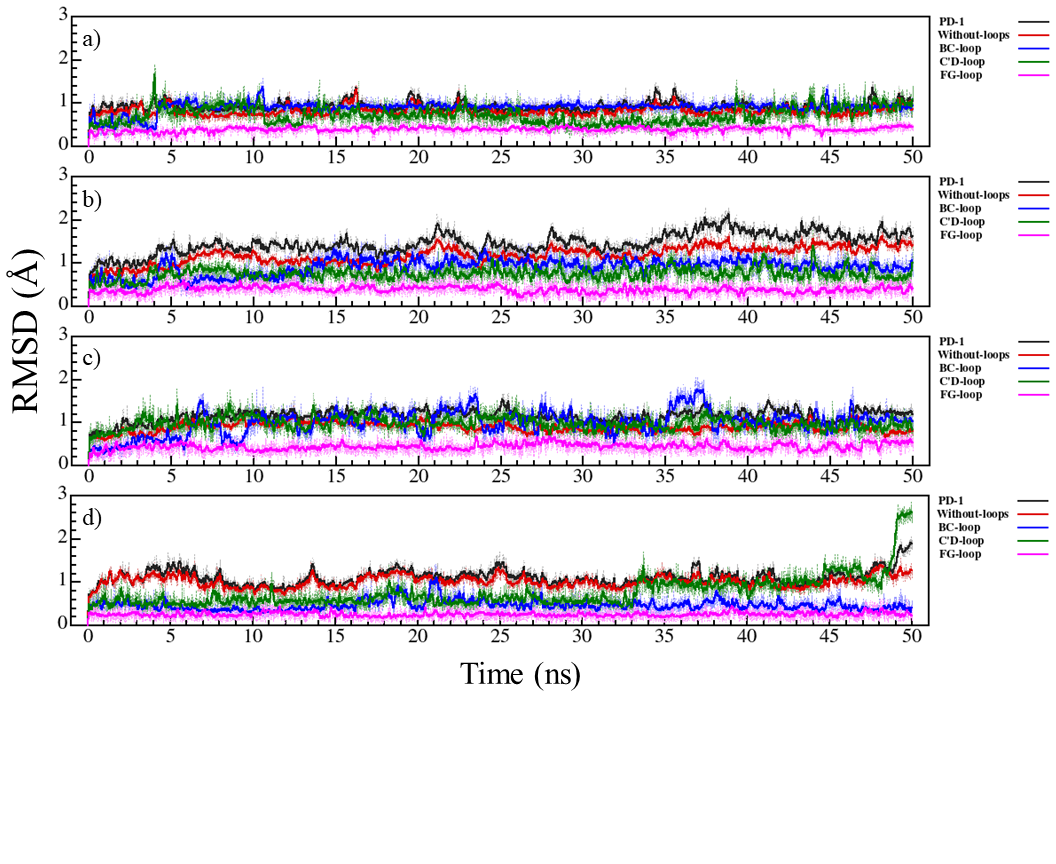


**S71 Fig. S69 Fig. RMSD graphs for PD-1 with and without loops during 50 ns of MD simulation.** PD-1 (black), PD-1 without BC, C′D and FG loops (red), BC loop (blue), C′D loop (green), and FG loop (magenta). (a) control-6k0y (mAb059c-PD-1). (b) design-1483. (c) design-3128. (d) design-4855.


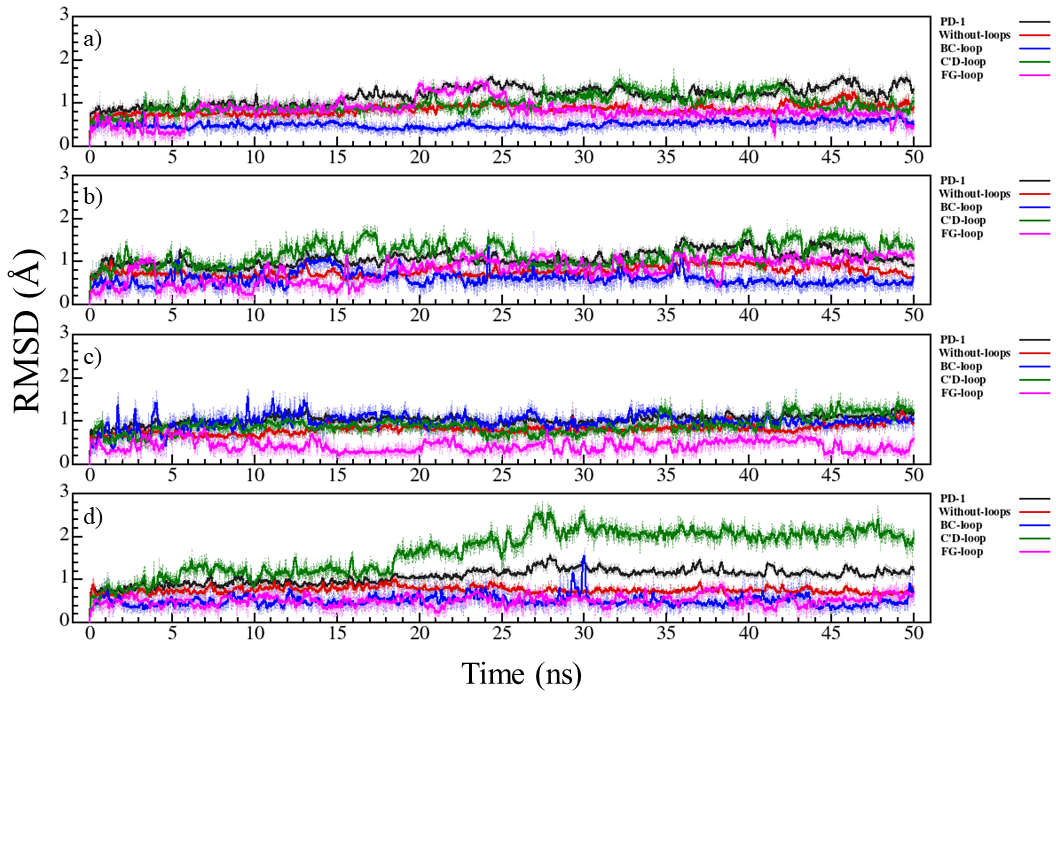


**S72 Fig. RMSD graphs for PD-1 with and without loops during 50 ns of MD simulation.** PD-1 (black), PD-1 without BC, C′D and FG loops (red), BC loop (blue), C′D loop (green), and FG loop (magenta). (a) control-6xkr (sasanlimab-PD-1). (b) design-0669. (c) design-0719. (d) design-8773.


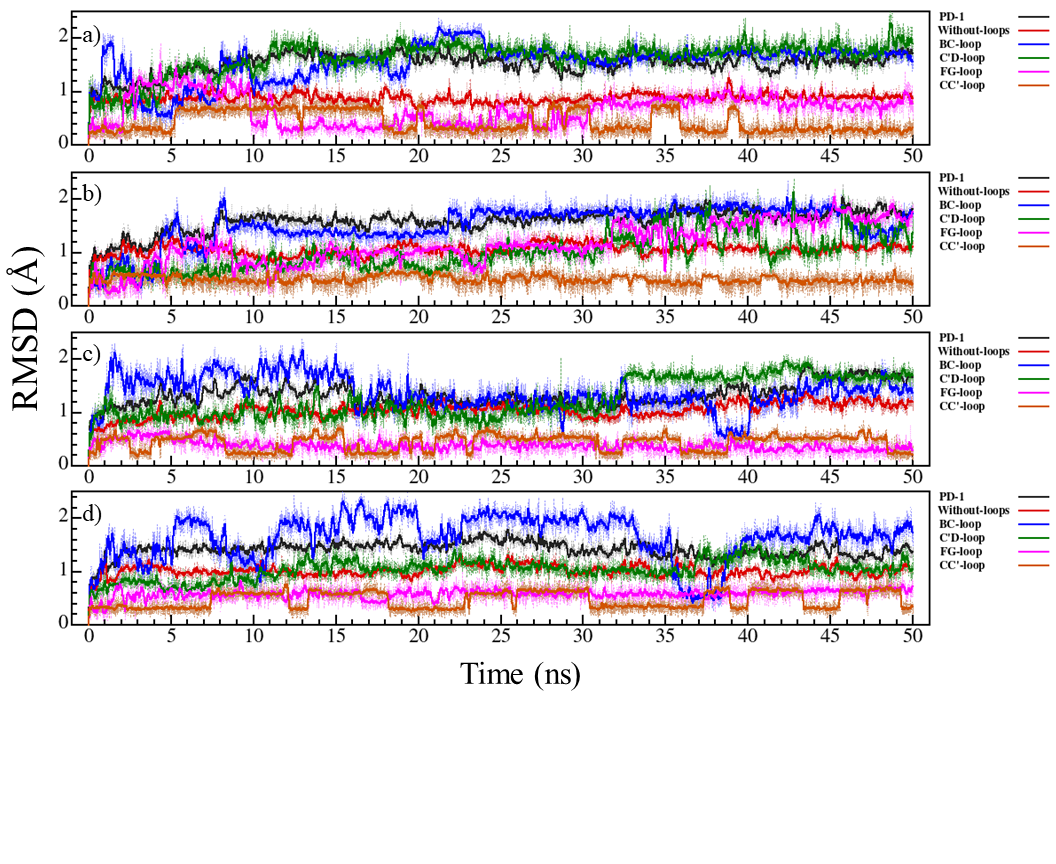


**S73 Fig. RMSD graphs for PD-1 with and without loops during 50 ns of MD simulation.** PD-1 (black), PD-1 without BC, C′D and FG loops (red), BC loop (blue), C′D loop (green), FG loop (magenta), and N loop (orange). (a) control-7cgw (tislelizumab-PD-1). (b) design-1223. (c) design-3120. (d) design-4390.

**S1 Table. The interface characteristics of the antibody-PD-1 and antibody-PD-L1 complexes were calculated by RosettaInterfaceAnalyzer.**

|  | *total-score^a^* | *dG^b^* | *dG/dSASA^c^* | *hbond-int^d^* | *delta_unsathbonds^e^* | *dSASA-int^f^* | *dSASA-hphobic^g^* | *dSASA-polar^h^* |
| --- | --- | --- | --- | --- | --- | --- | --- | --- |
| *Control-5ggs* | -1040.69 | -58.1373 | -2.64502 | 15 | 9 | 2198 | 1135.5 | 1062.49 |
| *Design-1022* | -1043.942 | -59.0409 | -2.71164 | 13 | 9 | 2177.32 | 1170.32 | 1007 |
| *Design-3753* | -1043.040 | -58.1324 | -2.66747 | 13 | 8 | 2179.31 | 1180.94 | 998.373 |
| *Design-6013* | -1041.094 | -58.2503 | -2.64393 | 13 | 10 | 2203.17 | 1164.21 | 1038.96 |
| *Control-5wt9* | -1119.788 | -39.0138 | -2.66536 | 9 | 8 | 1463.73 | 675.79 | 787.942 |
| *Design-1799* | -1133.746 | -45.1498 | -3.16364 | 11 | 5 | 1427.15 | 732.8 | 694.347 |
| *Design-3713* | -1121.099 | -44.8084 | -3.16877 | 10 | 3 | 1414.06 | 735.352 | 678.71 |
| *Design-9835* | -1134.952 | -46.0289 | -3.11045 | 10 | 3 | 1479.82 | 797.372 | 682.445 |
| *Design-9886* | -1130.743 | -44.466 | -3.11819 | 10 | 3 | 1426.02 | 787.373 | 638.65 |
| *Control-6jbt* | -1104.923 | -32.4556 | -1.8182 | 7 | 9 | 1785.03 | 936.909 | 848.125 |
| *Design-3941* | -1108.093 | -38.9062 | -2.2862 | 7 | 12 | 1701.79 | 983.39 | 718.395 |
| *Design-5880* | -1108.444 | -40.1905 | -2.38676 | 7 | 10 | 1683.89 | 1022.22 | 661.667 |
| *Design-8052* | -1110.318 | -39.1618 | -2.31964 | 6 | 8 | 1688.27 | 1042.02 | 646.256 |
| *Design-9609* | -1112.326 | -39.0501 | -2.29341 | 6 | 10 | 1702.71 | 1008.42 | 694.291 |
| *Design-9937* | -1109.745 | -39.5147 | -2.31911 | 6 | 8 | 1703.87 | 986.161 | 717.712 |
| *Control-6jjp* | -1116.095 | -47.8669 | -2.79844 | 10 | 11 | 1710.48 | 947.926 | 762.556 |
| *Design-118* | -1121.091 | -50.4086 | -2.84142 | 11 | 8 | 1774.06 | 996.561 | 777.503 |
| *Design-2207* | -1123.070 | -51.0697 | -2.89695 | 11 | 9 | 1762.88 | 993.332 | 769.548 |
| *Design-7357* | -1121.386 | -52.1637 | -2.85052 | 11 | 9 | 1829.97 | 1070.67 | 759.305 |
| *Control-6k0y* | -1252.773 | -54.92 | -3.9211 | 11 | 5 | 1400.63 | 706.092 | 694.534 |
| *Design-1483* | -1251.103 | -54.4094 | -4.13902 | 11 | 5 | 1314.55 | 687.242 | 627.308 |
| *Design-3128* | -1253.999 | -54.8652 | -4.06571 | 11 | 5 | 1349.46 | 684.556 | 664.905 |
| *Design-4855* | -1254.022 | -55.2639 | -4.09788 | 11 | 5 | 1348.6 | 686.414 | 662.182 |
| *Control-6xkr* | -1077.740 | -67.5875 | -3.24925 | 16 | 7 | 2080.1 | 1052.78 | 1027.31 |
| *Design-669* | -1081.302 | -69.0659 | -3.28492 | 16 | 7 | 2102.52 | 1085.12 | 1017.4 |
| *Design-719* | -1078.978 | -69.296 | -3.32382 | 16 | 7 | 2084.83 | 1065.65 | 1019.18 |
| *Design-8773* | -1079.368 | -69.9914 | -3.35814 | 16 | 7 | 2084.23 | 1080.86 | 1003.38 |
| *Control-7cgw* | -1033.152 | -54.758 | -2.77503 | 12 | 11 | 1973.24 | 947.405 | 1025.83 |
| *Design-1223* | -1035.597 | -59.3066 | -3.00043 | 12 | 7 | 1976.6 | 1100.43 | 876.175 |
| *Design-3120* | -1042.956 | -59.8174 | -2.98492 | 12 | 6 | 2003.99 | 1041.48 | 962.507 |
| *Design-4390* | -1039.282 | -60.2057 | -3.01044 | 12 | 6 | 1999.9 | 1054.51 | 945.389 |
| *Control-5ggt* | -1086.287 | -42.1543 | -2.98478 | 5 | 7 | 1412.31 | 867.236 | 545.07 |
| *Design-5520* | -1087.590 | -42.2487 | -3.0444 | 6 | 7 | 1387.75 | 868.125 | 519.625 |
| *Design-7173* | -1087.662 | -43.5579 | -3.05941 | 5 | 7 | 1423.74 | 899.251 | 524.486 |
| *Design-8421* | -1088.247 | -43.1905 | -3.0554 | 5 | 7 | 1413.58 | 888.097 | 525.482 |
| *Control-5grj* | -1063.568 | -63.3364 | -3.57195 | 16 | 8 | 1773.16 | 888.481 | 884.68 |
| *Design-1484* | -1073.040 | -65.1081 | -3.63706 | 14 | 7 | 1790.13 | 932.251 | 857.878 |
| *Design-4233* | -1070.431 | -64.7461 | -3.63957 | 15 | 7 | 1778.95 | 902.368 | 876.583 |
| *Design-5708* | -1072.605 | -66.1955 | -3.80789 | 15 | 7 | 1738.38 | 920.325 | 818.053 |
| *Control-5xj4* | -1139.166 | -39.6586 | -2.53517 | 11 | 6 | 1564.34 | 800.353 | 763.983 |
| *Design-5344* | -1139.001 | -40.3788 | -2.51883 | 11 | 6 | 1603.08 | 870.151 | 732.928 |
| *Design-8103* | -1140.133 | -40.2086 | -2.631 | 11 | 6 | 1528.26 | 842.694 | 685.571 |
| *Control-5xxy* | -1005.265 | -64.6807 | -3.35811 | 12 | 22 | 1926.11 | 1116.05 | 810.061 |
| *Design-990* | -1009.838 | -66.1703 | -3.50152 | 11 | 17 | 1889.76 | 1197.65 | 692.108 |
| *Design-1379* | -1009.386 | -67.3249 | -3.46849 | 12 | 15 | 1941.04 | 1166.88 | 774.161 |
| *Design-4503* | -1009.991 | -69.1986 | -3.42916 | 13 | 17 | 2017.95 | 1167.6 | 850.352 |
| *Design-9003* | -1013.426 | -68.4729 | -3.61223 | 11 | 17 | 1895.59 | 1232.51 | 663.077 |

**^a^total-score:** The score of the entire complex

**^b^dG:** The binding energy

**^c^dG/dSASA:** The binding density

**^d^hbonds_int:** Total cross-interface hydrogen bonds

**^e^delta_unsathbonds:** The number of buried unsatisfied hydrogen bonds at the interface

**^f^dSASA_int:** The solvent accessible area buried at the interface

**^g^dSASA-hydrophobic:** The amount of change in the hydrophobic part of dSASA

**^h^dSASA-polar:** The change in the polar part of dSASA

**S2 Table. MMPBSA binding free energies (kcal·mol ^-1^) for control and design complexes.**

|  | *∆G_binding^a^* | *EN_polar* | *EPB^b^* | *EEL^c^* | *VDWAALS^d^* |
| --- | --- | --- | --- | --- | --- |
| *PD-1-PD-L1* | -36.3006 ± 0.6670 | -12.2053 | 368.5067 | -294.6720 | -97.9300 |
| *Control-5ggs* | -63.5046 ± 1.0689 | -13.1622 | 211.3707 | -141.6487 | -120.0644 |
| *Design-1022* | -71.8580 ± 0.8023 | -11.9187 | 210.7278 | -158.6573 | -112.0099 |
| *Design-3753* | -84.5037 ± 0.9835 | -12.5994 | 228.9138 | -178.7286 | -122.0894 |
| *Design-6013* | -76.0652 ± 1.0441 | -13.2310 | 275.1449 | -217.5797 | -120.3994 |
| *Control-5wt9* | -45.9166 ± 0.6162 | -8.3022 | 220.2949 | -188.1658 | -69.7435 |
| *Design-1799* | -28.9920 ± 0.6232 | -7.3594 | 185.1880 | -156.0977 | -50.7228 |
| *Design-3713* | -25.2546 ± 0.7286 | -6.5267 | 164.5513 | -130.0838 | -53.1953 |
| *Design-9835* | -23.4566 ± 0.7279 | -7.0713 | 180.8681 | -142.7150 | -54.5383 |
| *Design-9886* | -53.3361 ± 0.7075 | -8.1761 | 229.8903 | -203.2274 | -71.8229 |
| *Control-6jbt* | -57.2988 ± 1.2210 | -9.6246 | 377.5447 | -347.7800 | -77.4389 |
| *Design-3941* | -57.4944 ± 0.9084 | -9.9253 | 300.8474 | -260.9954 | -87.4211 |
| *Design-5880* | -59.9833 ± 0.7305 | -8.8057 | 196.3309 | -170.5891 | -76.9193 |
| *Design-8052* | -38.2312 ± 0.7049 | -8.9574 | 274.9222 | -203.0716 | -74.1244 |
| *Design-9609* | -66.2888 ± 0.6987 | -9.2896 | 317.9428 | -290.8447 | -84.0974 |
| *Design-9937* | -56.8644 ± 1.1921 | -9.9594 | 281.1409 | -241.9234 | -86.1225 |
| *Control-6jjp* | -78.3314 ± 0.6077 | -10.3637 | 275.9774 | -266.9189 | -77.0262 |
| *Design-118* | -67.6653 ± 0.6881 | -10.4243 | 304.8807 | -290.7395 | -71.3823 |
| *Design-2207* | -76.4657 ± 0.8719 | -10.4272 | 304.6621 | -291.6020 | -79.0986 |
| *Design-7357* | -80.0839 ± 0.9019 | -11.1178 | 256.1507 | -243.8937 | -81.2231 |
| *Control-6k0y* | -62.1815 ± 0.6120 | -7.5072 | 210.2384 | -204.7890 | -60.1236 |
| *Design-1483* | -65.2533 ± 0.4896 | -6.9540 | 61.6082 | -60.0528 | -59.8574 |
| *Design-3128* | -67.7862 ± 0.5036 | -7.5754 | 59.2986 | -61.0846 | -58.4248 |
| *Design-4855* | -59.3813 ± 0.4697 | -7.5641 | 85.7730 | -86.7508 | -50.8394 |
| *Control-6xkr* | -67.5520± 0.6288 | -12.7660 | 244.3635 | -192.8176 | -106.3320 |
| *Design-669* | -86.5503 ± 0.7275 | -13.2291 | 302.7844 | -263.3775 | -112.7281 |
| *Design-719* | -74.4536 ± 0.6898 | -12.9840 | 313.8567 | -266.3762 | -108.9501 |
| *Design-8773* | -87.8527 ± 0.7346 | -12.8635 | 248.5621 | -208.0094 | -115.5420 |
| *Control-7cgw* | -69.1484 ± 0.9933 | -10.6475 | 198.8348 | -169.6457 | -87.6900 |
| *Design-1223* | -66.3825 ± 0.8905 | -12.0646 | 348.8838 | -296.5649 | -106.6369 |
| *Design-3120* | -50.7525 ± 0.7756 | -10.4295 | 301.9074 | -253.4811 | -88.7493 |
| *Design-4390* | -59.0010 ± 0.9997 | -11.5392 | 334.6023 | -277.5610 | -104.5030 |
| *Control-5ggt* | -43.6594 ± 0.6323 | -8.7001 | 179.5114 | -135.5261 | -78.9446 |
| *Design-5520* | -46.0070 ± 0.8771 | -8.2333 | 145.7664 | -102.8552 | -80.6350 |
| *Design-7173* | -53.5563 ± 0.6880 | -9.6456 | 169.3057 | -120.2914 | -92.9250 |
| *Design-8421* | -48.6919 ± 0.8411 | -8.4332 | 162.0082 | -125.4270 | -76.8399 |
| *Control-5grj* | -52.9111 ± 0.9894 | -10.3188 | 291.5815 | -239.0225 | -95.1512 |
| *Design-1484* | -57.0060 ± 0.6449 | -10.4273 | 253.1840 | -206.5268 | -93.2359 |
| *Design-4233* | -60.6421 ± 0.5616 | -9.6380 | 259.7454 | -218.7672 | -91.9822 |
| *Design-5708* | -55.4120 ± 0.5494 | -9.7065 | 238.7751 | -196.6032 | -87.8774 |
| *Control-5xj4* | -53.3737 ± 0.6736 | -8.9439 | 281.6083 | -250.1673 | -75.8707 |
| *Design-5344* | -91.9746 ± 0.7648 | -9.8335 | 389.1429 | -380.0630 | -91.2210 |
| *Design-8103* | -62.9899 ± 1.1393 | -8.8248 | 268.4529 | -233.3424 | -89.2759 |
| *Control-5xxy* | -92.8574 ± 1.2213 | -11.3072 | 225.6802 | -194.1754 | -113.0550 |
| *Design-990* | -75.8208 ± 1.0071 | -9.5161 | 185.0168 | -156.5105 | -94.8111 |
| *Design-1379* | -68.1533 ± 0.7771 | -10.9205 | 221.7871 | -173.7705 | -105.2493 |
| *Design-4503* | -97.6586 ± 0.9169 | -11.7572 | 288.2266 | -257.4467 | -116.6812 |
| *Design-9003* | -51.6839 ± 0.8393 | -10.5893 | 251.9205 | -191.5943 | -101.4202 |

**^a^DELTA G binding:** final estimated binding free energy (kcal/mol)

**^b^EPB:** The electrostatic contribution to the solvation free energy calculated by PB

**^c^EEL:** electrostatic energy as calculated by the MM force field.

**^d^VDWAALS:** van der Waals contribution from MM.

**S3 Table. The types of interactions between the CDRs of the designs and binding sites 1-11 during simulations.**

| **Type of interactions** | **Binding sites** | **Designs** |
| --- | --- | --- |
| Hydrogen bond | 5, 6, 7, 9, 10, and 11 | design-3128 (S44c Fig), design-4855 (S44d Fig), design-0669 (S45b Fig), design-0719 (S45c Fig), design-4390 (S46d Fig), design-4233 (S48c Fig), design-5344 (S49b Fig), and design-4503 (S50d Fig). |
| Hydrophobic interaction | 1, 3, 4, 5, 6, 8, 9, and 10 | design-1022 (Fig 8b), design-3753 (Fig 8c), design-6013 (Fig 8d), design-3941 (S42b Fig), design-5880 (S42c Fig), design-9609 (S42e Fig), design-9937 (S42f Fig), design-7357 (S43d Fig), design-1483 (S44b Fig), design-0669 (S45b Fig), design-0719 (S45c Fig), design-8773 (S45d Fig), design-5520 (S47b Fig), design-7173 (S47c Fig), design-8421 (S47d Fig), design-1484 (S48b Fig), design-4233 (S48c Fig), design-5708 (S48d Fig), and design-8103 (S49c Fig). |
| Salt bridge | 2, 3, 5, and 10 | design-9886 (S41e Fig), design-9937 (S42f Fig), design-1483 (S44b Fig), design-5344 (S49b Fig), and design-8103 (S49c Fig). |

**S4 Table. List of key residues of PD-1 and PD-L1 receptors at binding sites 1-11.**

|  | Residues  MMPBSA (< -2 kcal.mol^-1^) Rosetta (< -0.5 REU) Common residues | | |
| --- | --- | --- | --- |
| Binding sites 1 | \| V266 \| T278 \| Y280 \| P285 \| \| --- \| --- \| --- \| --- \| \| R288 \| S289 \| Q290 \| P291 \| \| I328 \| L330 \| K333 \| I336 \| | \| F265 \| V266 \| N268 \| Y270 \| \| --- \| --- \| --- \| --- \| \| Q277 \| K280 \| P285 \| D287 \| \| R288 \| S289 \| Q290 \| P291 \| \| I328 \| L330 \| K333 \|  \| | \| V266 \| Y280 \| P285 \| R288 \| \| --- \| --- \| --- \| --- \| \| S289 \| Q290 \| P291 \| I328 \| \| L330 \| K333 \|  \|  \| |
| Binding sites 2 | \| L225 \| D226 \| R230 \| P231 \| \| --- \| --- \| --- \| --- \| \| T259 \| S260 \| P330 \| K331 \| \| A332 \|  \|  \|  \| | \| I225 \| D226 \| D229 \| T259 \| \| --- \| --- \| --- \| --- \| \| L328 \| A329 \| 9330 \| K331 \| \| A332 \|  \|  \|  \| | \| I225 \| D226 \| T259 \| P330 \| \| --- \| --- \| --- \| --- \| \| K331 \| A332 \|  \|  \| |
| Binding sites 3 | \| P239 \| T240 \| T267 \| R294 \| \| --- \| --- \| --- \| --- \| \| I334 \| P338 \| K339 \| E341 \| \| I342 \| K434 \|  \|  \| | \| P239 \| T267 \| S270 \| N274 \| \| --- \| --- \| --- \| --- \| \| R294 \| I334 \| L336 \| A337 \| \| P338 \| K339 \| A340 \| E341 \| \| I342 \| K343 \| E344 \|  \| | \| P239 \| T267 \| R294 \| I334 \| \| --- \| --- \| --- \| --- \| \| P338 \| K339 \| E341 \| I342 \| \| K343 \|  \|  \|  \| |
| Binding sites 4 | \| S255 \| K271 \| R279 \| S280 \| \| --- \| --- \| --- \| --- \| \| I319 \| L321 \| A322 \| P323 \| \| K324 \|  \|  \|  \| | \| E254 \| S255 \| K271 \| P276 \| \| --- \| --- \| --- \| --- \| \| E277 \| D278 \| R279 \| S280 \| \| L321 \| P323 \| K324 \| A325 \| | \| S255 \| K271 \| R279 \| S280 \| \| --- \| --- \| --- \| --- \| \| L321 \| P323 \| K324 \|  \| |
| Binding sites 5 | \| R284 \| L326 \| A327 \| P328 \| \| --- \| --- \| --- \| --- \| \| K329 \| A330 \|  \|  \| | \| E309 \| S310 \| V312 \| P331 \| \| --- \| --- \| --- \| --- \| \| D333 \| R334 \| I374 \| L376 \| \| A377 \| P378 \| K379 \| A380 \| \| I382 \|  \|  \|  \| |  |
| Binding sites 6 | \| Y270 \| Q277 \| T278 \| K280 \| \| --- \| --- \| --- \| --- \| \| S289 \| Q290 \| P291 \| I328 \| \| L330 \| P332 \| I336 \|  \| | \| N268 \| Y270 \| Q277 \| T278 \| \| --- \| --- \| --- \| --- \| \| D279 \| K280 \| D287 \| S289 \| \| P291 \| G292 \| I328 \| L330 \| \| P332 \| A334 \| I336 \| Q335 \| \| E338 \|  \|  \|  \| | \| Y270 \| Q277 \| T278 \| K280 \| \| --- \| --- \| --- \| --- \| \| A289 \| P291 \| I328 \| L330 \| \| P332 \| I336 \|  \|  \| |
| Binding sites 7 | \| N259 \| Q268 \| K271 \| R279 \| \| --- \| --- \| --- \| --- \| \| Q281 \| R282 \| G238 \| Q284 \| \| I319 \| L321 \| K324 \| I327 \| | \| V257 \| Y261 \| N267 \| Q268 \| \| --- \| --- \| --- \| --- \| \| T269 \| D270 \| K271 \| L272 \| \| D278 \| R279 \| I319 \| L321 \| \| A325 \| Q326 \| E329 \|  \| | \| Q268 \| K271 \| R279 \| I319 \| \| --- \| --- \| --- \| --- \| \| L321 \|  \|  \|  \| |
| Binding sites 8 | \| I266 \| H281 \| M327 \| G332 \| \| --- \| --- \| --- \| --- \| | \| F231 \| D261 \| A263 \| A264 \| \| --- \| --- \| --- \| --- \| \| I266 \| Y268 \| E270 \| V280 \| \| H281 \| M327 \| S329 \| G332 \| \| A333 \| D334 \|  \|  \| | \| I266 \| H281 \| M327 \| G332 \| \| --- \| --- \| --- \| --- \| |
| Binding sites 9 | \| Y265 \| K271 \| R322 \| M324 \| \| --- \| --- \| --- \| --- \| | \| I263 \| Y265 \| E267 \| D270 \| \| --- \| --- \| --- \| --- \| \| K271 \| N272 \| Q275 \| D282 \| \| V285 \| H287 \| R322 \| M324 \| \| S326 \|  \|  \|  \| | \| Y265 \| K271 \| R322 \| M324 \| \| --- \| --- \| --- \| --- \| |
| Binding sites 10 | \| R329 \| M331 \| Y339 \| R341 \| \| --- \| --- \| --- \| --- \| | \| T236 \| D242 \| E274 \| M275 \| \| --- \| --- \| --- \| --- \| \| V327 \| R329 \| A337 \| D338 \| \| Y339 \| K340 \| R341 \|  \| | \| R329 \| Y339 \| R341 \|  \| \| --- \| --- \| --- \| --- \| |
| Binding sites 11 | \| A256 \| I258 \| Y260 \| E264 \| \| --- \| --- \| --- \| --- \| \| K266 \| N267 \| Q270 \| V272 \| \| V280 \| R317 \| M319 \| G324 \| \| Y327 \| R329 \|  \|  \| | \| D249 \| A252 \| I254 \| Y256 \| \| --- \| --- \| --- \| --- \| \| I258 \| M259 \| Y260 \| D261 \| \| K266 \| H269 \| V299 \| R301 \| \| M303 \| G308 \| A309 \| Y311 \| | \| A256 \| I258 \| Y260 \| K266 \| \| --- \| --- \| --- \| --- \| |

Hydrogen bond

Hydrophobic interaction

Hydrogen bond + Hydrophobic interaction

Hydrogen bond + Salt bridge

Hydrophobic interaction + Salt bridge

Hydrogen bond + Hydrophobic interaction + Salt bridge

**S5 Table. The binding free energy differences between the mutations and residues of designs and control in binding site 1 (kcal.mol^-1^).**

| **Design-1022** | | | **Design-3753** | | | **Design-6013** | | |
| --- | --- | --- | --- | --- | --- | --- | --- | --- |
| **Mutations** | **Residues** | **∆G_difference_** | **Mutations** | **Residues** | **∆G_difference_** | **Mutations** | **Residues** | **∆G_difference_** |
| T29K |  | -0.17 | T29K |  | -1.74 | T29K |  | -0.6 |
|  | Y32 | -0.6 | N51D |  | 6.9 | N51D |  | 4.6 |
|  | Y34 | 1.42 | S53T |  | -0.9 | N58C |  | 0.6 |
| N51D |  | 5.4 | N54S |  | 1.2 | R98Q |  | 1.8 |
| S53L |  | -1.3 | N58C |  | 0.8 |  | D99 | 1.4 |
| N58C |  | 0.8 |  | R98 | 2.9 | Y100K |  | 3.7 |
|  | R97 | -0.5 | Y100K |  | 0.8 |  | R101 | 3.5 |
|  | R98 | 1.9 |  | R101 | -4.4 |  | D103 | 5.1 |
|  | D99 | 2.5 |  | F102 | -0.9 |  | M104 | -0.7 |
| Y100K |  | 0.7 |  | D103 | -0.7 |  | D107 | -1.2 |
|  | R101 | -4.2 |  | M104 | -3.1 | T147S |  | 0.8 |
|  | D103 | -0.9 |  | G105 | -0.4 | S148D |  | 0.8 |
|  | D107 | 2.9 |  | D107 | -0.5 | Y152F |  | -1.2 |
| T147S |  | 0.2 | T147S |  | 0.4 | Y173L |  | -0.2 |
| S148D |  | 3.7 | S148D |  | 1 |  | S211 | 1.6 |
|  | Y150 | -0.3 |  | Y150 | -0.9 | R212A |  | 1 |
| Y152F |  | -0.9 | Y152F |  | -1.3 |  | D213 | 1.1 |
| Y173F |  | 0.6 | Y173F |  | -0.2 |  |  |  |
|  | E175 | -2.6 |  | E175 | -1.4 |  |  |  |
| R212A |  | 1.2 | R212A |  | 1.1 |  |  |  |
|  | L214 | 0.8 |  |  |  |  |  |  |
